# Decay of Genes Encoding the Oomycete Flagellar Proteome in the Downy Mildew *Hyaloperonospora arabidopsidis*

**DOI:** 10.1371/journal.pone.0047624

**Published:** 2012-10-15

**Authors:** Howard S. Judelson, Jolly Shrivastava, Joseph Manson

**Affiliations:** Department of Plant Pathology and Microbiology, University of California Riverside, Riverside, California, United States of America; Donald Danforth Plant Science Center, United States of America

## Abstract

Zoospores are central to the life cycles of most of the eukaryotic microbes known as oomycetes, but some genera have lost the ability to form these flagellated cells. In the plant pathogen *Phytophthora infestans*, genes encoding 257 proteins associated with flagella were identified by comparative genomics. These included the main structural components of the axoneme and basal body, proteins involved in intraflagellar transport, regulatory proteins, enzymes for maintaining ATP levels, and others. Transcripts for over three-quarters of the genes were up-regulated during sporulation, and persisted to varying degrees in the pre-zoospore stage (sporangia) and motile zoospores. Nearly all of these genes had orthologs in other eukaryotes that form flagella or cilia, but not species that lack the organelle. Orthologs of 211 of the genes were also absent from a sister taxon to *P. infestans* that lost the ability to form flagella, the downy mildew *Hyaloperonospora arabidopsidis*. Many of the genes retained in *H. arabidopsidis* were also present in other non-flagellates, suggesting that they play roles both in flagella and other cellular processes. Remnants of the missing genes were often detected in the *H. arabidopsidis* genome. Degradation of the genes was associated with local compaction of the chromosome and a heightened propensity towards genome rearrangements, as such regions were less likely to share synteny with *P. infestans*.

## Introduction

Flagella and their shorter relatives, cilia, are remarkably conserved in structure in all major eukaryotic groups [1]. This suggests that these motility and sensory organelles existed in the last eukaryotic common ancestor, LECA. The appearance of microtubular flagella, along with other cytoskeletal components, are hallmarks of the transition from prokaryote to eukaryote [2]. Flagella have been influenced during evolution by the acquisition, loss, or modification of genes. For example, the major component of eukaryotic flagella, tubulin, is thought to have evolved from the prokaryotic cell division protein ftsZ [2]. In addition, evolution of a cytoplasmic pathway for assembling flagella in protozoa such as *Plasmodium* has been linked to the loss of intraflagellar transport proteins, which in other species deliver building blocks from basal bodies to flagellar tips [3].

The most dramatic example of evolutionary change involving flagella is the loss of the organelle. This occurred during the evolution of plants, slime molds, most fungi, and some oomycetes [1], [4], [5]. It is reasonable to propose that in such cases an initial mutation arose that impaired the formation of flagella, followed by relaxed selection against defects in other genes. Similar events are well-described in bacterial symbionts, where pathway losses were linked to chromosomal rearrangements, deletions, repetitive element proliferation, pseudogene and gene remnant accumulation, and reduction in genome size [6], [7]. Related events in eukaryotes have been associated with gene truncation, mutation, or deletion along with transposable element activity [8], [9], [10].

The existence of species that lost the ability to form flagella can help define proteins required for the organelle. By searching for genes shared only by flagellates, prior studies identified between about 75 and 250 candidates depending on the species examined [11], [12], [13], [14]. In addition, proteomic studies of the organelle identified up to about 300 proteins [15], [16], [17], [18]. Not all proteins found by proteomics are necessarily specific to flagella, and some could be contaminants. Many were nevertheless shown to have a flagellar role by RNAi, to be expressed during flagella formation, or to localize to the organelle [17], [19], [20]. Central features of flagella include microtubules that usually form a 9+2 configuration of outer and inner doublets, radial spokes that join the outer and inner doublets, dynein arms between the doublets, and the basal body. Flagellar structure can vary between species, for example mammalian sperm contain unique fibers peripheral to the 9+2 axoneme, and trypanosomes contain a novel paraflagellar rod [1]. Combined with the fact that some flagellar proteins may be retained in nonmotile or aciliated species if they serve other roles, there is value to integrating comparative genomics and proteomics across multiple species.

To understand further the diversity of eukaryotic flagella and mechanisms of their elimination from some lineages, here we focus on the oomycetes *Phytophthora infestans*, the potato blight agent, and *Hyaloperonospora arabidopsidis*, a downy mildew pathogen of *Arabidopsis thaliana* [21]. Like most oomycetes, *P. infestans* produces a biflagellated life-stage called the zoospore that helps propagules reach optimal infection or colonization sites [22]. Despite its fairly close taxonomic affinity to *P. infestans, H. arabidopsidis* fails to form zoospores, and in a preliminary study we reported that some genes for flagella proteins were absent from the latter's genome [23]. To explore this further, in this paper we report the identification of 257 candidate flagella genes from *P. infestans* based on comparative genomics and RNA expression studies, and show that 81% lack orthologs in *H. arabidopsidis*. The loss of flagella from the downy mildew does not seem to be a very ancient event since gene remnants were found for about one-fifth of genes.

## Results

### Candidate *P. infestans* flagellar proteins from comparative genomics

Two recent studies reported finding about 100 orthologs of known flagella genes in oomycetes [13], [24]. To search more thoroughly for candidates from *P. infestans*, sequences were collected from prior studies of diverse species. These proteins included 95 found by proteomic analysis of flagella from *Chlamydomonas reinhardtii* [15], 195 identified by comparative genomics as being in flagellates such as *C. reinhardtii* but not *H. arabidopsidis* [11], 106 from genomics and proteomics analysis of *Trypanosoma brucei* [12], [17], 182 from a study of *Naegleria gruberi* [13], 148 predicted to be in metazoan cilia [25], 51 downregulated in a cilia-lacking mutant of *Caenorhabditis elegans* [26], 102 from a proteomics study of *Tetrahymena thermophila* basal bodies [27], and other proteins reported to reside in flagella or cilia [26], [28], [29], [30], [31]. These candidate flagella components represented 460 nonredundant proteins.

*P. infestans* was searched for orthologs to the 460 sequences employing the reciprocal best hit method using Blastp. Ortholog assignment was supplemented by phylogenetic analysis when multiple closely-matching proteins were detected. To reduce false negatives due to erroneous gene models, candidates lacking orthologs in *P. infestans* were searched against its assembly as well as the related species *Phytophthora sojae*. A total of 257 orthologs were thus identified, including one from an uncalled gene in the original genome study [32]. The common names, *P. infestans* gene model numbers in the Broad Institute database, and predicted functions of each gene are listed in Table S1.

### Distribution of orthologs in other eukaryotes

**Figure 1:**
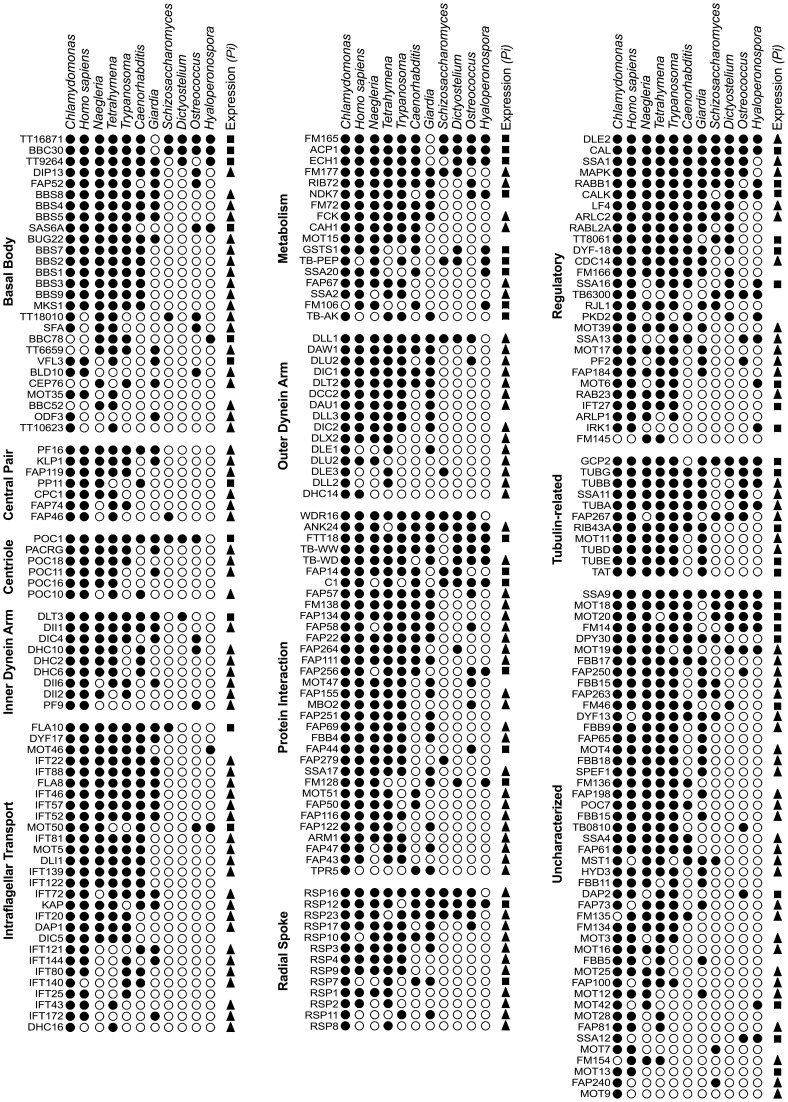
Phylogenetic distribution of flagella-associated components detected in *P. infestans*. Orthologs (black circles) were identified from seven eukaryotes capable of forming flagella or cilia (*C. reinhardtii, H. sapiens, N. gruberi, T. thermophila, C. elegans, G. lamblia*) and four that do not make the organelle (*S. pombe, D. discoideum, O. tauri, H. arabidopsidis*) using *P. infestans, C. reinhardtii, and N. gruberi* proteins as query sequences. Searches were performed using OrthoMCL and supplemented with the reciprocal best hit approach. Genes are grouped by functional categories, although some categories may overlap.

The association of the genes having orthologs in *P. infestans* with flagella or cilia was explored by examining eleven other eukaryotes (Fig. 1). The species examined include six that form motile flagella or cilia (*C. reinhardtii, H. sapiens, N. gruberi, T. thermophila, T. brucei, Giardia lamblia*), *C. elegans* which forms only non-motile sensory cilia, and *Dictyostelium discoideum, Schizosaccharomyces pombe, Ostreococcus tauri*, and *H. arabidopsidis* which produce neither cilia or flagella. Phylogenetic analyses indicate that the last species is related closely to *P. infestans* [33].

Most of the predicted *P. infestans* flagella-associated proteins, about 200, were conserved in *C. reinhardtii*, humans, *N. gruberi, T. thermophila, and T. brucei*. This number was reduced in *C. elegans* and *G. lamblia* to 117 and 124, respectively, which reflects the lack of motile cilia in the nematode and the compaction of the *G. lamblia* genome [34]. About 78% of the proteins lack orthologs in the non-flagellates.

As shown in Fig. 1, *P. infestans* encodes members of all major flagellar protein functional categories as defined by studies of models such as *C. reinhardtii*. The proteins in *P. infestans* include 27 that reside within or attach to the basal body, seven central pair proteins, all five expected tubulins (α, β, γ, δ, ε), 24 proteins representing heavy, intermediate, and light chain dyneins, and other proteins with roles in cellular regulation, metabolism, protein-protein interactions, and other functions. Also present are 28 proteins involved in intraflagellar transport. These include each IFT protein described for *C. reinhardtii*, along with the kinesins that work with IFT factors to move proteins along the axoneme. *P. infestans* also encodes orthologs of 13 of the identified radial spoke proteins of *C. reinhardtii*. Only one RSP4-like protein is encoded by *P. infestans*, like most eukaryotes except *C. reinhardtii* where the gene is duplicated [35]. Of the proteins expressed from single genes in the green alga, six come from duplicated genes in *P. infestans*, namely DAU1, DHC10, DHC14, DYF13, FAB57, and FBB15, and RIB72. Four *P. infestans* genes correspond to centrin, which is single-copy in *C. reinhardtii* (DLE2) but also multicopy in humans [36].

Of orthologs found in non-flagellates, most common are metabolic proteins. Examples include the FM165 acyl transferase, which was detected in *S. pombe*, *D. discoideum, O. tauri*, and *H. arabidopsidis*, and the FM177 oxidoreductase, which was found in the first two species. Such proteins presumably play multiple roles, some of which persist in non-flagellates. A second class of retained proteins include those that mediate protein-protein interactions or protein maturation, such as RSP16, which encodes a Hsp40 chaperone, and RSP12, which encodes a peptidyl-prolyl isomerase.

Forty-one sequences from the *C. reinhardtii* flagellar proteome were not detected in *P. infestans*; most flagellates also lack these proteins (Fig. S1). For example, RSP5 was not detected in humans, *N. gruberi, T. thermophila, T. brucei, G. lamblia*, or *C. elegans*. Also absent from *P. infestans* were many flagella-associated proteins that had been identified in *N. gruberi* and *T. brucei*, but shown before to be absent from most other flagellates. For example, *P. infestans* lacks orthologs of 26 of the 30 proteins from the paraflagellar rod of *T. brucei*, a structure that is unique to trypanosomes. In contrast, the genes found in *P. infestans* have functions not specific to flagella, such as a calmodulin (*P. infestans* gene model PITG_06514), adenylate kinase (PITG_18377), and phosphatidylinositol 3-related kinase (PITG_02495).

Since alveolates and oomycetes are proposed to have a common ancestry as chromalveolates [37], it was interesting to note that several proteins present in *P. infestans* and *T. thermophila* were absent from most of the other species. These included three found also in *N. gruberi* (FM145, FM154, BBC52), and two basal body proteins shared only with *T. thermophila*.

Only 44 of the *P. infestans* proteins had detectable orthologs in *H. arabidopsidis*. The fate of the missing genes in this flagella-lacking relative of *P. infestans* is the focus of the last half of this paper.

### Most *P. infestans* flagellar protein candidates are induced in spores

Transcript levels for the genes during the *P. infestans* life cycle were measured to test whether their expression patterns were correlated with the timing of zoospore development. This involved microarray and quantitative RT-PCR (qRT-PCR) analysis of nonsporulating hyphae; hyphae early in sporulation, when immature sporangia are just starting to appear; sporangia purified from the hyphae; and motile zoospores released from sporangia, 2.5 hr after cold-treating the sporangia to induce germination. Data were obtained for 224 genes. These included 116 from microarrays and 108 from qRT-PCR (the microarrays only represented part of the transcriptome; [38],[39]). Both qRT-PCR and microarray data were obtained for several genes, with both methods yielding comparable results.

**Figure 2:**
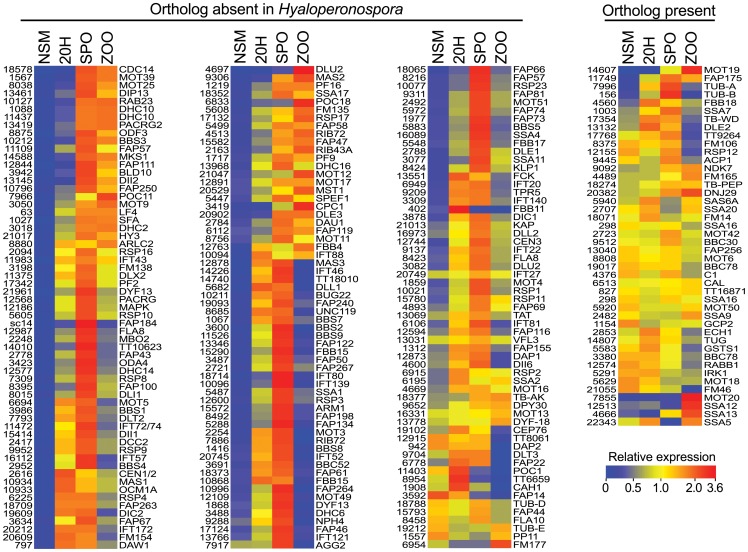
Expression of predicted flagella-associated genes from *P. infestans*. RNA levels were measured in nonsporulating mycelia (NSM), cultures 20 hours after being induced to sporulate (20H), purified ungerminated sporangia (SPO), and swimming zoospores (ZOO). Panels are split based on the presence or absence of orthologs in *H. arabidopsidis*. Numbers to the left of each image represent the accession number from the Broad Institute database (trimmed of the PITG prefix), and lettering to the right of each image represents the common names of the gene products as listed in Table S1.

Of the 224 genes thus measured, 172 and 149 were up-regulated by >2.5-fold and >5-fold, respectively, in the early sporulation, sporangia, or zoospore stages compared to nonsporulating hyphae. This is illustrated in Fig. 2, where the data are separated into panels based on whether orthologs were detected in *H. arabidopsidis*. The data are also summarized in Fig. 1, where upwards-pointing arrows denote up-regulated genes.

Most up-regulated genes (induced by >2.5-fold) were induced early in sporulation, reached highest levels in ungerminated sporangia, and declined in zoospores. Their induction during sporulation is consistent with data from inhibitor studies that suggest that most flagella proteins are preformed in sporangia [40]. This was the case, for example, for most genes encoding the principal components of the axoneme and its assembly machinery including α- and β-tubulin, all 15 genes encoding IFT proteins, all but one of the 22 outer and inner dynein arm protein genes, all flagella-associated kinesins, and all but two radial spoke proteins. Another induced class of genes encoded tubulin-tyrosine ligases, which regulate the recruitment of microtubule-interacting proteins to axonemes [41]. Three tubulin-tyrosine ligases (PITG_2721, PITG_03077, PITG_08756, corresponding to FAP67, SSA11, and MOT11, respectively) showed nearly identical induction patterns, rising about 3-fold in early sporulation compared to nonsporulating hyphae and 10-fold in sporangia compared to hyphae.

A notable difference between induced genes concerned whether their transcripts stayed abundant in swimming zoospores. RNA for about three-quarters declined in that stage compared to sporangia, while one-quarter stayed high. This might reflect differences in the need for *de novo* synthesis after zoosporogenesis to maintain flagella function, or might be a less biologically relevant reflection on their mRNA stability. Examples of genes that had reduced RNA levels after zoospore release included many involved in forming structural components of the axoneme, including all but two of the dyneins, all radial spoke proteins, the tubulin-tyrosine ligases, and most IFT proteins. While RNAs for most genes were still at higher levels in zoospores than hyphae, PITG_20745 (IFT52) and PITG_10096 (IFT139) quickly declined to levels seen in hyphae.

Several genes involved in energy homeostasis were also induced in one or more spore-associated stages, and many maintained high mRNA levels in zoospores. One example is PITG_03419 (CPC1), which encodes a central pair adenylate kinase (reaction: ADP + ADP → AMP + ATP). Its RNA levels were three times higher in zoospores than sporangia, which may reflect a role in preserving ATP levels, or ATP/ADP ratios which affect dynein activity [42]. A second adenylate kinase, encoded by PITG_18379, also had the highest levels in zoospores. A gene encoding a nucleoside diphosphate kinase regulatory subunit, PITG_04513 (NDK), also had the most RNA in zoospores (reaction: GTP + ADP → GDP + ATP). The NDK catalytic subunit (genes PITG_03634 and PITG_07886; RIB72) was also elevated in zoospores compared to hyphae, although their highest levels were in sporangia. A phosphagen kinase encoded by PITG_13551 (FCK) was induced >10-fold during early sporulation, but declined in subsequent stages.

Diversity in expression patterns was also observed for genes encoding the mastigoneme (flagella tinsel) proteins, represented by PITG_09306, PITG_10934, and PITG_12878. All had very low RNA levels in nonsporulating hyphae. PITG_10934 and PITG_12878 were induced >10-fold during early sporulation, however PITG_09306 transcripts were not induced strongly until the sporangia stage. The differences in expression patterns may reflect the fact that the proteins form distinct parts of the mastigoneme, *i.e*. shaft versus basal region, which possibly assemble at different times [29].

### Transcript patterns correlate with gene absence from aflagellates

The *P. infestans* orthologs of genes missing from species lacking flagella were almost always induced during sporulation, which is consistent with the genes' role in the organelle. This can be seen in Fig. 2 by comparing the panels of genes retained and lost in *H. arabidopsidis*, and Fig. 1 where spore-induced genes are marked by arrows. Of the genes lacking orthologs in *H. arabidopsidis*, 92% were up-regulated during sporulation or in spores, while only about 20% of genes having orthologs in *H. arabidopsidis* were induced. Most *P. infestans* orthologs of genes absent from *D. discoideum, O. tauri*, and *S. pombe* were also up-regulated.

About half of proteins retained in the non-flagellates had up-regulated orthologs in *P. infestans*. These include α- and β-tubulin (PITG_7996 and PITG_00156), which comprise a major part of flagella but have many other roles in growth. Another example is the *P. infestans* ortholog of *T. brucei* protein TB10.61.0160 (PITG_17354, named TB-WD in Fig. 1), which is a WD-domain protein. The human ortholog has the highest expression in flagella or cilia-forming cells, but is also expressed in other cell types, which suggests that it has multiple roles [43].

Orthologs of the about half of the genes retained in the non-flagellates were not induced in spores. Two examples are γ-tubulin (PITG_14807, TUBG in Fig. 1) and γ-tubulin associated protein GCP2 (PITG_1154), which acts at microtubule organizing centers maintained in non-flagellates, such as spindle poles or the centrosome. An additional example is RSP12 ortholog PITG_12155, which is predicted to be a peptidyl-prolyl isomerase.

About 8% of the *P. infestans* genes lacking orthologs in non-flagellates were not up-regulated in spores, since some probably act throughout the life cycle. This was also observed in *C. reinhardtii*, where many flagella-associated genes are not induced during flagellar regeneration [14]. One example is PITG_20749, which encodes Rab GTPase IFT27. This protein is unique among IFT factors in that it appears to be needed for general growth based on knockdown studies [44]. Many of the other non-induced, aflagellate-retained genes have roles in centrioles or basal bodies, which are structurally similar and in many (but not all) species interconvert during the cell cycle [45]. Examples include PITG_1877 and PITG_19212, which encode δ- and ε-tubulin, respectively, and the VFL3 ortholog PITG_13031, which have demonstrated roles in both organelles [46], [47]. Orthologs of others such as PITG_00827, PITG_09512, and PITG_19017 (TT16871, BBC30, FTT18) have been localized to basal bodies and might also have a centriolar role [27].

Most of the few remaining proteins that are expressed in all life-stages and retained in non-flagellates have no characterized function. A few have activities for which a flagella-specific role is challenging to conceptualize, such as PITG_05629, which encodes a non-canonical polyA polymerase, and PITG_02853, which encodes enoyl-CoA hydratase.

### Fate of flagellar genes in *H. arabidopsidis*

**Figure 3:**
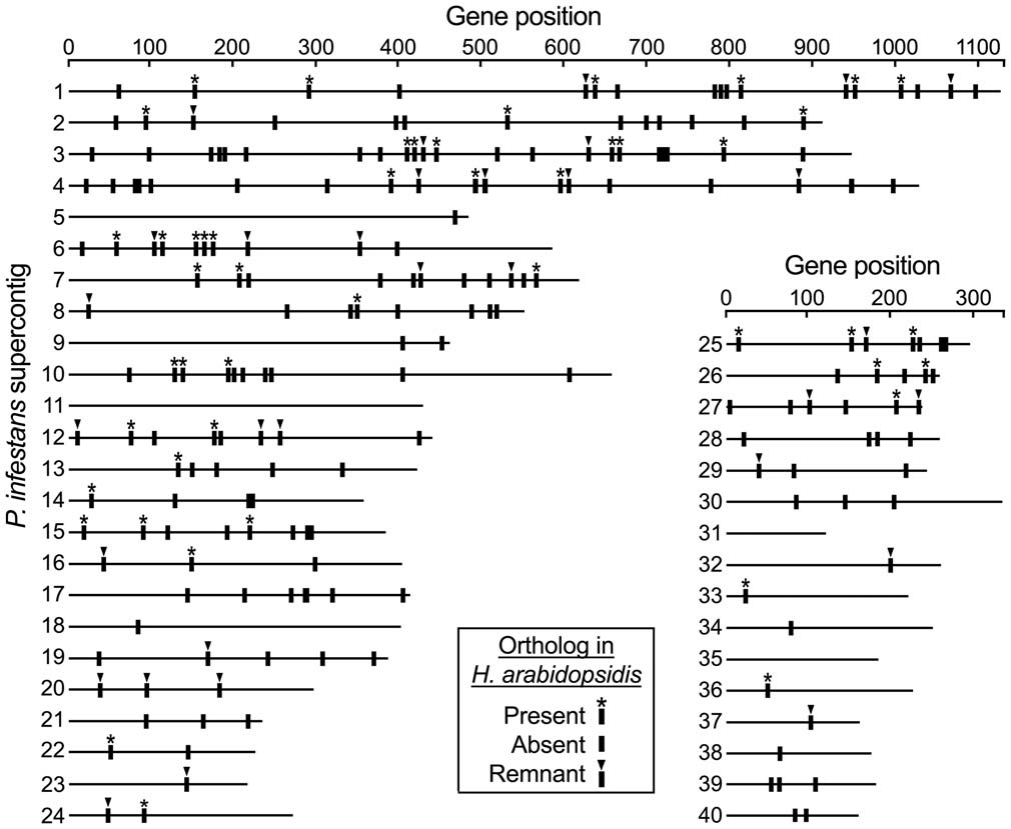
Location of predicted flagella-associated genes from *P. infestans*. Shown are maps of the 40 largest supercontigs from the assembly, with positions of genes marked by vertical bars. An asterisk above the bar indicates that the gene is intact in *H. arabidopsidis*, and an arrow denotes the presence of a gene remnant in *H. arabidopsidis*.

To initiate an investigation into how the flagellar genes were lost from *H. arabidopsidis*, their locations in *P. infestans* were mapped on the latter's major supercontigs (Fig. 3). The flagellar genes were not clustered in *P. infestans*, with most residing within gene-rich islands. Previous studies showed that about 70% of all *P. infestans* genes and 90% of those with orthologs in other oomycetes reside in such islands, in which gene order is often conserved between *Phytophthora* spp. and *H. arabidopsidis* [23], [32], [48]. In our analysis of 30 gene-rich clusters containing a total of 696 and 920 genes from *H. arabidopsidis* and *P. infestans*, respectively, 46% of *H. arabidopsidis* genes shared some degree of synteny with their *P. infestans* counterparts, as they were found in similar locations within each gene-rich cluster.

The lower amount of downy mildew genes in these clusters compared to *P. infestans* is consistent with the former's smaller total gene content, 14,543 versus 17,797, of which only about 7500 are orthologs [23]. Based on the positions of the flagellar genes in *P. infestans* and the commonly conserved gene order between the two species, it seems unlikely that elimination of the flagellar genes from the downy mildew involved a major catastrophic rearrangement, such as the loss of a chromosome arm.

Shared synteny between *P. infestans* and *H. arabidopsidis* was next exploited to search for remnants of the flagellar genes in the latter. Matches with significant *E* values had not been detected in our genome-wide searches, so a more sensitive exploration was pursued by studying the intergenic regions of *H. arabidopsidis* that matched locations of the genes in *P. infestans*. Shared synteny was detected for 179 regions containing the 257 flagella genes; synteny was declared if at least one of the four genes upstream of a targeted region, plus at least one of the four downstream genes, were near each other in the two species. The value should be taken as a rough estimate of the extent of shared synteny, since only draft genomes are available for analysis and gaps would cause false negatives. For comparison, shared synteny between *P. infestans* and *P. sojae* was observed for 83% of the regions containing flagellar genes.

**Figure 4:**
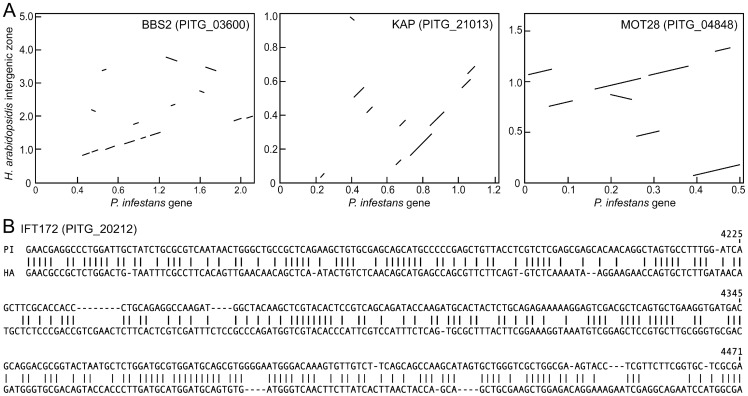
Examples of gene remnants in *H. arabidopsidis*. **A,** Comparisons of three *P. infestans* genes representing orthologs of *C. reinhardtii* BBS2, KAP, and MOT28 and the three corresponding intergenic regions from *H. arabidopsidis*. The images are redrawn from the dot matrix output of NCBI Blast 2.2.26, with diagonals representing regions of similarity. **B,** Alignment of portion of remnant of IFT172 gene from *H. arabidopsidis* (HA) with *P. infestans* gene PITG_20212 (PI). Numbers at right indicate the position within the *P. infestans gene*. The *H. arabidopsidis* sequences correspond to Scaffold 1, positions 1119536 to 1119160, of assembly version 8.3. The total aligned segment spanned 1597 nt, but only 373 nt is shown.

Within the 179 regions that showed shared synteny, gene remnants were detected in 37 cases using LALIGN and TBLASTN as described in Materials and Methods. The authenticity of each match was supported by calculations of false discovery rates, and by assessing the statistical significance of each alignment using randomly shuffled sequences (Table S2). Compared to the *P. infestans* genes, the *H. arabidopsidis* sequences typically contained multiple base changes including indels that changed the reading frame, as well as rearrangements such as larger deletions and inversions. This is illustrated in Fig. 4 for four selected genes; the total results are tallied in Table S2, and the 37 alignments are shown in Fig. S2. The regions of alignment between the *P. infestans* gene and *H. arabidopsidis* remnant usually spanned only part of the intact gene (42% on average), ranged in length from 83 to 2713 nt, and averaged 57% nucleotide identity. In comparison, flagella candidate genes having orthologs in both species averaged 83% nucleotide identity in alignments.

Interestingly, the elimination of a gene from *H. arabidopsidis* was more prone to be associated with a loss of shared synteny with *P. infestans*. Of the genes retained in *H. arabidopsidis*, about 82% showed shared synteny between the two species (based on the genes flanking the flagella gene) compared to only 66% for regions that lost the gene. The difference is significant by Fisher's Exact Test, with *P* equaling 0.02 (0.01 when recalculated for synteny with *P. sojae*).

The value of focusing searches for remnants to the region of *H. arabidopsidis* predicted to formerly bear the flagella gene is demonstrated by comparing the success here with a prior study [23]. That earlier approach checked *H. arabidopsidis* for sequences resembling 84 *C. reinhardtii* flagella genes having orthologs in *Phytophthora*. Due to weaknesses in the genome assembly available at that time, linkages between genes predicted to flank the former flagella gene in *H. arabidopsidis* were detected in only eight cases, and none of the intervening regions contained remnants. Here we used a dramatically improved assembly in which linkages between flanking genes were detected 69% of the time. Combined with expanding the total analysis from 84 to 257 genes, this enabled the 37 remnants to be detected.

### Gene loss is associated with chromosomal compaction

**Figure 5:**
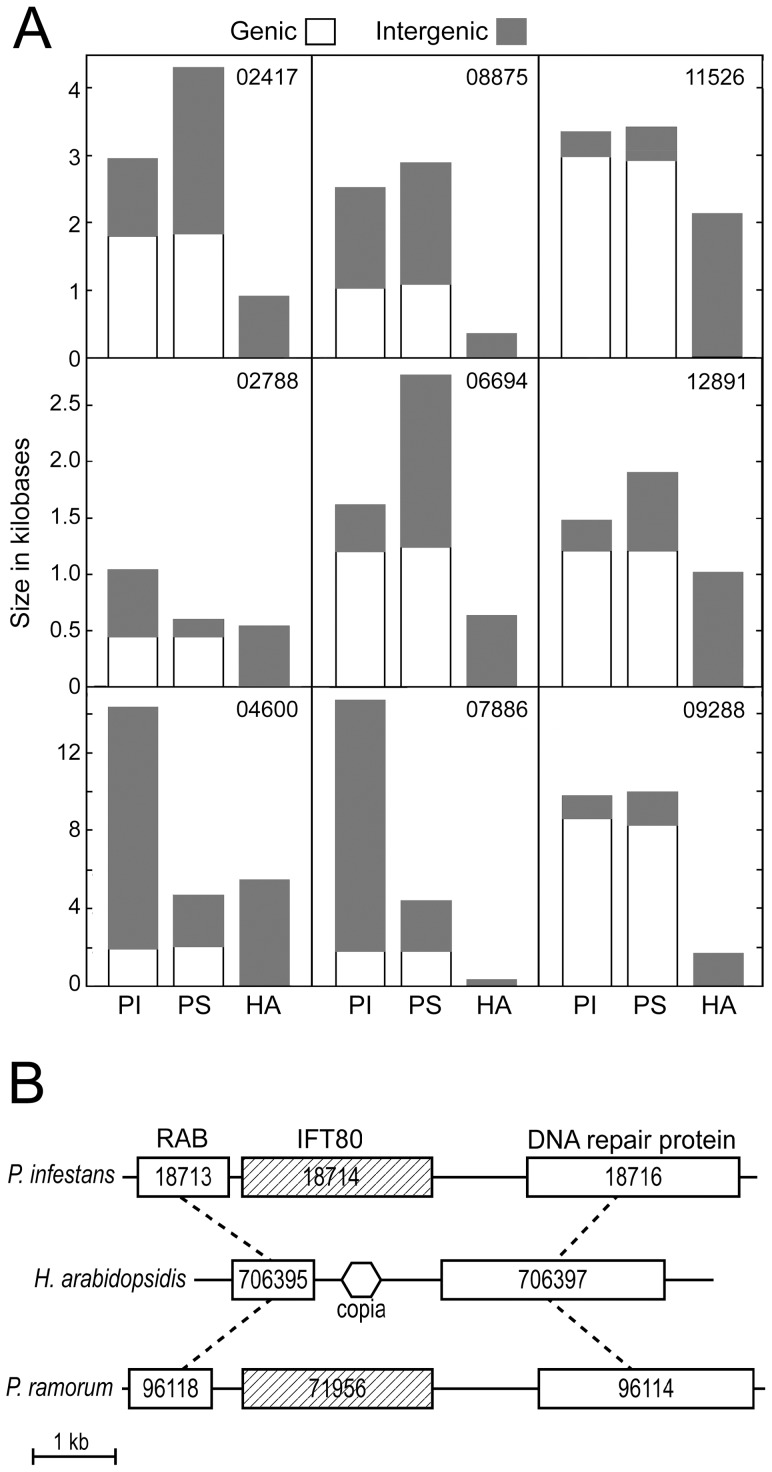
Examples of loci experiencing gene loss in the downy mildew. **A,** Sizes of genic (white bars) and intergenic (dark bars) regions in *H. arabidopsidis* (HA), *P. infestans* (PI), and *P. sojae* (PS). Genic regions include the entire predicted primary transcript, and the number of the corresponding *P. infestans* gene is marked in the upper right-hand corner of each panel. **B,** *H. arabidopsidis* locus experiencing loss of IFT80 gene, compaction, and transposable element insertion. Dashed lines indicate flanking orthologs in *P. infestans* and *P. ramorum*, boxes represent genes (with database number indicated), and the hexagon in the *H. arabidopsidis* map represents the location of a Copia-like sequence. Maps are drawn to scale using corrected gene models, and illustrate how the region between the RAB and DNA repair protein genes is reduced from 3.5 kb in *Phytophthora* to 1.5 kb in *H. arabidopsidis*.

Gene loss in *H. arabidopsidis* was most often associated with a reduction in size of the affected region of the chromosome. This conclusion was drawn from cases where the two genes flanking the flagellar gene had the same order in *P. infestans, P. sojae*, and *H. arabidopsidis*. This is illustrated in Fig. 5A for nine representative genes. For example, orthologs of PITG_08875 had genic and intergenic regions, respectively, of 1.0 and 2.8 kb in *P. infestans* and *P. sojae*, which was reduced to 359 nt in *H. arabidopsidis*. Similarly, the 9 kb interval containing PITG_09288 was reduced to 1.7 kb in *H. arabidopsidis*. Interestingly, gene remnants were always found when the intergenic region in *H. arabidopsidis* was not very reduced compared to *Phytophthora* (PITG_04600, PITG_11526, PITG_12891). This suggests that the rate of gene loss through chromosomal deletion was higher than by base-by-base degeneration.

The distance between genes flanking the flagellar locus was smaller in *H. arabidopsidis* than *P. infestans* in 78% of cases; a similar conclusion was drawn from the prior study of eight loci [23]. Interestingly, this trend in intergenic distances is opposite to that observed for genes that share synteny but have functions unrelated to flagella. Of 120 gene pairs selected randomly from 40 different regions of their genomes, intergenic regions were smaller in *H. arabidopsidis* than *P. infestans* only 31% of the time, with median sizes of 310 and 510 nt, respectively. The loss of the flagella gene therefore appeared to facilitate the compaction of the *H. arabidopsidis* genome beyond what would be expected if only the flagella gene and its transcriptional regulatory sequences had been lost.

Oomycete genomes contain a large amount of transposon and retroelement-like sequences, but such elements did not appear to have the predominant role in eliminating flagellar genes. In a search of 32 intergenic regions that formerly contained a flagella gene, repetitive sequences were detected only in the case of IFT80-encoding gene PITG_18714. As illustrated in Fig. 5B, the 3.5 kb region containing the IFT80 gene and flanking noncoding DNA was reduced to 1.5 kb in *H. arabidopsidis*. This included about 400 nt of a Copia-like sequence having 46 relatives in the *H. arabidopsidis* genome, based on BLAST with a *E* = 10^−9^ threshold. It is possible that transposable elements had a greater role than predicted by this analysis, which was biased by its focus on cases where gene order was conserved.

## Discussion

Of the 257 *P. infestans* proteins identified as resembling flagellar components of other eukaryotes, most are connected firmly to the biology of that organelle since 77% of the corresponding genes are up-regulated during the life-stages when zoospore components are synthesized, and 82% are absent from the non-flagellated sister taxon *H. arabidopsidis*. While it was not our intention to identify the complete flagellar proteome of *P. infestans*, the 257 proteins are close in number to the roughly 250 estimated by 2-dimensional gel analysis to comprise the *C. reinhardtii* flagellar axoneme [49]. Reflecting diversification of flagella during the eukaryotic radiation, about 45 proteins assigned to *C. reinhardtii* flagella lacked orthologs in *P. infestans*, while 7 *P. infestans* sequences lacked orthologs in *C. reinhardtii*. The latter were likely part of the ancestral organelle, since they match proteins in other lower eukaryotes such as *N. gruberi*. Oomycetes may also have evolved novel flagellar proteins, but identifying them is problematic due to an inability to purify enough flagella for analysis.

The highest confidence flagella-focused dataset from *P. infestans* includes about 185 proteins, based on the fraction that were up-regulated during spore formation and lacked orthologs in aflagellates. Combining both criteria reduces false positives, but at the expense of excluding proteins with multiple functions. This is especially true for proteins that may reside in both basal bodies and centrioles, which in many but not all species interconvert during the life cycle [1], [45]. While most basal body proteins have unknown functions, hints may come from their patterns of expression. Constitutive genes may encode proteins present in both basal bodies and centrioles, such as PITG_09512, which encodes the ortholog of a *T. thermophila* basal body protein, BBC30 [27]. In contrast, up-regulated genes might influence the disappearance of centrioles and genesis of basal bodies during zoospore development. An example is sporulation-induced gene PITG_14588 (MKS1), which encodes the ortholog of a human protein needed for the migration of centrosomes to the sites of basal body formation [50]. Although most oomycetes are known to use centrioles to nucleate the spindle at mitosis, their fate during the cell cycle and flagella-forming stages is not well-understood [51]. Strikingly, *H. arabidopsidis* lacks orthologs of all centriole-associated proteins covered by this study, including POC1 which is otherwise widely distributed in eukaryotes. It is unknown whether *H. arabidopsidis* has centrioles.

The up-regulated *P. infestans* genes exhibited diverse kinetics of mRNA induction and persistence, which likely reflects the role of each gene and biology of the spores. Sporangia are an intermediate between hyphae and zoospores. While sporangia can make zoospores soon after maturation, sporangia may remain quiescent for days until zoosporogenesis is stimulated by environment (cold temperatures and free water). Zoospore release can take less than an hour after stimulation, which is consistent with studies using actinomycin-D and cycloheximide that suggested that sporangia already contain all necessary proteins [40], [52]. Matching that earlier finding is our observation that virtually all genes for axonemal proteins (dynein arms, radial spokes, etc.) were induced early in sporulation. Why most RNAs persist through later stages may be explained by the need to replenish proteins broken down during sporangial quiescence, and to preserve motility, which can last a day under ideal conditions. Many late-induced genes may not be needed directly to form zoospores but may instead enable prolonged swimming and chemotaxis. Adenylate kinase PITG_03419 (CPC1), for example, may help maintain ATP levels during the motile period. The need for such functions may extend into subsequent life-stages, such as during encystment of the zoospore, extension of a germ tube from the cyst, or appressorium formation [22].

Why and how the zoospore stage was lost from *H. arabidopsidis* is unknown. The ability of most oomycetes to form zoospores is considered to aid survival by helping them reach nutrients or optimal host infection sites. However, zoospores were lost several times during oomycete speciation, particularly in obligately pathogenic clades such as *Hyaloperonospora, Bremia*, and most *Peronospora* spp. [53]. Aflagellates germinate by extending a hyphal tube directly from the asexual spore (conidium), and this also occurs in *Phytophthora* when conditions are too warm to favor zoospore release [22]. Presumably the need to maintain genes for both germination pathways represented a heavy load for some obligately pathogenic oomycetes, providing positive selection for their loss. In this regard, the elimination of zoospores parallels the trend of metabolic pathway loss seen in many obligately pathogenic bacteria and protists [7], [54]. It should be noted that zoospores are maintained in some obligately pathogenic oomycetes, including the *Albugo* white rusts and certain downy mildews within *Peronospora*.

One may speculate that destruction of the pathway began in one allele of a flagellar gene that experienced a spontaneous mutation or insertion of a transposable element. The latter are common in most oomycete genomes, and have influenced the evolution of several gene families; *H. arabidopsidis* has a 100 Mb genome, with 42% repetitive DNA content [23], [32], [55]. Considering that oomycetes are diploid, how the remaining functional allele was lost is intriguing. Loss of heterozygosity due to mitotic crossing-over appears frequent in some oomycetes [56], but this might have been ecologically deleterious if the elimination of the remaining functional allele was the result. Alternatively, the first step in zoospore loss could have been a dominant-negative regulatory mutation.

Regardless of the nature of the event that initiated zoospore loss from *H. arabidopsidis*, examining the fate of its remaining flagellar genes was of interest in light of the complexity of oomycete genomes, and the dearth of information about their evolution outside of effector families, retroelements, and occasional gene fusions [23], [57], [58], [59]. A fair degree of shared synteny between *Phytophthora* and *H. arabidopsidis* allowed us to identify remnants of many flagella genes and observe a higher propensity for loss of synteny at the affected regions. One factor maintaining gene order in oomycetes is probably their small intergenic distances, most commonly 400–500 nt; this likely restricts the viability of illegitimate recombination events such as unequal crossovers or transposon insertions. Degradation of a flagella gene presumably results in a larger target for viable recombination.

We also observed compaction of the *H. arabidopsidis* genome at most affected loci. Such changes are estimated to account for a reduction of about 1 Mb; overall shrinkage is likely greater since some metabolic genes, protein kinases, and effectors are also reported to be absent in *H. arabidopsidis* [23], [60]. Most other obligately parasitic eukaryotes such as *Cryptosporidium* and *Entamoeba* have more dramatically reduced genomes as a consequence of gene loss or fusion, intron reduction, and a diminution of intergenic distances [54]. Compaction, however, is not restricted to obligates. For example, the *O. tauri* genome is much smaller than its relatives [61].

It is interesting to note that the loss from *H. arabidopsidis* of a gene seemed to be a better predictor of its flagellar role than whether *O. tauri* lacked an ortholog of a *C. reinhardtii* gene; the fraction of retained flagellar genes was 35% greater in *O. tauri* than in the downy mildew. A recent survey showed that other plants that lack a flagella stage have also retained multiple flagella-associated proteins [62]. Retention of a flagella gene is likely explained by its acquisition of additional roles prior to the loss of the organelle. The fraction of retained genes also may reflect the time of divergence of *H. arabidopsidis* and *O. tauri* from their flagellated sister taxa, as well as from plants and the Stramenopile kingdom, which includes oomycetes, from their shared ancestor. Plants and Stramenopiles diverged more than one billion years ago during the Mesoproterozoic era, the two algae from each other 0.5 to 1 billion years ago during the Neoproterozoic (although flagellar loss is probably more recent), and downy mildews from *Phytophthora* less than 65 million years ago with oomycetes themselves having a Neoproterozoic origin [5], [63], [64]. Our understanding of oomycete evolution is still developing, and current phylogenetic schemes nest many of the non-flagellated plant pathogens within *Phytophthora* [33]. Dating the oomycete radiation is challenging in the absence of a clear fossil record, and it is interesting to consider that gene remnants as reported here for *H. arabidopsidis* may inform us about the timing of key events. The fact that gene remnants can still be detected suggests that flagella loss in *Hyaloperonospora* was relatively recent.

## Materials and Methods

### Identification of flagellar protein genes

Candidate proteins from the sources cited in Results were used to search a *P. infestans* protein database using BLASTP. This was performed on a local server or with tools provided for *P. infestans* at the Broad Institute of Harvard and MIT (www.broadinstitute.org, assembly v. 2). The veracity of ortholog assignments was tested using the reciprocal best hit method, by comparing *P. infestans* hits to databases of proteins from *C. reinhardtii* (http://genome.jgi-psf.org, version 3), *Tetrahymena* (ciliate.org, TIGR 2006 version), *T. brucei* database (tritrypdb.org, versions 2.5 and 3), or other species at Genbank. In the summary in Table S1, the common name of the *C. reinhardtii* ortholog as informed by *ChlamyCyc* (chlamyto.mpimp-golm.mpg.de) is used, except for dyneins that were recently renamed [65].

If several strong hits were obtained in *P. infestans*, then the sequences were compared phylogenetically to probe orthology further. This was also done whenever the BLASTP *E* value was below 10^−15^, which was common for some short proteins. This involved performing alignments using the SEAVIEW implementation of MUSCLE followed by PhyML to generate maximum likelihood trees [66]. In a few cases the *P. infestans* gene appeared to have undergone a duplication; after excluding the possibility that the duplication was an assembly artifact, both hits were placed on the list of candidate flagellar proteins, annotated with suffixes a and b. If no hits were detected against *P. infestans* proteins, then before concluding that the gene was absent, the test sequences were compared to the *P. infestans* assembly to check for unannotated genes and to gene models from *P. sojae* using the database at Virginia Bioinformatics Institute (http://vmd.vbi.vt.edu/toolkit). This database was also used to identify *H. arabidopsidis* orthologs of the *P. infestans* genes as described above, using both releases 6.0 and 8.3. This included searching annotated genes using TBLASTN, followed by checking the assembly when hits were not detected.

Ortholog mapping for Fig. 1 was performed using OrthoMCL (version 2.0.3; [67]) with the databases listed above as well as those for *C. elegans* (http://wormbase.org, release WS230), *D. discoideum* (http://dictybase.org), *G. lamblia* (http://giardiadb.org, v. 2.5), *H. sapiens* (http://uniprot.org, release 2011-05), *N. gruberi* (http://genome.jgi-psf.org, v. 1), *O. tauri* (http://genome.jgi-psf.org, v. 2), *S. pombe* (http://www.pombase.org, v. 12), and *T. thermophila* (http://ciliate.org, v. 2008), and *Trypanosoma brucei* (http://tritrypdb.org/, v. 4.1). *P. infestans, C. reinhardtii, and N. gruberi* proteins were used as query sequences in OrthoMCL, which was executed using an *E*-value of 10^−5^ and 30% identity as a cut-off, and an inflation parameter of 1.5. Matches with *E*-values below 10^−10^, and cases where OrthoMCL detected close sequences but could not easily differentiate co-orthologs, were double-checked using the reciprocal best hit method.

### Analysis of shared synteny and identification of gene remnants

*P. infestans* flagellar protein candidates plus the four preceding and following genes on the chromosome were searched against *H. arabidopsidis* assembly v. 6, and match coordinates were compared to assess shared synteny. This was claimed if linkage was observed between at least one upstream and one downstream gene, recognizing that some false negatives likely occurred due to gaps or assembly errors; N50 values in the *P. infestans* and *H. arabidopsidis* assemblies are 1590 and 116 kb, respectively. Similar criteria were used to compare *P. infestans* and *P. sojae*, using colinearity maps generated by Genome Project Solutions (Hercules, CA). When synteny was detected between *P. infestans* and *H. arabidopsidis*, explorations for gene remnants were performed initially by a DNA-DNA search using LALIGN [68], in which the *P. infestans* gene trimmed of introns was checked against the relevant region of *H. arabidopsidis*. If matches were not detected, then TBLASTN was used to compare the *P. infestans* protein against *H. arabidopsidis* DNA. False discovery rates were calculated as described in Table S2, and the statistical significance of each LALIGN alignment was calculated using 200 random shuffles of the *H. arabidopsidis* sequence using PRSS (http://fasta.bioch.virginia.edu).

### Growth conditions and RNA extractions

Isolate 1306 (A1 mating type, from tomato) was cultured in the dark on rye-sucrose media containing 40 units/ml of nystatin at 18°C. Nonsporulating mycelia were obtained by inoculating 25 ml of rye-sucrose broth in 100 mm plastic dishes with 10^3^ sporangia. Tissue was harvested after 72 hr, about one day before sporulation would normally begin. To induce sporulation in a semi-synchronous manner, hyphal mats were removed from broth and placed on 1.5% agar. The sample was then harvested after 20 hr, which is when new sporangia start to be observed. Sporangia were purified from cultures by adding 10 ml of water to each plate, rubbing with a bent glass rod, and passing the resulting fluid through 50 µm mesh to remove hyphal fragments. The sporangia were then pelleted by centrifugation for 5 min. To obtain zoospores, sporangia were placed in water at 10°C for 4 hr. Most sporangia released their zoospores by 90 min, and the zoospores were pelleted after an additional 60 min. RNA was prepared by grinding tissue in liquid nitrogen, followed by the use of the RNeasy Plant Mini kit (Qiagen, Valencia, CA, USA). RNA quality was assessed spectrophotometrically and by electrophoresis.

### Expression analysis

Transcript levels were calculated using Affymetrix microarrays and qRT-PCR. Expression values obtained as described below were combined, and then subjected to per-gene normalization and hierarchical clustering using GeneSpring software (Agilent Technologies, Foster City, CA USA). The microarray data are archived in NCBI GEO as series GSE9623 and GSE13580 [38], [39]; the arrays themselves are no longer available due to a limited production run. They were designed to detect 15,646 unigenes (mostly EST-based), which appear to represent about two-thirds of the 17,797 genes predicted for *P. infestans* [32]. Array data were preprocessed using Affymetrix MAS 5.1 software using two biological replicates, genes lacking “present calls” were discarded, and then the remaining data were subjected to median normalization using GeneSpring software (Agilent Technologies, Foster City, California USA).

qRT-PCR employed DNAse-treated RNA, pooled from two biological replicates, which was reverse-transcribed using oligo-dT with a first-strand synthesis kit from Invitrogen (Carlsbad, CA, USA). Amplifications employed hot-start *Taq* polymerase with primers targeted to the 3′ regions of genes, typically yielding amplicons of 150–225, using SYBR Green as a reporter. Reactions were performed in duplicate using the following conditions: one cycle of 95°C for 8 min, and 35 cycles of 95°C for 20 s, 55°C for 20 s, and 72°C for 30 s. Controls lacking reverse transcriptase and melting curves were used to test the data. Results were normalized based on primers for a constitutively expressed gene encoding ribosomal protein S3a, and expression was determined by the ΔΔCT method.

## Supporting Information

Figure S1 **Phylogenetic distribution of flagella-associated proteins present in** ***C. reinhardtii*** **but absent from** ***P. infestans*** **.**
(TIF)

Figure S2 **Alignments of** ***P. infestans*** **gene with** ***H. arabidopsidis*** **genomic DNA containing remnants using LALIGN.**
(PDF)

Table S1 **Flagellar candidates from** ***P. infestans*** **.** Indicated are the gene numbers in the Broad Institute database, common name of the locus, predicted functions, corresponding loci in *C. reinhardtii* or other flagellates, expression patterns in *P. infestans*, conservation in *O. tauri* and *H. arabidopsidis*, and whether gene remnants were detected in the latter.
(XLS)

Table S2 **Features of gene remnants detected in** ***H. arabidopsidis*** **.**
(PDF)
