# Supplementary figures and images for: Decay of Genes Encoding the Oomycete Flagellar Proteome in the Downy Mildew Hyaloperonospora arabidopsidis
Source: PLoS One. 2012 Oct 15;7(10):e47624. doi: 10.1371/journal.pone.0047624 (PMC3471859; doi:10.1371/journal.pone.0047624)

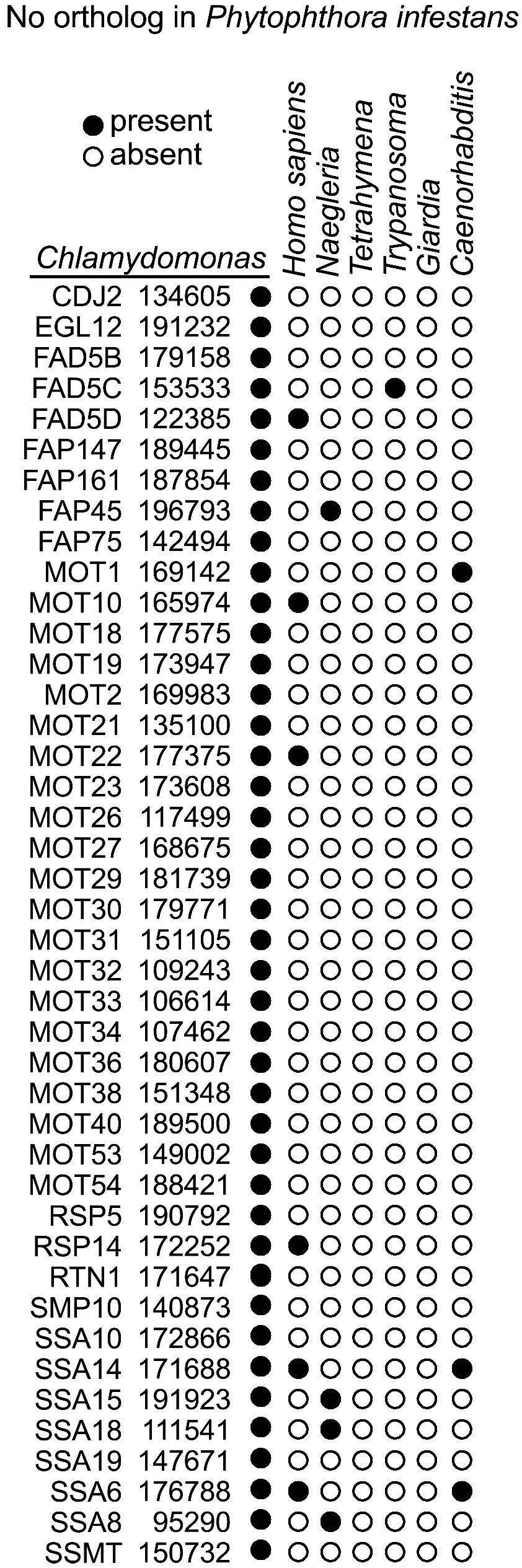

Supplement: Figure S1 — Phylogenetic distribution of flagella-associated proteins present in C. reinhardtii but absent from P. infestans . (TIF) [file pone.0047624.s001.tif]
