# Supplementary material for: Decay of Genes Encoding the Oomycete Flagellar Proteome in the Downy Mildew Hyaloperonospora arabidopsidis
Source: PLoS One. 2012 Oct 15;7(10):e47624. doi: 10.1371/journal.pone.0047624 (PMC3471859; doi:10.1371/journal.pone.0047624)
Supplement: Figure S2 — Alignments of P. infestans gene with H. arabidopsidis genomic DNA containing remnants using LALIGN. (PDF) [file pone.0047624.s002.pdf]

Fig. S2. Alignments of *P. infestans* gene with *H. arabidopsidis* genomic DNA containing remnants using LALIGN.

PITG_14226
Waterman-Eggert score: 126; 33.2 bits; E(1) < 2.3e-05
56.9% identity (56.9% similar) in 144 nt overlap

```
      1400      1410      1420      1430      1440      1450      1460      1470
PINFES GGGACTCGAGCAATACGTGCGAGTCATCTGCGCTCTGCTCGATATTCCAGTGCACAAGAACGTCTACGAGTCGCTG-CAC
       : : :: :::: :::::::    :::: :  :  : :   :  ::  : ::::    :: : :  ::::    : : :::
HYALO  GCGTCTTGAGCTATACGTGGAGCTCATGTAAGTACAGTATGC-ATGGCTGTGCTTCCGAGCATGGACGATAAACGGTCAC

       1480          1490      1500      1510      1520      1530
PINFES GTGCTCTTCACCC----TGTACCTTGAGTTCAGTAGTAACCAGCATTTTATGCCTCTATTGTAA
        :: : :: :: :    :: :::: :: :    :::: : : ::: ::  :: ::: :  ::::
HYALO  ATGTTTTTTACTCACGTTGCACCTCGATTATGATAGTGATCGGCACTTCGTGTCTCCACCGTAA
```

PITG_18065
Waterman-Eggert score: 283; 66.0 bits; E(1) < 1.8e-13
58.8% identity (58.8% similar) in 311 nt overlap (155-458:447-742)

```
           160        170       180       190       200         210       220       230
PINFES TGGATTT-GACAGCTCCAGTTGTTGCTCTCGAATGGGACACTGAAGGCAGTACAC--TCGCCATTCTACAACAAGGGAGT
       : ::: : :::  ::::  :  ::: ::::::  :: :::: :: :::::: :    ::  ::: :: :  :::   :::
HYALO  TTGATGTAGACCTCTCCTCTATTTGTTCTCGATCGGTACACGGAGGGCAGTCCTTGGTCATCATCCTGCGCCAAAACAGT

             240        250       260       270       280       290       300       310
PINFES AGTGTCGTCCCCCTA-TGGGATAACGGCACACGATCAACGTTGAGTCTTGACACGAATCTCAAGGACCCGACGTTTGCTC
       :   ::::: :  :  :: :: ::::: ::           ::  ::: : :: : ::  :    : :::  : : ::::
HYALO  AACATCGTCTCATTTGTGTGACAACGGGACC----------TGGATCTCGTCATGCATACCGGATATCCGT-GATCGCTC

              320       330       340       350       360       370       380       390
PINFES GGTGGTCCAAAGAGGGTTCGTTACTAGCAGTGGGGACTCAGAAGGGGAACCTCGTTCTCTACAGTAAAATCACTCGGAAG
         :  : : :  :::::: : : : :::   : :: :  :::::  :: : :: : :: :::  : :: :::: ::
HYALO  TATTTTTCGAT-AGGGTTTGCTGCAAGCGACGAGGTCAGAGAAGAAGA-CATCCTGCTTTACGCTCAAGTCACGCGATCA
```

```
              400        410         420       430       440       450
PINFES CTCGTTCCTGTGGTGGG-GAAAC--ACTCGCGTCGCATATTATGCGGATCTTGGAACGGAGACGACAAACT
       :: ::::   :: :: : ::  :  ::::::   :::  ::::  : :  ::: ::::: ::::::: : :
HYALO  CTAGTTCTAATGATGAGTGATGCGTACTCGCAAGGCA--TTATCTGCAGGTTGAAACGGTGACGACACAAT
```

gap.....
Waterman-Eggert score: 115; 29.0 bits; E(1) < 0.024
58.6% identity (58.6% similar) in 128 nt overlap

```
        560       570       580       590       600       610       620       630
PINFES TTGGTCGCAAGAGAGGAGACTCCATAAAAGCAGCCGAGCAGTTGATTGCAATTGCACTGTCCAAGGCCATCATTCTCTTT
       : : :::   :::   :  ::: :   ::: ::: :: :: :  : ::: :: ::: ::::::: :: : : : :  :::
HYALO  TGGTTCGTCTGAGGCAATCCTCGACGTAAGTAGCAGAACACTGAACTGCGATCGCA-TGTCCAATGC-AACTTCCCGTTT

        640       650           660        670
PINFES AACTTGCAAGACCCTG----CCAACCCCGTGGA-ACTCACCTTCCAAA
        :  :: : :  : ::    :::: :: :: :  : :: ::   ::::
HYALO  CATCTGAATGGTCATGATTCCCAATCCAGTTGTCAGTCGCCACTCAAA
```

PITG_15414
Waterman-Eggert score: 138; 34.9 bits; E(1) < 0.00011
64.3% identity (64.3% similar) in 84 nt overlap

```
       490       500       510       520       530       540        550       560
PINFES GGATGACAATTGCAGCCTATCAGACACTGTACGAGTCCAGTATCGCGTTTGGGATGCGTA-AAGCTCTCATGGCGGAGCA
       : ::: ::  ::: :: :::::::: ::::: :  :   :::::: :   : ::: :  : ::: : ::: :   :: ::
HYALO  GTATGTCACGTGCGGCATATCAGACCCTGTATGCTTGTGGTATCGAGCAAGAGATACCCAGAAGTTATCAGGAGCGAACA
       570
PINFES GAAG
        :::
HYALO  AAAG
```

PITG_13461
Waterman-Eggert score: 231; 40.3 bits; E(1) < 1.2e-06
59.2% identity (59.2% similar) in 211 nt overlap

```
              10        20        30        40        50        60        70        80
PINFES TACGACGACGTCTGCTTCTTCTTCGTGAGCTGCACCGTCTCACGTTTAAGAACGTGCAGTAAAGTCTGCGAGCTCTCGAG
       :::   :::::: :: :  :: : ::    :: :  :: :  ::     : :::::::: :: :::::  :::::::::
HYALO  TACATTGACGTCGGCAT--TCATTGTTTA-TGGATTGTGTTCCGG----GCACGTGCAGCAACGTCTGGAAGCTCTCGAT

              90       100       110       120       130       140       150        160
PINFES GATCTTCATGTAGGCAGCCTCTGTCTCCTGGATGGTACGGTCGTATTCATTACGAGCTTGTGTTTTCCTA-GCGAGCGCT
       ::::::::: : ::   : ::  :::::: : ::::: :  : : ::  ::     : ::  ::::: :  ::   : :
HYALO  GATCTTCATATCGGTGACTTCGATCTCCTAGTTGGTAAGACCATGTTTGTTGTTCACCTGCTTTTTCATCTGCTGTCTCA

       170       180         190       200
PINFES TCGTTGATCTTGCTGAGTCTGTCGG--TGAGAATGGTCAAGTCTTTCTGGA
        :  :  ::: ::::  : ::: :   :  ::: ::  :::     :::::
HYALO  GCAAT--TCTCGCTGCATTTGTAGTCATTCGAAAGGAAAAG-----CTGGA
```

PITG_4711
Waterman-Eggert score: 228; 36.1 bits; E(1) < 1.9e-05
56.7% identity (56.7% similar) in 240 nt overlap

```
             190       200       210       220       230       240       250        260
PINFES TGTGGACGCGAAATGCCAAGGAAGTGTCGATAGAATCTTGCAGCGGTCCGACGCCTACAAAATGGGTTAAACTT-TTTGA
       : ::::  ::: : :::::  : :: ::: :  ::      :: :          :  ::: ::::    : ::: ::: :
HYALO  TTTGGAACCGACAAGCCAAC-ATGTATCGCTTCAA------AGAG---------CATCAATATGGCCACAGCTTGTTTTA

              270       280       290       300       310       320       330       340
PINFES TTCAGAGTTGAAGGAGGCCGAAGGCGAAATGCAAATTGTTAACGAGAACGTCAAGCCGACTGATGCGTCCTTCATCAAAT
             ::::: :: : :::: : :: :: :::  :: : : :   :: ::::::::  : :: :: :: ::: :::
HYALO  C-----GTTGA-GGCGACCGACGACGGAACGCATGTTTTGAGCATCAAAGTCAAGCCACCGGACGCATCGTTCGTCAGCG

              350       360       370       380       390       400             410
PINFES TCAAAATCGTCTCCGGCTGGGACGACTTTGTGGTCGTGCATCGGATCAGCGTGGAAGGCTCGAGCGCTC------GAAGA
       :  ::::: :: : :::::: ::::          :::: :: : :::: ::  ::  :: ::  :: :      :::::
HYALO  T--AAATCCTCGC-GGCTGGAACGA----------GTGCTTCAGGTCAGTGTAAAACACTAGACAGCCCTGCAGTGAAGA
```

PITG_1067
Waterman-Eggert score: 144; 34.9 bits; E(1) < 0.00018
57.4% identity (57.4% similar) in 237 nt overlap

```
            890        900       910       920       930       940       950        960
PINFES GACGATAGCTA-GATTGCTCTCGTTCTCGAGGAGATCCAATGGCACCGCTGAGTGCAGTACGATCATTTGGATGC-TCAC
       :: :  ::::: :: ::  :: : :::  :: :: :::::::: :::   ::  :::: :::  :::::  :::  :  :
HYALO  GAGGTCAGCTAAGAATGG-CTAGCTCTACAGTAGTTCCAATGGTACC--AGAACGCAGCACGGCCATTTTCATGGATTGC

              970       980       990      1000      1010      1020      1030      1040
PINFES GGGGATTTCTAGCGACACGTCATAAGCTGCCAACGCCGCGTTCAGTTGGAACTTGCTGTTGACCACCAGGTCTTCCGCCA
        ::   ::: ::::::  ::            : :: :: :  ::  :::: :: ::  : :   ::::   :::  : ::
HYALO  AGGAGCTTCAAGCGAC--GT-----------ATCGTCG-GACCACATGGAA-TTTTTATCCGCCACTCAGTCGACTGCAC

                   1050      1060      1070      1080      1090       1100      1110
PINFES CTGGGAGATGTTCCTTGTCTCTGGCACCTCGCTGTAGACTCAATCGCGCGATCTTG-TCCTCAAGAGCGGCGACCTC
        :   :::   ::  ::  : :: ::: : :: :   : : ::  :::: : :::  :::  :: : ::: ::: :
HYALO  GTATAAGACAGTC--TGGTTTTGACACTT-GCAGCCAAGTTAAGAGCGCAAACTTTATCCTTGAGGGTGGCTACCCC
```

gap....
Waterman-Eggert score: 119; 29.6 bits; E(1) < 0.0071
59.8% identity (59.8% similar) in 199 nt overlap

```
         1460      1470        1480      1490      1500      1510       1520      1530
PINFES AGTGCGTACCATCGAAG--GTCAGTTTGGTCACGTAGAGGCTACGGTGGTGGCCAACACGC-AGCCTCGGTCGGCGCAAA
       :::  :: : :: ::::  : ::  ::::  ::::   ::::  : :  : :: :   ::  :::: :   : :: :::
HYALO  AGTCAGT-CAATAGAAGACGCCAAGTTGG--ACGT---GGCTCAGATCATAGCGAGTGCGGTAGCCAC---CAGCACAAC

             1540      1550      1560      1570      1580      1590      1600      1610
PINFES -CTATCAAGTTTTACGTCAAGCCTCTGTCACTGCATCACCGCGTCAACGAACTTACGGAAGCGGAAGAAGCTGAGCTGCA
        :  ::::::   :::::   : ::: :::   : :  : ::: :: ::: :: :   ::::: :::: :::::  : : :
HYALO  GCGGTCAAGTGA-ACGTCTGTCGTCTCTCAA-GAAGAAGCGC-TCGACGGACGTT--GAAGCAGAAGGAGCTGTACAGGA
```

```
                      1620      1630      1640
PINFES GAAGCCATGCAATACGCTTCAACTAAGTGGTGACTTCTC
       ::::     ::: ::  : :: ::  :: :::: : : :
HYALO  GAAGAAGGTCAAGAC--TGCAGCT--GTTGTGAATACAC
```

PITG_3488
Waterman-Eggert score: 222; 51.3 bits; E(1) < 3.5e-08
63.3% identity (63.3% similar) in 180 nt overlap

```
        780       790       800       810       820       830       840       850
PINFES ATGATCCACTACTGCCTTCACTGCCACCACCCGAACTTTGAGTAAGTAGCACGTCGCGACAGAGCTTGGCTGTTGCATTC
       : ::  : ::  :  :: ::  ::::::: : :::: ::: : ::: : :: :::::: :: ::::::::::::::  ::
HYALO  AAGACTCCCTGATATCTCCATCGCCACCATCGGAACGTTGTG-AAGAATCAAGTCGCGGCAAAGCTTGGCTGTTGCCATC

        860       870       880       890       900       910       920       930
PINFES TGATCTTTCGTGATCGTTGCATTATCGTGCAGTCCAAACACTCCCGGAGGAGCCACTAGCGGCAAACTTTCAATGAACTT
       :    :::::  ::      ::::::  : :: :: :  :: :: :::::::::    :: : :::  : :  :  :::
HYALO  T---ATTTCG--AT------ATTATCAAGAAGACCGAT-ACCCCAGGAGGAGCTTAAAGAGACAAGATGTGGA--AACC-

        940       950
PINFES AAGATACGAATCATAGTCAC
         ::: ::: :  ::: :::
HYALO  --GATTCGATTGCTAGACAC
```

PITG_2492
Waterman-Eggert score: 227; 53.3 bits; E(1) < 9.3e-10
57.7% identity (57.7% similar) in 310 nt overlap

```
           6380      6390       6400      6410      6420      6430      6440      6450
PINFES CGCGTCAATCGCTGCCATACTG-TCGCTTGGGCTCTGGTACTGCTGACTCTGTAGTCCTCGAGCCCCCGGTTGCCCTTGC
       ::::::  :::  ::   : :: :::: : :  ::: ::: :::: :   :: ::: ::  ::   : :  :::   :::
HYALO  CGCGTCCGTCGTGGC---AGTGATCGCCTAGAGTCTAGTAGTGCT-AGGTTGCAGTACTTTAGATGCAGCATGCA--TGC

            6460      6470      6480      6490      6500      6510      6520      6530
PINFES TGCCACCTTCTCTGCATCAATGAGTCTGTGAACAAATCAAATGCACCGTCGGCATACTCTGTAATGCGTGCATTTGAGCC
       ::::: :: ::: : ::   ::  :  ::    :  :  :: ::: ::: :::: :::: ::  ::   :: ::::::
HYALO  TGCCATCTCCTCGGTATAGCTGCCTAAGTAGCTAGCTTCAAAGCAGCGTCCGCACACTCCACAACACGAA-ATCTGAGCC
```

```
            6540      6550      6560      6570       6580      6590      6600      6610
PINFES GTGCAGACTGGAAACGCCCGATACGACAGCACGGCAGCAGA-TGACGCGCAACTGAGTTTTGGACAGCGCGAAGCTCGTC
        ::::: ::: :           :: : : :  :::::: : ::: :::    : ::: ::::  ::: :::: ::
HYALO  ATGCAG-CTGCAG----------CGTCTGTATAGCAGCATAGTGATGCG---TTCAGTATTGGGAAGCACGAA-CTTAAT

             6620      6630      6640      6650       6660      6670
PINFES GCTGAGTCGTAGAACGCGCGCCAAAATGCCTCAATTTCACG-AAGACGTAAGTTGTCGAAGGGGAGAATG
       :: :: : ::: ::     :: : ::    ::  : ::: : :::  : : :: :: :: ::   ::: :
HYALO  GCAGAATTGTACAAAATA-GCTAGAAGTTTTCGGTCTCATGCAAGCTGAAGGTAGTGGATGGCACGAAAG
```

PITG_4600
Waterman-Eggert score: 240; 57.8 bits; E(1) < 2.4e-11
58.5% identity (58.5% similar) in 342 nt overlap

```
              830       840       850       860       870       880       890       900
PINFES CGCATATTGGTGTCCATACAGAGTCTCCACGACCGTTTATGATAGTTGCGATCACGTCGAACAAGTAATGCCAAGTGAGT
       :::: ::::: :::::  :: : :: ::::: ::: :::  :  ::  ::::   :: :: ::::  ::::::: :::::
HYALO  CGCAGATTGGCGTCCACGCACAATCCCCACG-CCGGTTAC-ACGGTCACGATTG-GTTGA-CAAGCGATGCCAAATGAGT

              910                  920       930       940       950       960
PINFES CAAACGCAATG----------TAC-TGAAGCTCACGGAGTTGATTTCTTCTCGGTAGACGACGTCGGTCACACCTTCTTC
         :   :::::          ::  ::::  :  :: :  :: ::::  :: :  : :   ::: : :::: ::  :::
HYALO  GGATGTCAATGCGGATGTCAATAGATGAATGTTGCGTAACTGGTTTCAGCTTGTAACATAGCGTTG-TCACGCCCCCTTT

     970       980       990      1000      1010      1020      1030      1040
PINFES TTCCCAACGCATTCGTGTAGCATCCCACCAGCCAATCCGAGGAAGTTCAGCACTTCCAGCACGGATGCATGTCGTCGGAG
       :  :::: :  : ::  ::::   :: ::  :  :::   ::   ::: : :: :   ::     : :::  : :: : :
HYALO  TGTCCAAAGTCT-CGCATAGCCATCCCCCGACAGATCT--GGCTCTTC-GAACGTGACGCCGTTTTCCATCGCCTCCG-G

     1050        1060      1070      1080      1090      1100      1110      1120
PINFES CATA--CAGCACGTCCTTGGGCAATGTGAGACTAGCTTTGATCGGTAAAGCAGCAGAAACGACCATCCCATCCAAAGGAT
        :::  ::::: ::: :: : :: ::::::: ::: :: ::::      :: ::: :  : ::: ::           ::
HYALO  TATAATCAGCATGTCGTTTG-CATTGTGAGA-TAGTTTCGATC------GCTGCATATGCTACCTTCT----------AT
```

```
       1130      1140
PINFES AATGCAGTCGCTTAAGCTGTTC
       :    : ::::::::::: :::
HYALO  AGCTGACTCGCTTAAGCTATTC

gap....
Waterman-Eggert score: 164;  40.3 bits; E(1) <  4.4e-06
62.0% identity (62.0% similar) in 142 nt overlap
              180       190       200       210       220       230       240       250
PINFES GCTGCGAGTAGCTGAGCAGACGAAGAAGACGCAGCATCTTCTGCAGTGTCAGTTCAAACAAGACGTGTGAGTTGTCCATT
       :::: :: : ::::::::  :::   ::::  : ::  ::: :  : : : ::::  :    ::: ::   : :  :: :
HYALO  GCTGAGA-TGGCTGAGCAATCGAC--AGACAGATCAAGTTCCGTTGCGCCCGTTCGTAACGCACGCGTAGATGGCTCAAT

              260       270       280       290       300       310
PINFES CGATCGCGAGCATCCGGAGACGAGATCTCGTCCAGAGCTCGTCGAAGATGCACGTGTGTGTC
       :   ::: ::::: :  ::: ::: : ::  :::: ::: :   ::: ::::: ::  ::::
HYALO  CAGCCGCAAGCATTCA-AGA-GAGGTTTCACCCAGTGCTTGATCAAG-TGCACATGAATGTC
```

```
PITG_5487
Waterman-Eggert score: 456;  105.1 bits; E(1) <  1.2e-25
55.1% identity (55.1% similar) in 1023 nt overlap

            180       190       200       210         220          230       240
PINFES GAAGACATCAGTGCCGAACTTGACAAATCACCAAGGTTGTCGTGGAC--ACCACTC---AGCTGAGGCAAATAGCGTGG-
       : ::: :::  :: :    :: :::: ::: :   :  :  ::: ::  ::::::    :  :::: :::  :: :::
HYALO  GTAGATATCGCTGACTGG-TTAACAAGTCAACCCCGACGCGGTGTACGCACCACTGCCTACTTGAGTCAAGCAGTGTGAA

         250       260       270       280       290       300         310       320
PINFES -CAAATTTTGCTGCGACATCAGAACTTGTCGTGACACCGGTCGAACCGCAGCAATGCCGACT--CCTGTAGGTCCCATAT
        :: : :::::: :::::    :::    :: ::::  :::  ::: ::  : :   ::  :  :::: :: : : : :
HYALO  ACAGAATTTGCTTCGACA----AAC---ACGAGACATAGGTTAAACGGCGACGACAACGGGTGACCTGCAGTTGCAAAAA

           330       340       350       360       370       380       390       400
PINFES TTGTCGATGATACAAAGTCGCTACTATTCCATATTTTCTTTTTGCCGCCTCCACTAGTTGCAGGCTTTGGGAGATG-ATT
               ::  ::: : ::  ::  :  ::   ::  :::: :: : ::: :::: : :  :::  :   : ::  :
HYALO  --------GACGCAATGCCGTCACCGTGTCACGGTTGATTTT-GCTGACTCGACTACTCGTTGGCACTA--AAATCCACC
```

```
            410       420       430       440       450        460       470       480
PINFES GCTTCCTTTTTTGCGGCTTTTTCGACGCCCACTCAACGACCTTGAAGCCC-CTCCATCGTCATCTTTTGGTGCTGCACA-
        ::    :: :: :   : :: :  : :: :: ::  :    :      : : ::: : :: ::     :  :: : :: 
HYALO  TCTAAACTTCTTACAA-TGTTCCCGCTCCTACCCATGGTGTGTATTTATCTCACCAGCATCGTCCGAAAGATCTTCGCAG

              490       500       510       520       530       540       550       560
PINFES TTCACTTGGTTGCCACGATGCAGTCACTAGACTTGAAATATCTTCCACCGAGTCCGTCAGCTTTTTCAAGCTGCTGCTAC
       ::    ::::  : ::  ::::  : ::    ::  : :: : : ::: :::::: ::: : : ::::::::::  :: :
HYALO  TTTTGCTGGTGACTACACTGCA--CGCTGACATTCTATTAGCCTTCACTGAGTCCATCACCGTCTTCAAGCTGCCACTGC

              570       580       590       600       610       620       630       640
PINFES TGGTTGAGTGGCCACTTCCCCCTCTGTCTTCCTTGCGACTCCGCCTGCTTCCCGATCGACGTTTGTCTTTTGTAAGTGTC
       : :: ::::  ::: ::::  :::  :  ::::: :: :::: :::  :::: :: :  : ::: :         :: ::
HYALO  TTGTCGAGTAACCATTTCCAGCTCGTTGGTCCTTTCGGCTCCTCCTATTTCCTGACC--CCTTTAT---------GTCTC

              650       660       670       680       690                700       710
PINFES GAGCTCTTCTCCTCTGCCTTATACACTCCACGACTTGACATCCAGCACTT---------AGATTCATAGGACTCGGACGG
       ::   ::: ::: : :: :     :  :::: ::::::::::::::::::         :: :::  : :::: :  :: 
HYALO  GA---CTTTTCCACCGCTTCGGGTATCCCACAACTTGACATCCAGCACTTGCTCCGAGCAGCTTCGCACGACTTGATCGA

              720           730       740       750       760        770           780
PINFES CCAGCGTAGAGCTCCAGT----TGCAACGTCATCATCCGCTGTTATGGACCCTCCTG-GTTGTTG----CTTAGAAGTGG
       : :  : : : ::::::     ::     ::: :: ::  ::  ::  :::  :  : : :::    :::::: :  : 
HYALO  CAAAAGCACACCTCCAGCATAATGTTTGTTCA-CACCCCTAGTCCTGATCCCTGTTTTGCTCTTGATGACTTAGCAACGT

                 790           800       810             820       830       840
PINFES AGCAAG----AAAATC---CTTCAAAGTACG-GATGCTTTAGAC------ACTCTTCACTTGAAAATCGATCTTCAGGAC
          :::    :   ::   :::  :   : : ::::  :   ::      :::::::::::: ::: :  : ::   :  
HYALO  CTTAAGCTCTATGGTCGGGCTTGCAGTCATGCGATGAGTACAACCGCCACACTCTTCACTTGCAAAGCACTTTTTGTGT-

      850       860       870       880       890       900        910       920
PINFES ACAACCGGATGACACCTTCCAGGAAACTAATCCCCTTCTTCGAA-ACACGACCACAATACCGTCGAGTCAGAGTCTCTGG
       ::: : : ::::: :: :   :  :: : ::    : : ::::: : : :::  ::  ::  ::  ::::    :  :: 
HYALO  ACACCAGAATGACGCCGTATTGACAAGTGATAGT-TGCCTCGAACAAAGGACTGCACCACTATC--GTCAA---CATTGA
```

```
930       940       950       960       970       980       990      1000
PINFES GGCTTTTACTTCGGGAATCTTGAGTCCGGAGAAGCGAGGATTCATTGCAAATAGCTCCGTGTGTCGTTTCTGTAGCGGAC
        ::        : : ::: : : ::: :: ::: :    ::    :: ::: ::: ::      :::: ::: :: :: :
HYALO  TGC-------ACCGAAAT-TCGCGTCGGG-GAACC----ATCGGCTGGAAA-AGCACCA----GCGTTCCTGGAGTGGGC

      1010      1020      1030      1040      1050      1060       1070      1080
PINFES CCAGCATTTTCTGGATCAAGTACAGTTGATCAATTTCGCTTTCCCCCGGGAACATCGGCT-GCCCCTCAATAAGC---TC
          :::: : ::: : ::: ::::: :::::: ::::::: :   :    ::  :: : : :  : :: : : ::   ::
HYALO  TG-GCATCTCCTGTACCAAATACAGCTGATCAGTTTCGCTCTTTTCA--AAAGCTCAGATTGATCTTCGACATGCAACTC

          1090      1100         1110      1120      1130      1140
PINFES ACCCATAATGCACCCGATTG---CCCAGACATCCACAGATTTAGAGTACGTGGTATCCCCAAG
        ::   : :: ::  :  :    ::::   ::::::: :: : ::::   :: ::::  ::::
HYALO  TCCTGGACTGAACATGTGTCACACCCACGTATCCACA-ATCTTGAGTTGCTGATATCGACAAG
```

PITG_6195
Waterman-Eggert score: 202; 48.6 bits; E(1) < 1.3e-08
60.0% identity (60.0% similar) in 170 nt overlap

```
            1160      1170      1180      1190      1200       1210      1220      1230
PINFES CTACATGGCGTGTTTATTCGACAAGGAGAGCATGACAAAAAATGATCTACGCG-ATAATAACAATGCCACCACGTCGCGT
       :: :: : :  :::   :   : : : :: :::  :  :  : ::::: : :: : :: :::: ::  :: :: :::: :
HYALO  CTGCACGACAGGTTACCTGTCCGACGTGACCAT--CTGACTACGATCTCCACGCAAAACAACATTGTAACAACTTCGCAT

             1240      1250      1260      1270      1280       1290      1300
PINFES CAAGGATCAAGTCGTGTGGCTCTGTATCGAGCTACTGGACTCACGTACGTGT-ATACCATTGAATGTAACTACAACGAAG
       : :: ::  ::: :    ::  ::::::  ::::: : ::: : ::: : :   : :   :::: ::::  : :: : :::
HYALO  CGAGAATGTAGTTGCACAGCATTGTATCATGCTACAGAACTGATGTATGAGGCACAAACTTGAGTGTACTTGCAGCAAAG

       1310
PINFES GTCGGCGTAA
        :   :::::
HYALO  ATTTCCGTAA
```

gap....
Waterman-Eggert score: 105; 26.8 bits; E(1) < 0.045
54.5% identity (54.5% similar) in 222 nt overlap

```
              240       250       260       270        280       290       300
PINFES CTGGTTCTACTTTGAGGTAGAACGGACTCTTAAGGCCGCCAAA-CAACCGCAAGATCTGCACGTGACATTGGTGAATCTG
       :::: ::::::      ::    :::::: ::  ::  : ::: :::  : : :     ::: :::  ::  :::: ::
HYALO  CTGGATCTACTCAACCTTATTGTGGACTCGTACTGCTTCAAAAACAA--GAACGTGGATCACATGAACTTTTTGAAGCTC

     310       320         330       340              350       360       370       380
PINFES AATCCACAGCG--AGGACTATTCAAAAATGGCT-----ACAC--GATCATGCACAGCTCGGACGAGGAACGATGGGACCG
       :: :   ::::  ::  : :: ::   :  ::      :: :  ::: ::  ::   : :::: : :   : ::::: :
HYALO  AAACTGAAGCGCAAGTTCGATGCAGCCACCGCGCGCCAACGCCGGATGATCGACTTATTGGACAATGTGTGGTGGGAGCA

                   390       400       410       420       430
PINFES G----TTGCCTTC-ACCGCTGATGAAAATTCGACTGTCGCTCAGCTACAAATTCAAATTTGC
       :    :::  ::: ::: ::  :  : :::: :   ::::::     : ::   : :: :::
HYALO  GCTCTTTGGTTTCCACCTCTTTTTCAGATTCAA---TCGCTCCATGTCGAAGGGATATGTGC
```

PITG_16089
Waterman-Eggert score: 295; 68.3 bits; E(1) < 9.9e-15
58.9% identity (58.9% similar) in 338 nt overlap

```
               10        20        30        40        50        60              70
PINFES TCAGCCAGTTGAGTCCATGAGGTCGAGCAGGTCGTCGCCTCCTCCTTTACCTTCGCGTTTGGTCTCGTC------CATCT
       : :::  :::::::::: ::     : :: :: : :  :  : :   : : : :: :: :::   :::       :::::
HYALO  TGAGCGCGTTGAGTCCACGAA----ACCAAGTAGACAACGGCGCTACTGCGTCCGTGTGTGGCGCCGTTGTCAGTCATCT

           80        90       100       110       120       130       140           150
PINFES TGAAGTCCTGCAGTATCGCATCAGTCGAAGTCTTGCGGTCACTCGCAGCGTCAATCCAAATCGTAGGAA----CTTCTTT
       ::::: ::: : ::::::: :  ::  :::: :::: ::: :::::: :::: ::: ::   : :: ::    : : ::
HYALO  TGAAGCCCTACTGTATCGCGTTGGTGAAAGT-TTGCAGTCGCTCGCA-CGTCGATCGAAT--GGAGAAATCTACCTGTTG
```

```
               160       170       180        190       200       210       220
PINFES ATCGTCGTCGTCATCTCCACCAGATCCAGGCTTCG-TGGGGTCGTC-TGGGAACAGGTTGATACGCAACGCCTTGTTGTC
        : : : :: :  ::::     :: :: :  :::: : : ::   : ::::  :: :: : :  ::::::: : ::::::
HYALO  CTTGCCCTCTTTGTCTCTTTG-GACCCCGTATTCGGTCGAGTTACCATGGG--CAAGTCGGTT-GCAACGCTTCGTTGTC

       230       240       250       260       270       280       290       300
PINFES CCCTTCGACATCCTTCGCGGACGAAGATTTCCCCACAAGATCAGCGAGCAGGTTGAGTCCATCCGCTGCGGTGGCTTTAA
        :    :::::  :: :  :: :  :: :  :   :  : :  : :::::: :::::::     :: :: :  ::: :
HYALO  TCTAGTGACATATTTTGTAGATGCTGACTGGCTTGCGGGTTATGTGAGCAGATTGAGTCTGGTGGCCGCTGCAGCTCTGT

       310         320
PINFES AAGTCTTTT--GGTGCTT
         ::: ::   : :::::
HYALO  TGGTCGTTGACGTTGCTT

gap....
Waterman-Eggert score: 177;  42.5 bits; E(1) <  5.7e-07
62.0% identity (62.0% similar) in 150 nt overlap

               500       510       520       530       540       550       560
PINFES GGTCCTTCGGGCGCTTCAACAGATCCCTCCGCACTGGCAATAGCGAACGTTTTTTCTTCCTCAACACGGTTC-GACGCGT
       :: :::: :   :::::::: :: :::: :  :::: ::::   :: :   :: ::::  :  :: : :  : :: ::::
HYALO  GGCCCTTTGAT-GCTTCAACGGA-CCCTTCAGACTGACAATTA-GACC---TTGTCTTTATTGACCCTGAGCTGATGCGT

      570       580       590       600       610       620       630
PINFES CAAAGTAGCGAATTCTACCCGGCACTTTGCCGCCTGTAGCCGTGAAGCCTTCGTAGTGAATGGCCAGCGT
       :: :   :: :    :  : :::   :: : ::  :: :::::::::::::::: ::  : :  ::  ::
HYALO  CAGAACTGCTACGGTTTTCTGGCCACTTACTGCTAGTCGCCGTGAAGCCTTCGTTGTTGACGATCACTGT
```

```
PITG_12109
Waterman-Eggert score: 195;  46.7 bits; E(1) <  1.9e-08
58.8% identity (58.8% similar) in 216 nt overlap

              450       460       470       480       490              500       510
PINFES CGCAACCGCCTGACAACAAGCGCAGACTTACTAGAGCTCTCTCAATGTCCGC-------TGCTGAACAACATCGATATCA
       : ::: : ::: :: : :::: :   ::: : ::::::  : :: : ::::        : :: ::::::: : : ::::
HYALO  CACAATC-CCTTACTAAAAGCTCG--CTTGCAAGAGCTGGCACATTTTCCGGACTTGCATACTCAACAACAACAACATCA
```

```
          520        530       540       550       560       570       580       590
PINFES GCCACAACCTAA-TCGAGGACCCAGATATATTGAACGTGCTCAAAGCGGTGCCAATGCTCAAGGCGCTACGAATCACAGG
       :::     : :: :: : : : : :   : :  : :::::::::     : ::: :    :: :: :: :  ::::  ::
HYALO  GCCTGCGTCAAAGTCAAAGTCACGG---TGTCAAGCGTGCTCAAGT--TTTCCATTAAAAAAAGCACTGCCGATCAGCGG

           600       610       620       630       640
PINFES CAACCCAGTGGTCTCCAGCACGCGCTCGTTTCGAAAGACGTACATTGCAGCTCTAC
       :::   :::::::: ::  :  : : : :: :   : :  ::  :::::::: : :
HYALO  CAATATAGTGGTCTGCAA-AGACACCCTTTCCCCGATATATAGGTTGCAGCTATTC
```

PITG_14588
Waterman-Eggert score: 159; 38.2 bits; E(1) < 5.7e-06
63.6% identity (63.6% similar) in 99 nt overlap

```
        390       400       410       420       430       440       450       460
PINFES AGGCTGCTCGATACGGGAGGTGCCGCTGTTTCGGCCAATGTCTTCGTCACGAATGCAGCAGTTTATAGCGTGGCTAACGG
       :::: :: : :: :   : : :: :::::::::    : :: ::: :::   :: ::::::::: :  :  :::   :::
HYALO  AGGC-GCACAATGCACAAAGCGCTGCTGTTTCGTGGGACGTTTTCATCATAGATACAGCAGTTTGTGACCGGGCCTCCGG

        470       480
PINFES GATCACCGCCCGAGTATTT
       : : : :::: :  ::: :
HYALO  GCTAATCGCCTGGATATGT
```

PITG_8015
Waterman-Eggert score: 278; 66.6 bits; E(1) < 3e-14
55.6% identity (55.6% similar) in 478 nt overlap

```
            80        90       100       110       120         130       140       150
PINFES TGCTGCCGCGCACGATCGATATTTGGTTCAGGGAATTGGCTGGATGACTCTT--CTGCGTCTTTATCGTCTTGCTCGCCT
       :: :  :: : ::::::::: :: :: ::: :   : :  :    :::: ::  :   ::   : ::::: :: :::: :
HYALO  TGTTTGCGGGTACGATCGATGTTCGGATCATGTGTTGGATTATGGGACTATTTCCGAGGTTGGTGTCGTCGTGTTCGC-T
```

```
             160            170       180       190        200       210       220
PINFES CCTTCCTTCT-----GCGTAAGTAGGCTTTCAGCTATATCAACTGATGGAGA-ATAGTATTCGCTTGAGATTTTAATCCA
       :  :  ::::     : :: :: : : :::::: :  ::   : ::  :::: :::::   : :   :  ::    : :
HYALO  CAATTTTTCTTACCCGAGTTAGCAAGTTTTCAGTTGCATTG-CCGACTGAGACATAGTCCACTCACAAACTTGGTTTACC

         230       240         250       260        270       280       290       300
PINFES TTGCCGTGCTTTCTC-TTCGTGCT-GATCTTTCCCGAATCGGTC-TTGGCGCCACGCAGCGCTCTTTGGAACACCGATAT
        :    : ::::::: :: ::::: : ::: : : :   :::::  ::   :::: :: : ::  ::: : :  :  :
HYALO  GTCGTATACTTTCTCCTTTGTGCTAGGTCTGTACGG---CGGTAATTCAGGCCAAGCTGAGCGTTTTAGCATGCGCAAGA

            310           320           330         340       350       360       370
PINFES CCTCGAAAAGA----TCAGCTCC----AGCAGGTACGAAA--AGCGGTTTGGCTGGGTCGACTTCTTTACTGGCACCTCG
       :::  : :::     :: : ::     : : : : : :::  ::: : ::  :     :::: ::: : :     :  ::
HYALO  CCTTTACAAGCGGGCTCGGGTCTGCAAATCTGATGCAAAAGTAGCAGATTTACATAACCGACCTCTATGCAAATGCTACG

            380       390       400       410       420        430       440       450
PINFES AGCCATAGCTCGGAATGCAAAGCCTTTCATGACGGACCGAAATTGCGTGACA-AGGTTCTTGTCCTTCATACTGGTAAAC
       :::  : :: :::: : : :: :  : : : :::  ::  ::::  ::: :  : : :  :::::::::::  : :  :
HYALO  AGCGGTTGCACGGATTACGAATCAGTCCGTTACG--CCACAATTT-GTGCCGTAAGATGATGTCCTTCATATGGATGGAT

             460       470       480       490       500       510       520
PINFES AAGACGCTGGCCCCGTACATATGTGAGAGATACCTTACTGCCTGAGTCACTCCTTTGCGCTTGATTGAGTCCTCGTCT
        : ::: :::: :  : ::  :::::::  : :  ::: :: :  : ::  :   :::    : :: :: : : ::::
HYALO  TATACGTTGGCGCGATGCAGCTGTGAGA--TGCTCTACGGC-TCGGACAAACAGATGCTGCAGTTTTAGGCTTGGTCT

gap...
Waterman-Eggert score: 167;  41.3 bits; E(1) <  1.3e-06
63.7% identity (63.7% similar) in 124 nt overlap

        920       930       940       950       960       970       980       990
PINFES GCTGTGGTTGGTTTGATCTCCTCGGCTTTGGACGAATTCCGGAAATTGGCTGTCAGAGAGGTTTTACCTGATCCGCGAGG
       :: ::  : :: ::: :::  :: ::: ::  : :::::: :::  :: :  : :::: ::::: :: ::: :: :   :
HYALO  GCAGTTTTAGGCTTGGTCTGTTCAGCTGTGACCAAATTCCTGAACCTG-CAATAAGAGCGGTTT-ACTTGAGCCACTTCG
```

```
       1000      1010        1020      1030
PINFES ACCGACAATCAGCGTGAAGG--TGTCACGTTCCTCCGGTGCTGG
       ::  ::: ::   :  :: :  :: : : :::::  :::::: :
HYALO  ACTAACA-TCTTAGGCAATGATTGCCTCATTCCTTTGGTGCTTG
```

PITG_3878

Waterman-Eggert score: 283; 65.3 bits; E(1) < 1.7e-13

59.0% identity (59.0% similar) in 290 nt overlap

```
     440       450       460       470       480       490       500       510
PINFES GGCTCCGGTAGATTATGATCCCATAGCTTCACGGTCCAATCGGCGCTACAAGACAGGAATGCTCGCTCGTGGAAGGGATT
       :: ::  : : :::::::  :::: :::::::: : ::: :: :::  ::: ::    ::    :::::::::::  ::
HYALO  GGATCGAGCACATTATGACTCCATGGCTTCACAG-CCAGTCAGCGTCACA-GATTATAAAATCCGCTCGTGGAATAGA--

     520       530       540       550       560       570       580       590
PINFES CCAGCGCACAGCATACACTGCCATGTGGTGCCCTGCATACGTCTCGAGATACTGTCCAGAGTACGCTTTCGAGCACTTGT
       :::: :: :  ::: :::::  ::: : ::  ::::    : ::    : : :    :::: : ::::: :: ::: :
HYALO  CCAGTGCGCGACATGCACTGTTATGCGATGATCTGCTGCTGGCTTACAACATTCCAAAGAGCATGCTTTTGAACACATCC

     600       610       620       630       640       650       660       670
PINFES GAAGCTTGCCTTCCTCCGTACCGACAATGAAAAGGTGCTCACTGAAGCGGTTAAAGTCAAAACAACATCCACCTGCAAGA
          : ::  :::: : ::: :      ::::    ::::  :: :: :  :  :::  :   :: ::  ::  ::
HYALO  CCGGTTTCTCTTCTTTCGTGC------TGAA---CTGCTTGCTCAA-CAAT--AAGCGATCCCAGCAGTCAATTGT----

     680       690       700       710       720
PINFES CCAGATAAGGACGCTTCTTCAGGATCGTCCTTGGCCGCTGCGACTAGCTT
          ::::: :: ::  ::::: ::::  :  : : ::::::::  :  ::::
HYALO  ---GATAAAGATGCCACTTCATGATCACTCCTAGCCGCTGCCGCATGCTT
```

PITG_7793

Waterman-Eggert score: 170; 43.9 bits; E(1) < 8.8e-08

58.2% identity (58.2% similar) in 196 nt overlap

```
             150       160       170       180         190       200         210
PINFES GAGCTCGAGGGCAAAGAGTTTAACGAGAGCGACTGCAAGGGC--TGGAGCATCGCTATCGCCGACGCC--GTCAAAGCTC
       ::: ::  ::     :::::: : :  :: ::  :::: ::   :: :::   :: :  :: :::::   :: :  :::
HYALO  GAGGTCCTGGCGGTGGAGTTTGAAGGCAGTGAGAGCAAAGGACTTGAAGCGGTGCGACTGCAGACGCTCGGTTAGGGCT-
```

```
       -220       230       240       250       260       270       280       290
PINFES GCATCCACACTGATTGTAGCTTCCCACGCTACAAGATCGTCGTACAGACGTTCATCGGCCAGCAGAGACTGCAGGACGTC
       : :: :: :  ::     : :::::::   : ::: : ::: : : ::: :  :    ::::: :: :: :    :::::
HYALO  GAATTCATATAGAGCCAGGGTTCCCACAACAGAAGGTTGTCCTGCGGACATCTACTAACCAGCTGATACCGTCT-ACGTC

       300       310       320       330
PINFES CGTATCACGTCTCGCTGTTTGTGGGACGACGATCAT
        ::::  : ::::     ::::  ::  ::: ::::
HYALO  -GTATACCATCTCATCACTTGTACGATCACG-TCAT
```

PITG_20902
Waterman-Eggert score: 261; 56.0 bits; E(3501) < 3.5e-08
56.5% identity (56.5% similar) in 462 nt overlap

```
      170       180       190       200       210       220       230         24
PINFES GAGGTGTCTGCGTTACTGAACGAGAAAGGAATCAAAATGACTCGTGAGGAGATCGAGGACGAG--CTTCGTATAATCAAC
       :: : :: :::: : :::::: ::    ::: : :  :: : :::::   ::: :: ::::::  :: :: :    :  :
HYALO  GACGAGTTTGCGCTGCTGAACAAGTC--GAAACTATGTGCCACGTGAACCGATTGAAGACGAGGTCTGCGAACGGGCG-C

        250       260       270       280       290       300       310       320
PINFES CAGAGTCTAGAGAAAGCCGTAGTCAATCGCTCTGTGGTGCAGAATAGCAACGCCCACAGTCGTGTGATCAAGAACGAAGA
       :::  :: : :::: ::  :    :: :     :  :::::::: :: : :  :::::: ::  :: :::  ::::::: 
HYALO  CAGC-TCAACAGAATGC--TTCAAAAACCAAAAGCAGTGCAGAACAGTAGCAGCCACAGCCGGATGGTCATAAACGAAG-

        330       340       350       360       370       380       390       400
PINFES CCTATACGAGGCTATCAAGGCTCTGGGCAAAGTATGCTCGAAGAAGGAGGTCAGTGATATGATCTGGGAGGCCGACGAGA
         :::   : : : :::     :  :::   :: :: ::::: :   :: :::: ::: ::::    ::::  ::::  :
HYALO  --TATTGCAAGGTGTCAC----CCCGGC---GTTTG-TCGAA-ATCAAGATCAGCGATGTGATTGAAGAGGTAGACGGTA

        410       420       430       440       450       460       470       480
PINFES ATCTGGACGGTGTAATCGACTGGGAGGAACTGCGCGCCATGTTCAATCGCAACCTGCTGGACAAGACGGAGCTGGAGCCA
         ::::     :::        ::: :: ::::: : : ::::: :  : :   :::: ::: : ::  :  ::: ::: 
HYALO  CCCTGG-----GTA--------GGAAGAGCTGCGTGTCGTGTTCGAATGTACGATGCTCGACCA-ACCTACTTGGCGCCG
```

```
        490            500         510        520       530       540       550
PINFES GCCAATCT-----CTTCAAC--GTCGTCCAGTT-CATGACGTACGACAAGAAAAACTGTGGAGTCATCACTGCAGACGAC
         :: ::      ::: : :  :   :: :    ::  : ::::  ::    ::::: : :::::    ::: :: :  :
HYALO  TACATTCATCGCGCTTAAGCCAGCACTCAAACCACAGCATGTACTCCACCTGAAACTTTAGAGTC----CTGGAGGCATC

      560       570       580       590        600       610
PINFES ACTATGGCCATCCTCTTTGCGCGCTACGGCCAGT-CGCAGCTCGAGATGCGCATGAAGCAGC
          ::::  ::: ::::   ::  :: :  :::: :: :  :  :  :  ::: :: ::
HYALO  GACATGGT-ATCATCTT---GCATTATGATCAGTACGTACGTATACGTATGTGTGACGCTGC
```

PITG_1312
Waterman-Eggert score: 216; 28.2 bits; E(1) < 0.018
54.7% identity (54.7% similar) in 384 nt overlap

```
           940       950       960       970        980       990       1000      1010
PINFES CTAGATCTCCGACTAAATCGCCTCACCGACGATGGGGGGCGCGTGT-TGCTGGAAGGCCTGGTGGAT-CATACGTCGCTG
       :: : :: : ::   ::::::: : :  :: :: ::: ::    ::: : ::: : ::: : ::: :  :  :: :: ::
HYALO  CTTGGTCGCTGATGGAATCGCGTAAGGGATGAAGGGAGGTAA-TGTCTCCTGTACGGCTTTGTGAAGACCCAC-TCACTA

            1020      1030       1040      1050      1060      1070      1080      1090
PINFES ACCAGTCTGAACTTGAGCAGCAACATG-TTGGGCACGGAAAGTGCTGAGGCGTTGGCTGAAATCCTCAGTGATTCACCGA
       :   ::::  : :::::  :: ::::: ::  :  :  :::: :   :::::: :::::::           :: ::   :
HYALO  ATTGGTCTTCAATTGAGTCGCGACATGCTTTGTGA--GAAACTATCGAGGCGCTGGCTGAT----------ATGCAATAA

             1100      1110      1120      1130      1140         1150      1160
PINFES CGGCACTCCGCTCTGTAGATCTGAGCAGTAACGCGATGAGTGAAAGCGAT---GGAGACGTGTTGCTGCAGGGTTTGCAG
       :::: ::  ::::  :  :   :   : ::::          :::::: :   : : :   :: ::: ::  :     ::
HYALO  CGGCGCTTGGCTCAATTCAATGGCATATTAACTTATCTCCCTAAAGCGGTCTCGTAAAAAAGTCGCTACAAG-----CA-

      1170        1180      1190       1200      1210      1220      1230      1240
PINFES CAGAATAA--CACGGTCATAGCGCTAGATCTGC-GCCAAAACACGCAGATCCCGGCTGATGCTGATTGCCTCGCTAAAAT
       : :::::   :::  :::::    :: : : ::::  ::   ::  ::   :::  : ::     :::  :: ::
HYALO  CGGAATATGTCACCATCATA----TACAGCTACCGCCATCAC---CATGTC---GCTCCTTCTTCGCTCCTTTCTTAA--
```

```
         1250       1260      1270          1280      1290
PINFES CGCCCAGAAAACCAG-ACAGAATGAGGTGAATA----TCAAGAACCCTTCCT----CCACAGCA
        : :  :: : :::: :: : ::: : : :  :    :  : ::: ::  ::    ::::::::
HYALO  TGGCGTGAGA-CCAGTACCGCATGCGTTAAGGAAACGTACACAACGCTATCTACAGCCACAGCA
```

PITG_0627
Waterman-Eggert score: 178; 27.3 bits; E(1) < 0.012
54.4% identity (54.4% similar) in 250 nt overlap

```
          100       110       120       130       140       150          160
PINFES GCTTTTGGATCAACGTTGACGACTTTCCTGTCGCGTTTGTTTTTAAACAGTAGCCCAGTTTCAC---TGTCA-----GCG
       :: :: : ::  :  ::::::  : :    : :: : :::  :::: :  :::    :: :: :   :: ::     ::
HYALO  GCCTTCGTATTCAAATTGACGCTTATTTCATTGCATATGTACTTAAGCCCTAGAATCGTCTCGCAAATGACATAAAAGCA

           170       180       190        200       210       220       230       240
PINFES ATCACTTGGAAATTTGGTGTGGAGTTAGA-AGTGGAAGTCTGAGTCTCCCAATTGTAGTAGCTCAGATCCGAGGATTTCG
       :::   :: ::      :::  :: ::   ::: :::  :   :: :             :::    ::::   :::::
HYALO  ATCG--TGCAA------TGTCAAGCTAACGAGTAGAAAGC---GTTT-------------GCT----TCCG---ATTTCA

         250       260       270       280       290       300       310       320
PINFES GTAGCAGCTTCTTCTTGCGCTCGAAATATGGCTCGAACGGTTCATTTTTCAGACGCAGCGCGTAGTCGATATAGCCGCTA
       : ::::: :: : : :: :: :::: ::  : :::::: :: : : :: : ::: :::: : :  :    ::::: ::   :
HYALO  GCAGCAGTTTTTCCATGTGCGCGAACTACAGTTCGAACAGT-CGTTATGCAGGCGCAACACAAAACTCATATATCCATAA

           330
PINFES ATCT--CTTG
       :: :  ::::
HYALO  ATTTAACTTG
```

PITG_3419
Waterman-Eggert score: 396; 39.3 bits; E(1) < 2.4e-05
51.9% identity (51.9% similar) in 1446 nt overlap

```
             20        30        40        50              60        70        80
PINFES CTGCTACGCTGGCTCAACCACGAGCTGGAGCTGTCTTCTCGTGT------GACGGACGTGGAGTCGGACTTTTCCAGCGG
       ::::: ::::::::::::::: ::::::   : :::  : ::         ::   :  ::: :  :::: :: ::::: :
HYALO  CTGCTGCGCTGGCTCAACCATGAGCTGCGACCGTCGACGCGCACACCACAGAACCAATTGGGGGTGGACATTACCAGCAG
```

```
           90       100       110       120       130          140       150       160
PINFES CT--ACTTGCTTGGAGAGATCCTGCACCGTCTCAACCACCAGCACAACTTCGC---CGACTTCATGCGAAGCTCCAGCGC
        :  ::    : ::   : : ::: :::: :: :::          :: ::::   : :  : :  :: ::::::  :::
HYALO  TTCCACCGATTCGGCTCGGTTCTGTACCGGCTTAAC----------ACGTCGCGCGCCATATTACACGCAGCTCCTTCGC

             170          180        190       200       210       220       230
PINFES CGACGCCAAAGTCGT---TAACTTCT-GCTTGCTGGAGCCGGCGCTGCACAAACTTAACGTTAAGTTTGACGCCAATGTG
       : :::: ::  :: :   ::   ::: :   : :::::::   :   : : : :: :::  ::   : : : ::::::
HYALO  CAACGCTAAGATCCTCCCTACTCTCTCGACCGTTGGAGCCCATGAA-CGCGATCTCAACCGTACACTCGGCACCAATGCA

       240        250       260       270       280       290       300       310
PINFES GCC-GTCGCTATCATGAACGAGAAGAAAGATGCCGCCGCCAACCTGCTCTACCAAATCAAGATGGCCGCAGCCCGAGTCG
       ::: ::  : :::  :::: ::::::  : :::   ::   ::   ::: : :  ::: : ::          :   ::
HYALO  GCCAGTGCCAATCGAGAAC-AGAAGACCGTTGCGATCG---ACGCACTCGATCGGATCGATAT----------CCTTTCT

        320       330       340       350        360       370               380
PINFES GGAGAGCTCCAAATGTTTCGACCAAATCGCTGGAGCGGACTG-GTATCATTCCTT------TGCACAA--CAGACCCGTG
       :  :   : :  : :  :::  : : : : : ::  : : ::  :  ::  : : :::       :: :: :  :   : ::::
HYALO  GTTGTTTTACTGA-GCCTCGCTCCAGT-GCCGAAACGTCCTCTGTGCCTTTTCTTCTGTTCTGAACCATTCGATCACGTG

       390       400       410       420        430       440       450       460
PINFES AAACTTGCGAAGCCAATCTACGACTCAGAAAAGCACAAGC-TGTTTGAGCACTCGATTCGTCGCCATGTGCGGTCGAGCA
          ::: :     :: : :     :::::: : :  : :: :  :::: ::: ::          ::: :: : : ::  :
HYALO  ---CTTCCTT---CATTGTTA---TCAGAAGAACGTATGCGTCGTTGA-CACCCGCA-------CATTTGAG-TAGAT-A

        470       480       490       500        510       520         530       540
PINFES CTTCGTTGCAGCAAGAAAAGTGGACGAAGAAACGAGAGGCT-TATCTGGCGAGCATGGCG--GAGCAGAGAGAGATC---
       ::  ::::::    :   : : ::  ::::  :: : :::  ::: :   :  : :::: ::: : : : : :::
HYALO  CT--GTTGCATAGTGTCCATTCGA--AAGA--CGTGTGGCACTATATCAT-ACAAAGGCGAAGAGAATACATAAATCGAA

                550          560       570        580        590       600       610
PINFES --CTGGACAATA---CAAAAGCAGAGAGACTGCACCGCGCA-TACATCCATA-GCAGCTATATTAAAGAAGCTTTCGAAG
         :  :::  :    :  :    :: :::: :  : :   : :: :::::   : : ::  ::  :   :    : ::::
HYALO  CTCGTGACCGTTTCTCTCACTACGAAAGACGGATCAGGAAAATAGATCCACGCGAATCTGAATGCATACACAGCTGGAAG
```

```
               620       630       640          650       660         670       680
PINFES AG----ACTGATTCGCCGGCATGGAGACTGGCACTG---CAGAAAAAGAATGCATGCGA--GCAGCGACGGGTTGCATTC
       ::    :::: :       : ::::  :  :: ::    ::: : :: : :   ::: : : : : ::   ::  ::
HYALO  AGCGCTACTGCT-------CGTGGAAGCAAGCGCTATGTCAGGACAATAGT---TGCTACTGTACCAACTCTTTTTATCA

          690       700         710                    720       730       740
PINFES TTACAGCAGCTGATGAAG--AAGCGCGAAG-------------AAGCGGATGAGAATCTTACCTTCAGTCTTCGTCGTAA
       : :: :::: :::   ::  ::::  :: :             :::::: :::: :: : : :  :  ::  ::     :
HYALO  TGAC-GCAGATGACACAGCAAAGCAAGATGCACAATTCTGTCGAAGCGGCTGAGCATTTGATCGACCATC--CG-----A

     750       760       770       780           790         800       810       820
PINFES AGTTTGCAATGAGCTAGATGATTTCGATGTACATCGATC----AAAGGCTG--GAATGGCGGGCAGCAAGGTTTCTTCAC
        ::: ::::  : ::: : :    :  :::: :::: :      ::::  ::  :::      : : : :: ::  :  : :
HYALO  TGTTAGCAAC-ACCTACAAGGG--CATTGTATATCGTTGCAAGAAAGTATGAAGAAACTGAGTCTGGAATGTCCCGCC-C

           830        840       850       860       870       880        890
PINFES GGAAATCGGTTG-GATATGGTCTACGCTCACTTACAACAGCACTGAATGTATCGTCCGACAAGCA-GAAGT---ATTCAA
       :     : :: : :::   : :: ::   :: :: ::::   : : : : : :::  :    ::: :: ::   : ::::
HYALO  GTGGTCCTGTCGTGATTGTGGCTGCGGGGACATAGAACAAGTCCGGACGCA-CGTGTG---CGCACGACGTCAAACTCAA

         900        910        920          930       940         950       960
PINFES A---TGC-TCACTTGGCGGGATCAG-CGATGAACGA---AGCGGAGCTTTATCAAGCA--AACATGTTGGAAATGGAAGA
       :   ::: : ::: :   : ::::: :::  : : :   :  :::   ::: : :::   :::::: : ::  :
HYALO  ACGATGCGTTACTGG--TGAATCAGACGAGAAGCAACATATTGGAAAGTTA-CGAGCTGGAACATGCTTGATTTATTTTT

      970         980       990      1000       1010      1020      1030      1040
PINFES CG--CATCGGGCGTCATCAAGCAACAAAGGGCTCTACGTGGAA-GACGCAAGGACAAATTGTCTCGCAGAAGGAAGCGGT
       ::  :: :   : ::::: :::  : :     :: :::: ::  :: ::    :::  ::: :::: ::   :  : : :
HYALO  CGTCCAGCTCCCTTCATCGAGCCGCGATCCCTTCGACGTCGATCGATGCTGAAACACTTTG-CTCGAAGC--GCTGGGTT

        1050      1060       1070          1080       1090      1100      1110
PINFES TTGTTCAAGATTGCGTGAATACG-CACT----CTCTAATAGGAACC-GCTCGTGTGGCGAGTATTTTGGAAAATCTGGTG
       ::: :: ::      :  :: : : :: :    ::  :: :::::   ::: :        :::::    :: : : :
HYALO  TTGCTCGAG-----GATAAGATGACAATTGGGCTACAACAGGAAAAAGCTTG-----ATAGTAT----GAGACTTTCCCT
```

```
    1120      1130      1140       1150           1160       1170      1180
PINFES CTGCGTGACACCAACAGCGAGAACGATATA-CAGCA--TGAAA---TGGAC-CGCATTCTCGTGTACAA---AGAAATAG
       : :  ::      ::::    :: :  ::  ::: :  ::: :   ::::  : :  :: : ::: :::   ::::  ::
HYALO  CCGGTTGT-----ACAGTCGTAATGTGATTGCAGTAATTGAGAAGATGGAGGCACGATCACATGTGCAAGACAGAAGCAG

    1190      1200             1210      1220      1230      1240      1250      1260
PINFES CTCGGGAGAACAGGTCAATG-------CGAAACCAAGAGTATTCGAATCAGCAGGAGGTGGACTCCATGACAGCGATGGA
         :::       ::::::::       :: :::: : ::    :::  : :  ::   ::: :  : :: ::  :::  
HYALO  --CGGC------GGTCAATGACTTGCTCGCAACCCAAAG-----GAACGACC--GAAAGGGAATGTA-GAGAGACATGTC

            1270      1280      1290           1300       1310      1320      1330
PINFES TCGAG-ATACTAGCTGCTACCACAATCTGCTTCTT-----CAAT-TCGAGGATAACAGCGAGATGCAAATTTTACAACGA
       ::  : :::::: ::::   : : : :  :::::      :::: : :::   : ::      :::::::  :    :: 
HYALO  TCACGGATACTATCTGCAGACTCGA-CACCTTCTGTATGACAATGTTGAGTCCATCAT-----TGCAAATGATTAGGCGC
```

PITG_11109
Waterman-Eggert score: 261; 27.4 bits; E(1) < 0.066
49.7% identity (49.7% similar) in 3092 nt overlap

```
     130       140       150                  160        170       180       190
PINFES CACAATGTCATCATCTACAATC--------TTGAGT---CCAAGGAG-CAGCAGTTTATACATGGCGTCGAGACTACGGG
       :::: :  : :::  ::: : :        : :::    ::: : :: ::  ::::: : :::: : : : : : : :  
HYALO  CACAGTCGCTTCAAGTACCAACCATATCGATAGAGAATGCCATGAAGACACTAGTTTGTCCATGAC-TGGTGGCAAGGAC

       200       210       220       230         240       250       260
PINFES GCGCGGTATCACGGCACTCGCCGTGTCGCCCAATAAGCGCT--ACGTGGCCGTGGCGGAGCAGGCGGAGC----------
        ::::  ::    : : :   : : : :  : : :: : ::  :::: ::  : :  ::   ::  : :::          
HYALO  ACGCGAAATGT--GTATT---CTTTTGGAGCCAGAAACTCTTAACGTTGCGTTTG--GAAAAGCGGCAGCTATATACTGA

          270         280            290       300        310       320       330
PINFES -GCGCGGTTGT--GCACATCCAC---GAC--CTCAATACGCTGC-GACGCCGCAAGACGCTAACACCTAGTGACGGTCTG
        :::::: : :  : :::  : :   :::  :: :::     ::: ::::: :::     : : :::: :::     : : 
HYALO  AGCGCGGGTATAAGTACAGACGCCGTGACAACTGAAT----TGCAGACGC-GCA-----CAATCACC-AGTACAAATTTA
```

```
        340       350       360           370       380       390       400         410
PINFES GCGGAAACTTTTGTCTGGGTGGCGTTT----TCAGGCGATAGCAAGTACTGCATCACGCAGGGAGGCGCAC--CAGACTG
         :::::   : : :  :  : :: :     :: : ::::  :::: :::   ::: ::::  : :: : :  :: ::: 
HYALO  TTGGAAA---TGGACATGAAGCCGCTAAGCGTC-GACGATC-CAAG-ACTC--TCA-GCAG--AAGCACTCTGCACACTC

              420       430       440              450       460       470       480
PINFES GGTGCTTTCGCTCTGGTTGTGGGAGAAGACCAAGCTGC-------TCGCGAGCGTCAAAGCCTCAACGCCTCAAGGCAAC
       ::       : ::::  : :  :::  :::  : :: :       :  :: ::  :: :    :::   :: :  :::::
HYALO  GG-------GTTCTGAATATACGAGTGGACGGA-CTACGACACATTTTCGTGC--CATA----CAAGAGCTGA--GCAAC

           490       500       510          520                530       540
PINFES GTCATTCATCAGGCAGATTTCAGTCCAAATGAACC---TCTAACGAT---------ATGTGCGAGTGGCAACGGAC---T
       :   :   :     ::: : ::   :::::  :::   :: : :: :         ::::::: :  :::: :: :   :
HYALO  GAGGTGTCT-----AGAATCCAACACAAATCCACCGTGTCGACCGCTGCACAGCGCATGTGCGTGAAGCAAAGGTCCAGT

       550       560       570       580       590                600        610
PINFES GCTAAAGTTCTTCCGATTCACAGACGGCCAGCTGAGATTACAGGC--------GACGC-CGCTGA-AGCGTGA-----GC
       :: : : :  :: ::   : ::::  :  : :::    ::: ::         ::: : :: ::: ::  : :     ::
HYALO  GCGACA-TCATTTCGTAGCTCAGAAAG--ATCTGCTGGTACGGGATGCACAATGACTCTCGGTGACAGTCTCAAGTCTGC

           620       630       640        650       660       670       680        690
PINFES CCGCGCACTTCCTACGACATGCTTGGGTCTCG-GACGACCGCGTTGTAGCAGCAGCAGACTCAGGTGAACTGTG-GCTGT
        : : ::   ::: :: :: :::   :::::: : : : ::    ::: ::::::::  : ::     :: ::  ::   
HYALO  ACTCACAAGACCTTCG-CA-GCT---GTCTCGAGTCAAGCG----GTAACAGCAGCAACCGCA-----ACAGTATGCCAG

             700         710         720       730       740       750       760
PINFES TCGAACAGTTTGA--GTTTAGACAG-ATCT-TGACGCCAGCGACTACTGGCGCAACGGGAGACGGCAGCTACGTGAGCTC
        :::    :   :  :: :  :: : :: : : :: :  :: :  :::  :  ::::     :: :: : ::   ::  :
HYALO  ACGATACCTGCCACGGTGTCAACTGCATTTCTCACTCTCGC-ATGACTTCCTGAACGTCTC-CGTCA-CCAC--AAGTAC

       770        780       790       800       810       820                830
PINFES CTTGTTG-GGCTACTCCAAGGGCTTCGTTTGTGGCGGGTCGGGAGGTTGTGTGCGCATT---------TACGACCGAAGC
       ::: ::: :: : :: : :   : ::   :::: :: : : :::     ::::::: ::         ::: : ::  ::
HYALO  CTTCTTGCGGTTTCTTCTACA-CGTCTACTGTGCCGAG-CTGGA-----TGTGCGCTTTGCCCTTGTATAC-ATCGTTGC
```

```
       840         850            860       870       880       890              900
PINFES GAT--GACGGAGGAT-CGCG----CGAGTACTACAAAAAGGCAAAAACGTTTCGAATCGA-------GGGTGATACTAGC
         :  : :: :: :: :: :    : :::  : ::: :  :  :::::::  ::::::         : ::: ::: :::
HYALO  ACTCTGCCGCAGCATTCGAGTCTGCAAGT--TTCAACAGTGACAAAACGTAACGAATCAGTAATTCCGCGTG-TAC-AGC

           910       920           930        940             950       960
PINFES ACTATCAAGGACTTGGCTATCAGTCC----TAGTGAAG-ATCTGCTCG------TTTGCTCCCTTGAGAA----CAACCA
       :    ::::::  ::  :: ::: :     :: ::  : :  :: : :      ::   ::  : : :::    ::  ::
HYALO  AGCTGCAAGGAGCTG--TACCAGACGCACGTATTGCTGCAAATGGTGGGAGATATTGATTCTGTCGGGAATGTGCACGCA

      970       980        990      1000      1010      1020      1030      1040
PINFES TATGTACGCACTTACAC-TCTCAAGCACCGATATCCTCAAGGAAGACGCCATGAACTTCGAGGTCGTCTCGACGGCTTTC
       :: : ::   ::: : : :  ::: ::::    :: :: :: :  ::   :  : :: :  :   :: :  ::
HYALO  TA-GCACCGTCTTGCCCATGACAAACACC----TCGTCCAGCATCAC---AGTAGCTCCCTGTATGTATATACA------

      1050      1060      1070      1080       1090         1100       1110
PINFES CATGCACCGAGTAGCAGTGGGAGCTGTCATATC-ACTGGACTTGACACG---TGCATTCG-TAAGCCGCT-----AGTGG
       :::: : ::: :::::     ::  :::: ::: : :: ::: : ::::   ::: :::  :::   ::      ::: :
HYALO  CATGAATCGAATAGCAACA--AGTCGTCA-ATCTAATGAACTAG-CACGAGTTGCTTTCTATAAAAAGCATAACAAGTAG

      1120       1130       1140      1150      1160       1170      1180      1190
PINFES TGACTTGTG-CGCTC-GATCGATCTGTGCGCGTGTGGAATTACTTG-GACAAGTCAACTGATATCCTTAAATTCTTCCGC
          :: : : ::  : : : : : ::: : ::   : :  ::   : :  ::::: :    : :: :: :   : :::
HYALO  CTTCTCGAGACGTACAGTTGGCTTTGT-CTCGCAGGCATCTAAGAGCGTAAAGTCCA----TGTCATTGACGACCTCCTG

         1200            1210        1220       1230       1240      1250        1260
PINFES GAAGACGCTTTG------TGTGTGG--CCTTCCATCCATCCGG-CTTGCA-CGTCGCTGTGGGCTTTA-CAG-ATAAGCT
        :  :: : ::       :: :: :  : ::   :: :: ::  :  ::: :  : :   :  ::: : ::: :  :::
HYALO  CACCACACCTTCGATCACTGAGTCGTTCTTTAAGTCAATACGCACACGCAGCCCCACAAAGCTCTTGAGCAGCAGCAGCA

           1270       1280      1290      1300      1310      1320      1330      1340
PINFES ACGATTGCTCAACGTC-CTTATGGACGACATTCGCTCTTGTCGTGAGTTTACTATCAAAGGCTGTCGAGAGGTGCGTTTC
        ::       : :::  :::: ::  : : ::: : ::   : ::  :: :: : :   ::  ::::  :  :::
HYALO  GCG-------ACCGTGGCTTACGG--GTC-TTCCCCCT--CCTTGGCTTGACCACCTC-GGAAGTCGTCACCTGCA----
```

```
            1350      1360         1370        1380      1390      1400
PINFES TCGAATGGAGGTCAGTTCTT---CGCCGCAGCAAACAACA--ACGTCGTCCATGTGTATGCCAC------ATA--CACAG
       :::::: : :  ::  :  :   : :: ::  ::::::::  :  :: :: :  :  :  ::::      ::   : ::
HYALO  TCGAATCGTGCACAAATAGTGTACACCACACAAAACAACACTATTTCATC-ACATAAAAACCACCCCACCATCGTCCCAC

     1410      1420      1430                1440           1450      1460      1470
PINFES GGGAGTTGGTGTCCGTGCTACG-------TG---GCCATTCGAATCGCGT-----GACGGCGATTTCATGGAAATCTGGC
       :  ::::  :::::     :::       ::   ::::::   ::  :::     ::::::  :  ::: :    ::  :
HYALO  GA-AGTTCATGTCCACCACACGACATTCCTGTGCGCCATTG--ATGTCGTACTTAGACGGCC-TCCCATCG----CTTTC

          1480          1490      1500      1510       1520      1530      1540
PINFES GACCGAA--AGCTACTA--ACATGTGGAGCTGATGGTACAGTCCTC-CTCTGGAGTCTCCGTTCAGCATCAAA-AGCTGG
       :::  :   : : :: :  :::     :: :::   :: : : ::: ::: :     : : ::   ::::  : ::
HYALO  GACTCACTTAACAACGACGACAA---AAGTTGACCCTAAACTTCTCGCTCGGT----TACATTTTCCATCGCACAGTACT

      1550      1560      1570      1580          1590      1600         1610      1620
PINFES CGATGGCCACTCACAGGCACGTTGTTCATACATTGATGC----CGCGCTATCTCCAGAAGGGG---ACGTGGCGTACGTA
       :: : ::  :: : :       : :  ::: : :::  :    : :  :::  :::: :       :: :  : ::: ::
HYALO  CGCTCGCTGCT-ATA-------TATATATAGAGTGAGACTAAACACATTATAACCAGTAACCCTCTACCTATC-TACCTA

             1630      1640      1650      1660      1670      1680      1690
PINFES ACAGGCTCTGATAATATGCTCAAGGAGATTGATACGGCTACGGGTTTGGCACGAAACGAGCTTTGGGTCGGCG-GTAGTG
        :   :: :    :::  ::::: :  ::  ::::  ::::    :    ::  : :::::  ::::::  :: :  : :
HYALO  TCTACCTATC--GATACACTCAAAGTCAT--ATAC--CTAC---CTACCTAC--AGCGAGCGATGGGTCCTCGCGGCGCG

    1700      1710         1720                1730               1740      1750
PINFES GGGCTGCATTAG---GCCCATTAGCTC----------TCGCAAACTCACAGC---------AAGTGCTTTTTGTCGGA--
       :: : :: :  :   : :::  ::: :          :::  : :   ::::         :::::      :: :::
HYALO  GGACGGCGTCTGAACGACCAGGAGCGCATGGAGATCCTCGAGATCATCCAGCGCGAAGCCAAAGTGAAGAACGT-GGACC

        1760       1770      1780             1790                    1800
PINFES TCGGCA-GAGCCAGGTCGTCCCGGTA----CGGT---GAGGCT--------------ATACCGTTTGCCAT---------
       ::: :: : :: : : :::  ::: :    :: :   :: :::              : :: ::  :: ::
HYALO  TCGCCAAGCGCTACGGCGTGTCGGAAGGCGCGATCCGGAAGCTCAAGCAGATGCAAGACACGGTCCGCAATCGCTATTAC
```

```
          1810        1820           1830      1840          1850           1860
PINFES -TCGAGAAAGACCAC--CCGCTAC-----CAACAACGACGTTGTTGAGTG----GTGGTGTA-----ACTACTTCTCCGG
        : :  :: :: :::  :::: ::     :::   :: ::   :  :: :    : :  ::       :  :  ::: :
HYALO  ATGGGCAACGAGCACAACCGCGACAAGCGCAAGCGCGGCGGCTTCCAGCGCAACGCGCCGTTCGAAGAGGAGCTCTACCA

      1870      1880      1890      1900      1910      1920         1930       1940
PINFES GATCTGTATCACCCACAGCCCAGACTGGATCTACGTTAGCTGCTGCTTCTGCAGGTACAG---GCTCAGCGTCC-CAGGC
       :   :: :::   : ::  :  :: : :  :  :  :: : :::  : : ::    ::::   ::   :::::  :::::
HYALO  G---TGGATCGTGCGCATGCGCGAGTCG--CAGCCGTACCAGCTCATGCCGCTCACACAGACGGCGGTGCGTCAACAGGC

                1950      1960      1970      1980       1990      2000      2010
PINFES C------TACACTGAGTACCATTGCCATGACCTCCCGGTGTCACGACT-TCGACTATCGTTCGACAACCAGTTCCTCTTT
       :      :   :: :: :: :: : :: ::   ::   : : : : :: ::     : ::: :    :  : : :: ::
HYALO  CATTGTCTTGGCTAAGAACTAT-GACAAGATGGCCAATTTTAAGGCCTCTCCTGGCTGGTTTG----CGCGCTTCTGTTC

       2020      2030        2040      2050      2060                    2070
PINFES TCTACAGGCGAAGATGGATCG--TTGTGTATCTTCGAGACGCAAAA-------CGTCATCGG-------AAAAG------
        :  ::  :::   ::::::   :::::      : : :: ::           :: :  :::       : :::
HYALO  GCGCCAT-CGA--CTGGATCCAATTGTGACCAATGGGGATGCTGCTGCAGCTGCGGCTGCGGCTAGTGTACAAGTGGCTG

         2080        2090      2100      2110         2120      2130      2140
PINFES GAGGAAGCGTTGGT--GCTAGTAGCGGTCCAGGAGGTGGCACT---GTTGGGTCTCGAGAAGCTCGAAGTGATAGCAT-G
       :::  :::  :::   ::: :  :::: :   :  ::: :::    : :::  :  ::  :   ::: : :::: :: : : :
HYALO  GAGACAGCAGTGGGACGCTGGCGGCGGAC---GGCGTGTCACCAGGGATGG--CTGCATCTGCT-GCAGTGGTACCGTCG

     2150      2160      2170      2180      2190       2200                  2210
PINFES ACACTTGGTGGCGGTGGTGCTAACGGCTCGGGTTTACCCTTTGCAG-AGGAAATT------------CTGGTTACCAAAT
       ::    :  : ::: ::     :::  :::: :::::   :::: : ::: : :             :  :: :: :  :
HYALO  AC----GACGACGGCGG-----ACGATTCGG-TTTAC---TTGCTGGAGGCACTCACAGAGTCGTCGCCTGTGACGACGT

             2220         2230      2240      2250      2260      2270      2280
PINFES CG-----GATCTAGA---AGAGAAGAATCGTCTTATGAACGAGCTGAAGTCCAAGGTGGACGAGTTGACGCTGCACAACG
       ::     :::  :::   ::::  : : :: ::         ::::  :::   :: :::  :   ::::: :  ::: :
HYALO  CGCAGACGATGGAGATGGAGAGC-GCAGCGACT---------GCTGTGGTCTTGGGAGGAGCATCGGACGCAGAGCAAGG
```

```
       2290              2300      2310      2320              2330       2340
PINFES -AGTAT--CAG------CTTCGATTAAAGGATCTCAACTATAAG--------GAGAAGATCAAAGAGG-TATCCGACAAG
        ::::   :::      : ::::: :: : :      :   :::        ::: : : : ::: :: : : :::
HYALO  GAGTAAAACAGGGACGCCGTCGATCAATGAACAGGTGCAGCAAGCTGCTCGCGAGCATAACGAAGCGGCTTTGCGA----

    2350      2360      2370      2380         2390      2400      2410      2420
PINFES TTCACCGCTGAGCTAACGCAAGATCGACAACGCT---GTGGCGACTTGCAGAGTGACAAGACGGAAATGGAGAATGAGTA
             ::::   :  : :::::: : : : :::   ::: ::   :: ::: :  :::: :: ::  :::: : :::
HYALO  ------GCTGCCGTGGC-CAAGATTGGCGAAGCTCAAGTGTCGG--TGGAGATTATCAAGTCGAAAGAGGAGGAGGAG--

       2430      2440      2450      2460      2470      2480      2490      2500
PINFES CCAGAAGAAAATGCGCGAGATGGCTGCAACGCACCGAAGTGAGATACAAGAGCTGAAAACCACGTACGAAGCCAAAGTCG
          :: ::::: :       :::  :  : :      : :::: :: : ::   :: :         ::::   :::  :
HYALO  ---GAGGAAAAGG-------TGGACGTCAAG------ATTGAGGTAGAGGACGAGACA---------GAAGAAGAAG--G

       2510      2520      2530      2540      2550      2560      2570          2580
PINFES AGGCTGAAAAAGCACGCTACGCAGCACTAGAACAGGCACGTGGTGAGCAGAACACTCGGTTCGAGGAGGAAA----GTCA
       :::          :::  : : ::::  :: ::::  ::: : ::   ::    :: ::  : :   ::::     ::::
HYALO  AGGG---------ACGGAAGGAAGCA--AGGACAGCGACGAGATGTTTAG---TCTTGGA-CTATAGGGAATACTTGTCA

                        2590       2600      2610         2620        2630      2640
PINFES -------------TCTACTCGTTG-AGAGCCACACACAATTCCTAGCG---GAAA--TGACAACCGAGTACGAGCAAAAG
                    ::: :: : :: ::::   :      ::: : :::   : ::  ::: :: :::     : : :
HYALO  ATCATTTTAAGGTTCTTCTTGGTGCAGAGTATC------TTCGTCGCGATCGGAACTTGAGAATCGA-----AGCGACA-

          2650      2660      2670           2680      2690      2700       2710
PINFES TTCGCTCATGAGCAGGAATTGCAGGCAGTCTGTG-----AGCAGGCTAAGAATGAGCTCATTGCCACAAA-TGAAGCGCT
             :::::        : ::::  :: : :      :::::  :  :  ::::     ::  : ::: : ::: ::
HYALO  ------CATGA--------TACAGGTCGTGTTTTTCTACAGCAGCATTTGGTTGAG-----TGGAAGAAAGTAAAGAGCA

      2720      2730      2740      2750       2760      2770      2780      2790
PINFES GAAGCGTTCTCTTGAAGAGGATGCAGAGCTCGAGGTAG-ACGAGCTGAAGAGTAAGTACGAGCTGAAGCTGTCAGACGAG
       :    ::::        :::   ::: : : : :: :: :::: :    :: : :    :::::: :::::     ::
HYALO  G----GTTC--------AGGCGACAG-GATGGTGGAAGCACGAAC----GACTCA---TGAGCTGTAGCTG-----CG--
```

```
       2800      2810      2820             2830      2840
PINFES CGTGAGGCGACACTACGCCTTAAAG----GCGAA---AATGGCATTATGAAG
        : :: : :   :::    ::::::    :::     ::::: : :::: ::
HYALO  -GAGATGTGTTTCTA----TTAAAGCGCTGCGCTGCCAATGGTACTATGCAG
```

PITG_18065
Waterman-Eggert score: 283; 61.3 bits; E(3501) < 5.1e-09
58.8% identity (58.8% similar) in 311 nt overlap

```
          160        170       180       190       200         210       220       230
PINFES TGGATTT-GACAGCTCCAGTTGTTGCTCTCGAATGGGACACTGAAGGCAGTACAC--TCGCCATTCTACAACAAGGGAGT
       : ::: : :::  ::::  :  ::: ::::::  :: :::: :: :::::: :    ::  ::: :: :  :::   :::
HYALO  TTGATGTAGACCTCTCCTCTATTTGTTCTCGATCGGTACACGGAGGGCAGTCCTTGGTCATCATCCTGCGCCAAAACAGT

             240        250       260       270       280       290       300       310
PINFES AGTGTCGTCCCCCTA-TGGGATAACGGCACACGATCAACGTTGAGTCTTGACACGAATCTCAAGGACCCGACGTTTGCTC
       :   ::::: :  :  :: :: ::::: ::           ::  ::: : :: : ::  :    : :::  : : ::::
HYALO  AACATCGTCTCATTTGTGTGACAACGGGACC----------TGGATCTCGTCATGCATACCGGATATCCGT-GATCGCTC

              320       330       340       350       360       370       380       390
PINFES GGTGGTCCAAAGAGGGTTCGTTACTAGCAGTGGGGACTCAGAAGGGGAACCTCGTTCTCTACAGTAAAATCACTCGGAAG
         :  : : :  :::::: : : : :::   : :: :  :::::  :: : :: : :: :::  : :: :::: ::
HYALO  TATTTTTCGAT-AGGGTTTGCTGCAAGCGACGAGGTCAGAGAAGAAGA-CATCCTGCTTTACGCTCAAGTCACGCGATCA

              400        410         420       430       440       450
PINFES CTCGTTCCTGTGGTGGG-GAAAC--ACTCGCGTCGCATATTATGCGGATCTTGGAACGGAGACGACAAACT
       :: ::::   :: :: : ::  :  ::::::   :::  ::::  : :  ::: ::::: ::::::: : :
HYALO  CTAGTTCTAATGATGAGTGATGCGTACTCGCAAGGCA--TTATCTGCAGGTTGAAACGGTGACGACACAAT
```

PITG_08685
Waterman-Eggert score: 333; 44.4 bits; E(1) < 1.1e-07
54.0% identity (54.0% similar) in 609 nt overlap

```
               70        80        90       100       110       120       130
PINFES CTTGTCGTCAGCACGACTGGGTCCCCCCTTGGCGCCGCTTTTGGCGTCGTCCTCGTCGTCGCTGTTATAGTAA-GACTTC
       : :::: :: ::: :: : : ::    : :  :::::: ::::::::  :   : : ::: :::::  :  :: : :
HYALO  CATGTCATCGGCA-GAGTAGCTCTGATCATT-CGCCGCATTTGGCGTTTTTACCTTTGTCACTGTTTAACGAATGGCGAT
```

```
          140       150       160       170           180       190       200       210
PINFES TTTGCCTGCGCTCTGTCCTCTGGAATGTATTTGTACT----CGGGCCTTGTTGTGCATGTATTAGATACGTCTCCTACAC
        : : ::::   ::::: ::::  :::::  ::::::    :  :: : ::::     :  ::: :     :::: :: :
HYALO  GTAGTCTGCTTCCTGTCGTCTGAGATGTACCTGTACTGACCCATGCATAGTTG-----GATTTAAA-----CTCCAACGC

            220       230       240       250       260       270       280       290
PINFES AGTAGAAGCTGTCGGAGACCGTGGCGTAGGGCTGACCCACCATGTCGCTGATCAGTTCGTCGTCCAGTGGAGGCATG-GC
        ::::   :::::  ::::: : : :::      : :::: : :  :  :  :  :  ::::         :: ::  ::
HYALO  CGTAG---CTGTCAAAGACCATTGTGTA------AGCCACTACG--GGGGCCCTATCTGTCG---------GGGATAAGC

             300       310         320       330       340       350       360        370
PINFES ATAGATGGCCTCCCACGTGTT--CACACTCGACGGAATGCAGAAACCAAAGTTAAAGTCGTAGCTCTTAA-CGATCTCGT
        :: ::  : :: ::::: :   : ::::   :  ::: :  :   ::: ::::  : :  :::: ::   :::::::::
HYALO  GTAAATATCTTCGCACGTATATGCGCACTTCGCATAATACCAAGCACAACGTTAG-GGCACAGCTTTTTCGCGATCTCGT

               380       390       400       410       420        430        440
PINFES CGCGGAAATAGTGGCGCTCGATCATGCGCAGGTTATGCACCTCGTGTGGGC-CAACAGAGAACTCGA-GCTCCG--TGGC
         :: ::: : ::  :::: : ::: :   :::   ::  ::::::   :: ::: : ::     :: : : :   :: :
HYALO  TTCG-AAAAAATGATGCTCAA-CAT-CTATGGTCGCGCGTCTCGTGGTAGCACAAAAAAGGTTGTGATGATACAGTTGAC

       450       460            470       480            490       500            510
PINFES GATCGCCGGCAGT-----CGTAGCACGTCCTCAGAGAATTCGTA-----GCGAATGGTGCGGTACGCGC-----CCTCTC
         :: :   :::       :: :: :  :     ::::  : : ::     :::::    :  :::  : :     : :::
HYALO  CGTCACGTACAGCACGAACGAAGTAAATATAGAGAGCTTCCATATATAAGCGAAGTAAGTAGTAACCACTTCAGCTTCTG

            520       530       540       550       560       570       580       590
PINFES CGCGCGCATGCGAGATATCCATCTTAGGTACAGCCTTGCCTGGTCGGTCCTTACCGATTTCGAAAATCTTGCGCTTGGAC
          : : ::  :: :: : :: :  ::: :  :: :::  : ::::                  ::: ::: :: :: :
HYALO  TATG-GAATTTGATATGTGCACC--AGGAAGTGC-TTG--TAGTCG------------------AATATTGAGCATG-AG

            600       610       620        630       640
PINFES TCGAAGTCCGAAATGGAGAAGTGCAAGAA-CTCAATGCCATATTTGTTG
        ::: ::   :::   : : ::::::::: :::   ::  : ::: :::
HYALO  ACGATGT---AAA---ATACGTGCAAGAATCTCCGGGCTTTCTTTTTTG
```

```
PITG_5608
Waterman-Eggert score: 415;  38.6 bits; E(1) <  6.4e-05
50.5% identity (50.5% similar) in 2151 nt overlap

            890        900       910       920       930       940       950       960
PINFES GCGTAAATAAGGATTCG-ATTGGTCATCAGCTGCATCATTGGAGACCGCAGTCGTTGTGAGGGTGACGTACGGCATTTGT
       :::::  :   :::::: ::: :   :: :    : : :  : ::: :::      ::: :: : :   :: :     ::
HYALO  GCGTACTT---GATTCGCATTTGCGTTCCG----AGCTTGTGTGACAGCAA-----GTG-GGATAAAAAACAGG----GT

             970       980            990      1000      1010      1020      1030
PINFES GCTCTTGGAACTGGAAGCGAGA-----GCATCCT-CAAGTTGCATCCGTACACAAGAAATAATACGAACACAAGTGTGAA
       : : :::: : :: ::   :::     : ::: : ::::  ::::: ::  :   :::: :    ::   :::::    :
HYALO  GTTTTTGGGAATGAAAAGCAGACTGTCGAATCGTGCAAG--GCATCTGTTGAGGCGAAACACGCAGAGGCCAAGTTGCCA

        1040        1050                        1060      1070        1080        1090
PINFES GCGTAACGAA--GTCTTGCG-------------AAC-----AGTTTGGAGCTTGTTCCACTCG--TTGAC--AACCAAA-
        ::: :   :  : ::::::             : :     :: : : :::  : :: ::: :  : : :  :  :: :
HYALO  TCGTCATTCACCGCCTTGCGTTGGTCTTCGGTTATCTCGGAAGATAGCAGCGAGCTCGACTGGGCTCGGCGCATGCAGAT

                      1100      1110      1120           1130                 1140
PINFES ---------ATCATACGCCACACACTGATGGAAGCGTGGGTTGGCT-----TGGCGGAATC-----------GATATCCA
                :  :::       :::::: : ::  :::   :: :      :: : :: ::           :::::  :
HYALO  AGTGGACTGAAGATAATTTTGGCACTGA-GAAACTGTGTCCTGACCATGAATGACAGATTCTGTATACTTGTGATATTGA

          1150      1160             1170                1180      1190         1200
PINFES TGAC--GGTAGCGCGACGTTTCTCC-------CAGTCAAT----------GAAGAAGAGCTGTAGCT---GAGCTTGACG
       ::::  : :: :  :  : ::::::       :  :: ::          ::::::: :   ::  :   :: ::::  :
HYALO  TGACCCGTTATCA-GCTGATTCTCCTTTTTATCGTTCCATTGCAGGGCTGGAAGAAGTGTGCTACGTCAAGAACTTGG-G

          1210      1220            1230      1240      1250      1260      1270
PINFES ATAGACGTGATGGAACACTGA------CACCAGTTGACAGAAGAAGGATAGAGGAAGCAGCACTCGGAACGGGTAATATT
       : : :: :  : ::    :::      : :  ::::    :::::: :: :::  : :::  : :  : :  :: :::
HYALO  AGATACTTTCTCGACTGGTGAGTGATTCGCTTGTTGTATCAAGAAGCAT-GAGCTA-CAGTGC-CACATCCTGTTATAG-
```

```
      1280      1290        1300      1310      1320      1330              1340
PINFES CATCGACACCTCTTGC--AAGGGGGTCGAAATTCACCTCTGGAAGCATGAGAACG----GTGCTGGAC----TGGAGTCG
       :  : :: :   ::::  : :  : :    :::      ::  ::::  : : ::    :::: :::     :: ::::
HYALO  CGCCTACTCTATTTGCGCACGATGATTAGCATTGTTTGTTGAGAGCAC-ACATCGCTTGGTGC-GGATATCGTGCAGTCA

      1350          1360      1370       1380      1390      1400      1410      1420
PINFES AAAGACGA----TGAAAAAGTAGCTGCACATTATCA-ACATGACTGTAAATGTGACAGGGGCGAAGTAGGAGGCACTGTA
         :  :      :::  ::: ::  ::::    : : ::      : :: :  : :: :::::   : ::  ::: ::::
HYALO  TCATCCAGCAATTGAGCAAGCAGAAGCACCGAGTGATAC------GGAAGTTGGGCATGGGCG---TGGGTAGCA-TGTA

           1430      1440      1450      1460        1470      1480       1490
PINFES GCTGATCACGGTCGATAGAATCTGACACATGGCATTCTCCACG--ACTTCGTTCCGGATGCT-ACGTCTGTACCAGTTGA
         :  :    ::: : ::  : :::::    :: :  : :: :  :: :::: ::      :: :::::: :   :: ::
HYALO  ATTTTT---AGTCCAAAG--TTTGACAATCTGCTTAGTACATGCTACATCGTGCCCATCACTTACGTCTATGAAAGATGT

    1500        1510         1520      1530               1540      1550      1560
PINFES ACGTCTGAA--GGAG-GACGC--GACATCCTGCGAGGAC-------GCACCC--CACAGTGTACACAGCCTTTGCAAACG
        ::   :::  : :: :  ::  :::::      :: ::       ::: ::  :::: ::: ::     ::::   :::
HYALO  CCGCAGGAAATGCAGAGTTGCTTGACAT------AGCACACTAGCTGCAACCGTCACATTGT-CAT----TTTG---ACG

        1570      1580         1590      1600       1610            1620        1630
PINFES ACCAAAACTCGTCCGTG---TCGGAAGTGTACGCTACACGAA-GTGATA-----CTGCG-ACGACTC--TGGGACTCTGT
              :: ::: :::   :::  ::  ::   : :::::  ::::::     ::::: :  ::::  :  : : :::
HYALO  TT-----CTAGTCGGTGCTATCGACAGAATAG--TTCACGATTGTGATAGATAGCTGCGTAATACTCATTACGGCACTGA

           1640      1650        1660      1670                   1680      1690
PINFES GT-GTCGACATATGCTATCG--TTAGCTCCGGGACATAAATGAAGTGTG-------------GGTCGGACGCTTGTGTTC
        : ::: :  :  : ::::   :  ::::  : ::: :::    :::::              : : ::   :: ::: :
HYALO  CTTGTCCATTTGGGGTATCACCTACGCTCACGAACAAAAA----GTGTGACCCCATTATCGCGCTTGGCAACTAGTGGTG

      1700      1710      1720      1730      1740      1750      1760             1770
PINFES GGATAGTGAGTGTGATGGTTCCAGCGTATCGAATCACGTTGGAAGAGGTCTCGTCAACTGGAATTC---CCG----TTTG
       :  ::  : :::::: :   :::  :   : :::        :::  ::::   :: ::     ::    :::    ::::
HYALO  GTTTATAGTGTGTGAGG---CCAT-GACACCAAT--------AAGGCGTCTGTCCAGCTTCTTTTTGTACCGGATTTTTG
```

```
             1780      1790      1800      1810        1820      1830      1840
PINFES AAAGTCGTACAGGAAGAAGCGATGAGATAGAAAATCGTTTGCTATGTC--GCTATCCTGATTGACGAACACTCCACTGCT
        : :::   ::: : : ::   :   :: :  :   :  ::: ::: :  ::  : :  :  :: : ::: : ::: :
HYALO  CAGGTCCACCAGCACGTAGATTTTC-ATCGGCATATGAATGCAATGACTGGCAGTGCAAAGCGA-GGACA-TGCACCGTC

     1850          1860          1870        1880      1890      1900      1910
PINFES GTCACG----ATAGTTTA----AATTCCTCACAGGA--ATGGGAATGAACTTCTTATCGTCTCCACTGCTCGTCGTATCG
        :  ::    :::: : :    ::::  :   ::::  :  : :: ::: :  :::                ::: : ::::
HYALO  CTAGCGCAAAATAGCTGAGCTCAATTTGTGCAAGGATCAACGCAA-GAA-TGGTTA--------------CGTAGCATCG

     1920      1930      1940      1950       1960      1970      1980      1990
PINFES ACCAAGAAAATCTCGTGCAGAATTAGCTTGGAAGT-CTGTAAAGCCTCGAGATTGCACGAATATTCCGATACTGCGCTCA
         : ::::        :  : :::  ::  : : : :::: :  : :  : ::: :: ::::::    : : :::::  :
HYALO  --CTAGAA--------GGCGCATT--CTGAGTATTACTGTGA--CTTGTAAATTACA-GAATATCAACAGAGTGCGCGTA

       2000      2010      2020      2030      2040      2050      2060       2070
PINFES C-TCCAAAGTGCATCCACGAAGGAGAATCAGCCACGCCAACGCTGCCTGGTTGACAGTAGGCCAGTTCAT-GTGTCAGCG
       : :    ::  ::  : :: : :     :: :::   ::  :::   :: ::    ::    :: :::::  :: :: :
HYALO  CGTGTTTAGCTGATGAA-GATGTACTGGTAGACACAATAATTCTGTGAGGCTGT--ATACATCA-TTCATAGTTTC-GAA

        2080      2090      2100      2110      2120        2130      2140       2150
PINFES GTCGAAATCCGATGAGCGTGCCGTTGACGTGATAGCTGGCCAGTACGAA--CTCAAGATGATCAATGCCTTCAT-TCGAT
       :: : : :  :::   :    ::   ::: ::      :: :  :: ::  :: : :::: : :::    :::: : ::
HYALO  GTAGGAGTTAGATTGTCAGCACGCAAACGAGAGT----GCAAACACAAAGACTGATGATGTTGAATAGAGTCATATTGAG

           2160      2170      2180      2190           2200      2210      2220
PINFES GCCGCGTCCGAAGTCATCTTCATGCCTACTGGCGTCTGA---AGTACAC--TGGATGCAACCGACCGATAGTTCAAGAAT
       :       ::: :::  :  ::::: :::   :    : :   :::  ::  ::::   :::::  :  :: : ::: ::
HYALO  GAG-----CGATGTCGACAACATGC-TACACGACATTAAGATAGTGAACGGTGGAGCGAACCGGACATTATTGCAACAAG

             2230      2240      2250                         2260        2270
PINFES G-------GCAACGTGGTGAACCACCCGTC-------GATGTTGT------------TAA--CTATTGACGATCGTCCAC
               : ::  : :::::  :: :::        :: :: ::            :::  ::: :::  :: : : :
HYALO  TCACAGCCGAAAGATCGTGAAGAACACGTTACAGCATGAGGTAGTCTTTTGTATCGATAATTCTAATGA--ATTGACTAT
```

```
     2280        2290      2300      2310      2320      2330      2340      2350
PINFES T-GCGTTGA-ATTGAGTCGAAAACAGCGCAGGAAGGTGCTTGAGGGTCGAAATGTTGCAGAACGCAGAGGTTCCCCAGTG
       : ::::::  ::::   :: : :::::::: :::: : ::    : :: :: :::: :: : : :::::  :::   : :
HYALO  TTGCGTTGTTATTGC--CGGATACAGCGCATGAAGATACTGTGCGATCAAATTGTTCCATAGCACAGAGACTCCAACGCG

       2360      2370      2380      2390      2400      2410         2420       2430
PINFES CCTGACACGACACGATATCTTTCTCGCTTTGGTAAAAGTAACATGCAGTGGTGGCAGA---CAGAAACAT-GTCGTCAAG
       : ::: ::::  :: : :    :: : :::  : ::   ::::  :  :   ::::: :   ::::::: : ::: :  ::
HYALO  CATGAGACGAATCGGTGTACCGCTTGATTTTCTCAAC--AACAGCAACTTATGGCAAAGTGCAGAAACGTTGTCATTGAG

           2440          2450                      2460      2470            2480
PINFES AACAGCA----CTAGCGACGGAAACGA----------------CCGACTCGAAGCTGCCAG---TTTC---TTCCTTCAG
        ::::::    : ::::::  :::::                 :::::: :::  :: :     : ::   : :  : ::
HYALO  GACAGCATGTACGAGCGACA-AAACGCGAAGCCTGGAGTGCCTCCGACT-GAAAATGGCTCACCTCTCGGTTGCGCTGAG

       2490      2500       2510               2520            2530      2540      2550
PINFES GAAGGACGAGAACGTCATG-TCCTTCGCGT--------TGT-TAAGTGTG------ATCTTGGAGCTGATCAAGTTGATG
        : :  :::   :: : :: :  :::::::        ::: ::::: :       :: :   :  :  :::    :::
HYALO  CACGACCGAAGCCGCCTTGCTTTTTCGCGTCGCTCTTTTGTATAAGTTTACTTCACATTTGTTATTTACTCA----GATA

             2560      2570      2580      2590      2600       2610      2620
PINFES TGTGTATTGCGAACACACTTTTGGACACTCATCAATAAACTCGTGACCTGCTGAT-AGTCATTCGGACAG----------
       :: :::   :::  ::: : : : :::  :: ::: :  : :   ::: : :  : ::: ::::  ::
HYALO  TGCGTACATCGATAACA-TATCGAACAAACACCAA-AGGCGCAACACCAGGTATTTAGT--TTCGTCCACCGTTGTGCTT

               2630          2640      2650       2660      2670      2680      2690
PINFES GCACACAC-GAA--GCTA--TTGCTAGCTACAATCA-TCATTGGATCTGGACATGGTTTGCACGTGTAGGTGGAATAGTC
       : ::: :: :::  : ::  :  :::  : :: ::: ::::   : :  :: ::: : : ::::  : :::   : : :
HYALO  GTACAAACAGAAATGTTAAGTGTCTAT-TTCAGTCAGTCAT---ACC--GAGATGATCT-CACGCTT-GGTTCTACAATT

          2700      2710      2720      2730      2740      2750      2760
PINFES AGCGACTGTGTTCGCGGCACATTCCACACATTCTTTTGCGTCGAGATAGCTGCCATCCTCGTTTTTCTCTT
       :   :: :: :::  :       :::      ::: ::::  ::  : ::     ::: :::::::   :::
HYALO  AC--ACAGTCTTCTTGCTGTCGTCCTGCTTCTCTGTTGCACCGGCACAG-----ATCATCGTTTTGAACTT
```

PITG_3600
Waterman-Eggert score: 868; 73.9 bits; E(1) < 1.8e-15
53.3% identity (53.3% similar) in 1799 nt overlap

```
          510       520       530        540       550       560        570       580
PINFES GTCTCCTGTGAATTCACGGAGAT-GTTGAGCCGCGAGTTGTTGCGAAGCGAACGGAA-CATTAAATGGGCTTGAGAACTA
        :: : :::   : ::  :: ::: :::::   :::::  :     :  : :::  :   :::  :  :::::::     ::
HYALO  GTATACTGACTAATCTAGGTGATTGTTGA---GCGAGACGGCTGGGGGTGAATTGCTGCAT--ACGGGGCTTGCATGGTA

            590       600       610           620       630       640       650
PINFES TCTGGGATCAACTCGGCTAACACAA----AGGCGTTGTCCAG--CCATGAGTACATTTGCTGTGGCTTCAC----TGGCG
        ::  :::       :::  :::::     :  :: :::  :    : :::   ::  :: : :  :  :::      :::::
HYALO  TCGAGGA-------GGCGGACACATTTACAATCGGTGTTTATGACAATGGACACTGTTTCCGAAG--TCAAATTATGGCG

              660       670       680       690       700       710         720       730
PINFES --TATTGAAACGCACCGATCCTTCCGGCGTCGTCTGTGGGCCTGGAGTTTCACCAGTGATGT--GGTGAAACGCAGCAAA
          ::  :: ::: :  ::      ::: :: : : :  : : :   ::     :::  : :::   ::: ::: :: :::::
HYALO  GGTAGCGAGACGGAAGGA----ACCGTCGACCTTTTCGAGACAA-AG----ACCCAT-ATGGCAGGT-AAAAGCGGCAAA

                  740       750        760       770       780       790       800
PINFES CTT--TGGCAGTTTGAAAGTGGATTCA-AAC-ACGTGAAAGGACGTGTGATGGCCACGTGAACCTACGAGCACCTTGAAA
       :::  :: ::   :::: :::   ::: ::: ::::: :  : ::::::::::            :  ::    :::
HYALO  CTTGGTGACA---TGAAGGTG---TCATAACGACGTGGAGTGTCGTGTGATGGC-----------ATTAG----TTGGTG

          810       820       830          840       850        860       870       880
PINFES TCGAGACGCGCTGCAGCATCTTTAG---TCACATTCAGCGGAATTCGAACTG-TCGAACTCATAGTTGCTGGACGAACGA
         :: :::: :::      : :: ::   : ::        :::  ::::: : : :: :  :: : :  ::: : :: ::
HYALO  ACGCGACGTGCT------TGTTCAGGGGTGAC--------GAAAACGAACCGATAGAGCA-ATGGCTATTGGTCTAA-GA

              890       900       910       920       930       940         950       960
PINFES CCAAAGCCTCGCCCTCGAAGATACCCGCTTCGGACTCAAACACAATTACCATCTTGATAACTG--CGTCGTTCTCGGTC-
                : ::  : : : :: : : : ::  :: :  :::: ::  :  ::  :  : ::   :::: :: :: :
HYALO  --------TAGCGGTTGCACATGCACACCTC--ACACGTACACGATCTCGGTCGGGGAATCTTAACGTC-TTATCAGAAT
```

```
                970       980       990      1000       1010      1020      1030
PINFES ---GACACTTCGAGCTCAAAGTTTGGGGCGCTTTTACTTGCTAGTGAG-CTTACCTTGAGGTTGGTGTCGGCTCGAATAC
          ::  : :: ::::   :::::: : :    ::  ::: :   ::  :   :::    ::: ::::  :: : ::: :
HYALO  TGTGATCCGTCAAGCTTGGAGTTTGTG-CAAGCTTCATTGATCAAGAAACGCACCCCACAGTTCGTGTTAGCGCAAATGC

      1040      1050      1060      1070      1080      1090       1100         1110
PINFES GTAGCCCTTTTCCTGCTGTCGCAGTACCGGCCTGGTTTGCCTCTAACGACTTGATT-TCCAGCT--GTAGCGC-TGCTCG
       : : :: : :::::::::: ::  :::              ::: :: : :::: : : :: :   ::::    :: :::
HYALO  GCAACCTTGTTCCTGCTGT-GCTCTAC--------------TCTTACAAGTTGAGTCTGCAACCTCGTAGATTGTGATCG

          1120      1130      1140      1150       1160      1170      1180      1190
PINFES ATGTTTGGACAGTTTCGCTACCTCTTTCTCTTCCTGACTTGC-CTTATCATTGGTACCAGTACTGGACACTCCTGGGGCA
         : : ::: :  :: :: :: :: : ::  : :: : :::: ::::::  :::        :::   : :   : : :
HYALO  GCGCTGGGATAACTTTGCAACTTCCTGCTGCTTCT-AGTTGCACTTATCGATGG--------CTGATGAATGTGGTGCCG

           1200      1210      1220      1230      1240      1250        1260      1270
PINFES GGAAAATCACCCGTGGCAGTGAAATAGCCGCGAATCTCTCCGTCTGCTGAACAGCAGA--TAATCTCGTCTTTGCCATTC
       :::::::   ::::    ::::::  :::  ::  ::::::  : ::::: ::::::   :: :::: ::  :::
HYALO  GGAAAATA--CCGTAATCGTGAAACGGCCAAGAGCCTCTCCAACAGCTGAGCAGCAGTCCTATTCTCCTCCGTGC-----

             1280      1290      1300      1310      1320      1330      1340      1350
PINFES ATGCGATAGTCTGCCGTCACAAGGGCCGCCACAGGCGATTTAAGCACTTCCTTATGCACGGCTTCTCCGTTCGCCAGCTT
       : :::::  :: :: :::::::    ::    ::        ::::: :  :: :: ::: :    ::   :: :: : :
HYALO  ACGCGATGATCCGCTGTCACAAATATCG----AG--------AGCACATGTTTGTGTACGCCACGCCCC--CGTCA-CGT

             1360      1370        1380      1390      1400      1410      1420
PINFES TCGCGCCTCGAACTTGCCGTTGC--TCCAGCCCATAACGATCTCCTTCTCTCCATCACCGTCGATATCAAAGCTGTGGAT
       :   :   :::: :::: : :::  :::  : ::: :  :: : ::: ::  : :: : :::::::: :::::: :::::
HYALO  TACTGAAACGAAGTTGCTGCTGCGATCCGACGCATGATAATTTACTTTTCCGCGTCTC-GTCGATATAAAAGCTATGGAT

     1430      1440      1450      1460      1470      1480      1490      1500
PINFES TGCCGTTGGAACGTTCTTCGATTTAACACGCCATGCACGGTGCTTCCCTTTGTAGACGCCTACGGTGCCGTTCTGAAGAG
       :: : : :  :    :::::: :: :: : :: ::::::      :::::: : :::: : :  :  :: :: :: : :
HYALO  TGTCCTGGACATCCGCTTCGACTTGACTCACCCTGCACGTAAACACCCTTT-TCGACGTCCATAGCTCCATTATGCAAAC
```

```
     1510      1520      1530      1540      1550       1560      1570      1580
PINFES CATAGCCGAACATTTGCTTCTTAATCGCTGTGAGTGCCAGCAC-TCGACTTGTCTCCGTGCACTCGAAAACTACTTCTTC
       :                                  :::  ::: : :::           :::::::  :: ::::: ::
HYALO  C----------------------------------GCCTACACATAGACA----------CACTCGAGCACAACTTCCTC

      1590       1600       1610      1620      1630        1640      1650         1660
PINFES ATT-CTGGAATGCTCGG-ATCTCAAAGTCATCTGATCCGGCAATGAGTTCATC--GCGTCCATGGCCAGTCACAT---TC
        :: ::: ::::::: : :::: ::::: : : :::: :::::  :  :::::  :::::   : :::: :::::   ::
HYALO  CTTTCTGAAATGCTCAGCATCT-AAAGT-ACCCGATCTGGCAACAAACTCATCAGGCGTC---GACCAGCCACATCCTTC

              1670                     1680      1690      1700      1710      1720
PINFES ACTGTCA-GTGCA---------------CGAACATGATCTCCAGTGACTGTCCAGAAGAGCTCACCACCGTCCCGACCGA
       :: :::  :: ::               :: ::::::::: : : :   ::::::   ::  :     : :: ::::: :
HYALO  AC-GTCGCGTACAGTCGTAGGATACAATCGGACATGATCTTCCGCGCTCGTCCAGTGTAGTCCGTTCTCATCACGACC-A

         1730      1740         1750      1760      1770      1780         1790
PINFES AGCCTTGCAGCGAGCAGTT---TCCGCCCACCACGACCATCTGAGACGGAGAGTTTACCAACGAGG---CAGTGCGTGGT
       :   :: : :: ::  ::    :::  : : ::::::::   ::   : :        ::::: :    :::: ::  :
HYALO  AATTTTACCGCCAGAGGTAAGCTCCTTCTATCACGACCACGCGATGTGCA--------CAACGTGATCTCAGTACGAAGA

     1800           1810       1820      1830              1840       1850
PINFES GCAGGTGC-----GAAGAGC-ATGGCACTCACGCCATCG--------GGAACATCCTT-ATAGAAGATGTCGG----AGT
       :  ::::      ::::: : ::::  :    :  ::::        : : ::  : : ::  :: ::   ::    : :
HYALO  GATGGTGTCGCTTGAAGATCTATGGGTCGATGGGAATCGATTGGTGTGTACCACACATCATGCAACATTCAGGGCACAAT

    1860      1870      1880        1890      1900      1910      1920      1930
PINFES TCTTCTCCACGTCGTAAGCCAGCAAGCTGG--ATTGTGTGCCCACTACGAGCGTGTCGCCCTGATCTGCGTTCTCGAACT
           :::::  : :::   ::: : :::    ::: : :: : ::   : :    ::::: :: :: : ::   : :::
HYALO  GAAGCTCCA--TGGTA---CAGGA-GCTTATCATTTTTTGACAACCT-GTGGACATCGCCATG-TC-GAGTAG-CAAAC-

      1940       1950      1960           1970      1980      1990      2000      2010
PINFES TGCCG-GCTGCCAGTGCCGTGACCTTGCGATTG-----ATCTTGAGGAAACGCACGCTACTTTGGTCGCTGTCATCTGCC
         ::: :::: :  :  :::::: : :: :  :     : ::  ::::       ::::   ::: :  :: : : : :
HYALO  --CCGTGCTGACGTT--CGTGAC-TGGCAACAGTAACAAGCTCAAGGAGGTCATAGCTATCCTGGGCAGTGAC-TTTCCG
```

```
                2020      2030      2040               2050      2060       2070
PINFES TCCGAGTT----ATCCCGAAAGGTGTTCTCGTTT--------GGCGAGTG-GATGAATACTTTGCCC-GCAGTAGTGCCG
       :  ::: :    :  : : : :  : :::   :         ::::::   : ::::    :::::  : :: :::::::
HYALO  TTTGAGCTGCGTAATCAGGATGTGGATCTTCCTGAGCTGCAAGGCGAGCCAGCTGAAA---TTGCCAAGGAGAAGTGCCG

      2080               2090      2100            2110      2120      2130      2140
PINFES CAT----GCCAAC-----GCGGGGTGGCGACC--GTCGA----ACTTTCCTACTTGTGCTACACGCTCAAGAATCGGCTG
         :    :::::      :: :::::  :     :: ::    :: :  :: ::  : : :  ::::::::   :  :
HYALO  TCTCGCGGCCAAGCAGGTGCAGGGTGCTGTATTGGTAGAGGACACGTCGCT-CTGTTTCAATGCGCTCAAGGGACTACCA

              2150              2160      2170
PINFES GTGC---AAAT------GTACTTGGA--AAGCAGGTAAC
       : ::   : ::      :: ::: ::  :: : ::: ::
HYALO  GGGCCTTACATCAAGTGGTTCTTAGAGAAAACGGGTCAC
```

PITG_4848
Waterman-Eggert score: 399; 54.9 bits; E(1) < 7.4e-11
61.7% identity (61.7% similar) in 332 nt overlap

```
            110       120          130       140       150       160       170
PINFES CACCGATACAAGGAGCACCCGAGTA---GTGTCAGCGACCCTCGAGTGAAATCCCCAAAAGGGTTCCGTGCTTTCGCCTG
       :::  ::: ::: :: :     :::   :::::    : :::::  ::       ::::    :: :  ::::: :: ::
HYALO  CACTAATAGAAG-AGGATGGACGTAAAGGTGTC----AACCTCGCCTG-------CAAA----TTTCTCGCTTTAGCGTG

     180       190       200       210       220        230       240       250
PINFES TACATCAGCCAGGTGTTCGTGCAGAGCTTTCTTCTATGTTGTGGCG-GAAGGCGCCTGGATCCTACGATGCCGCTGCAAA
        :: ::::::: : :::::::  ::::::::  ::: ::::::::: :::::  : : ::::::::::  ::  ::::::
HYALO  CACGTCAGCCACGCGTTCGTGTCGAGCTTTCAACTACGTTGTGGCGAGAAGGA-CATAGATCCTACGACCCCAGTGCAAA

      260       270        280          290       300       310       320       330
PINFES CATAGACACACGAATCACGAC-CCGG---GAAGCAAACCATTTGTCTGCAAAAGGCCCAAGTGCGGCTGTCAAGGATTTG
        :: ::: :::::    :::  ::::   :: :::::::: : :  ::::: : : :   :: :: ::: ::   : : :
HYALO  AATGGACGCACGAC---CGATACCGGATAGACGCAAACCAGTCGCATGCAAGAAGTCTGGGTACGACTGCCAGACAGTGG
```

```
          340       350       360        370        380       390          400
PINFES ACAGTCCGTGGGTTTGCAATTGCGACCATCCG-TGGGGCGCG-CACCGCCAGCATCTTG---TCGAGAAGAAGTTTGACC
       :::      ::: ::::  :: ::  ::: :: : :   :   ::: ::::  : : :    ::::: :   : ::  ::
HYALO  ACA----AGGGGCTTGC--TTACG--CATACGATCGAAAGTATCACAGCCATGAACCTACGTTCGAGCAC--GCTTACCC

     410       420
PINFES CGCTGCAAATGT
         :: :  ::::
HYALO  ACCTCCGCATGT
```

```
PITG_21013
Waterman-Eggert score: 1051;  91.9 bits; E(1) <  5.4e-21
53.9% identity (53.9% similar) in 1906 nt overlap

             40         50        60        70        80        90       100       110
PINFES CGCTGTTG-TCATCCATCCCGTGGTGTCTGCGGAATACTGCGCTGTGTGTTTCAGCGTGAATCATCTCGAGGTATTCGGC
       :: : ::: :::  ::::  :: :::: :::   :: :::: ::  :   :::::: :: ::::    : : :: ::::
HYALO  CGATCTTGGTCACGCATCGTGTAGTGT-TGCAATATGCTGCACTCAGCAATTCAGCATGGATCACTGTGGGTTACTCGGT

             120       130       140       150       160       170       180       190
PINFES ATTATGAATTTCGAACCGGCGCTGCCGAATGAGATGCCCAAGGTCCCCGATGGTCCCTCCGTCGTTGAGGTCATGATCGA
        :: ::   :: ::::   : :::: ::   ::  :   :: ::::::  : :: :  :: :::  :      : : :
HYALO  GTTTTGTCGTTTGAACATTC-CTGCTGAGGCAGGCGTTAAAAGTCCCCACTCGTTCTGCCATCGCCG------TAAGC--

             200       210       220       230       240       250            260
PINFES GAATCAAGTCCATGAGAACGTCGCACATCCGGCGCACTTCAATATTCGGCGCATCAGCAGCG-----AGAAGCA-----A
        :::  :::: :: :: ::::: ::::::::::: :::::: :  :: : ::: : : : ::     : :: :      :
HYALO  -AATTGAGTCTATTAGCACGTCTCACATCCGGCGTACTTCAGT--TCTG-GCAGCGGGAACGGACTTACAAACCTTTTGA

              270       280       290         300       310        320       330
PINFES GTCCG-GCACAAAGCCGACACCAAAGAGAATATCTTCAA--ACGTATCGGGG-TGCCTCAGCATGCGATAAAATGCATAA
        : :: ::   ::  :::   : :   :   : :: ::   : :::  :    :::::: : : :  : : :: ::: :
HYALO  CTACGTGCTAGAACGCGATTGCGAGTGGGTGAGCTACATTGAGGTACTGATCCTGCCTCTG-AAGGAACAGAACGCAAAT
```

```
       340       350       360       370       380       390       400       410
PINFES AGGATCTGTAAGGTGAGCTCGTTGTCGTGCTGTTTCTCTTGGAATAGTGAGTGCAGCGTGCGCACAAGTCGAGATGATGA
       :: :  : : : ::::::  ::  ::::::: : : ::::::: : ::: ::
HYALO  AGAAGTTATGATGTGAGCAGGTA-TCGTGCTATGT-TCTTGGCAAAGTAAG-----------------------------

       420       430       440       450       460       470       480       490
PINFES CAGCAGAGGAGCACACTTTGGTTCCAATGCGAGCGTGCTAACAAACAGTACGACTTCCAAAATCATGTCATCTTGTGAAA
              :: : : :: :       :::::::::   :: ::  : ::::: :  :  :  : : : :: :  :: : ::
HYALO  ------CGGGGTATACGT-------AATGCGAGCA--CTGACGCAAAGTACAATGTTAAGGACCGTATCGTTGTGCGGAA

       500       510         520       530       540       550       560       570
PINFES AGCCCGGTACAAGGAGTTTGT--TGAGTAGCGGGATAACAGAATGGTCTGTCACCAATCTGTCCCATGAACCCTTCGACG
       :: :   ::: : :::    :  :::  :: :::: ::  :: :: :::  : ::::: :::  : :: :: :: :
HYALO  AGTC---TACCATGAGCGGCTCGTGAAAAGTGGGAAAAGCGAGTGATCTACCGCCAATATGTTTCGTGCACTCTCCAGTA

         580          590       600       610       620       630       640       650
PINFES GTAGATCACT---TGGCGTCATGTTTGCTAGCGTCGCTACAACTTCAACAGAAAAGTCGTGGTTGTCAGTAGTAAGACAT
       :::: :: ::   :::  ::::::  :::  : :: : :   : ::::              ::::: ::     : ::
HYALO  GTAGGTCGCTCCGTGGAATCATGTCCGCTCACATCACCAGTTCCTCAA--------------TTGTCCGTGACGTGTCAA

            660        670       680       690       700       710       720       730
PINFES AGCTCGATAAA-TGGGACCACGAAGGGGGCCCACATGCCTCGATACTTGTACGCCTTGTCGCTGACCAAATCTTCCTGCA
       :: ::  : :: :::   :::::  :  ::  :   ::  :: :: :::   :: :: ::::: ::: :: :::  ::::
HYALO  AGTTCAGTGAAGTGGTGTCACGATTGCAGCGTA--AGCAACGGTATTTGGG-GCTTTTTCGCTAACCGAA-CTTG-TGCA

              740       750       760        770       780       790       800
PINFES GCGTG-TACGACCACAGTGCAATATTTCGAATCACCTT-CATCAGCAGCGGGTCGCGAGTGCGAAATACACGTGAAGCAA
       ::: : :: ::: : ::::  ::  ::   : : :::: : :: : :::: ::::: ::::: : : : ::::::  :::
HYALO  GCGGGGTATGACTA-AGTGATATGCTTGTGAGCTCCTTTCCTCCGAAGCGAGTCGC-AGTGCAATACAAACGTGATACAA

     810       820           830       840       850       860       870       880
PINFES GAGCTTCTAGACCACGGT----TCTGGCACATGAGTTCGGCATTGCGCGCGTTGTGGCTGAGGTTCACAGACAGCGCAGC
       : :::: :: :    :::    : :: : :::::: :::   ::: : ::      ::  ::::        ::: : ::
HYALO  GTGCTT-TAAA----GGTGTCATTTGACGCATGAGATCGAGTTTGTGAGCCAACATGC--AGGT--------AGCACGGC
```

```
         890        900       910       920       930       940       950       960
PINFES CAGCTCCTTC-GCCACGATATTCTGTGGGAAATTAATTACCAGTTGCATGACGATTGGGACTGCCTCTGTGTATGTGAAC
        ::: : ::: : :::  :  :::: : ::: :: ::  :::::::::  ::::::::   :: :::  ::    :: ::
HYALO  TAGC-CATTCTGTCACAGTGGTCTGCGAGAAGTTCAT--CCAGTTGCAC-ACGATTGGATGTGTCTCCCTGCTGATGGAC

          970       980       990      1000       1010      1020      1030      1040
PINFES ATTGACTTGCATCGATCGTCGATGCTGAGGTGGTAAAG-GATTCGTAGGGAAAGCGCTCGAAAAGGAGGTTTCTTCAG--
       ::   :: :  ::::   ::::::: ::  :: :: :: ::  :   :  : :::::::       :  ::: :: ::
HYALO  ATAAGCT-GAGTCGACTATCGATGCCGATATGATACAGAGACACCG-GTTACAGCGCTC-------ATCTTTTTTGAGAG

            1050       1060      1070      1080      1090      1100      1110      1120
PINFES TAGCTCCACCAGCT-TCGGGATAAGACTATTTCTCACCAGCACTTCGCGAAGCGTTGGATCGAAGGACAAGTTAAACAGC
       :::: :: : :::: ::   :  : ::: :: ::: ::: :::: :         :::::    :: : ::::  : :::
HYALO  TAGC-CCCCAAGCTCTCCCAACGACACTCTT-CTCGCCACCACTACC--------TGGAT----GGTCGAGTTGGATAGC

             1130      1140      1150       1160      1170      1180      1190
PINFES AGTTTGAGTGATGTCTGGATGGGTTTGTCGTGGTTACAA-TTGAGGTATCGCACTAGCTTTTCCACCACTGAAAGCTCCA
       :             : ::            :: : :::: :::: :: ::: : : :  :  :    ::::  : :  ::
HYALO  A-------------CGGG-----------CTGCTAACAACTTGAAGTGTCG-AGTCGGGTGGCACGGACTGCTAACAGCA

    1200      1210      1220      1230      1240           1250      1260        1270
PINFES TCATTGCGTCCTTGTTTTCCTCAAAAATGCTCAACTTCTTGAG--GAATGTG---ATAGTCAGAATT--ACCAGATCGGG
       :  :::      :: : ::    ::  :: ::  ::  : : :  : : ::    :::::  : :::  : :::     :
HYALO  TTTTTG------TGCTGTCTG--AAGGTGTTCG-CTGGTAGGGCCGCA-GTCCACATAGT--GCATTTGATCAGGGTTTG

           1280      1290                1300      1310        1320         1330
PINFES TGCTACACGATCCAACAACTT---AGT-------CAGGAAAATCACAATCTTGCGC--TTGA---CCATCTTGCGCTCGG
       :::::: :::: ::::::::    :::       ::  :: :  ::::   :: :   ::::   ::::::  ::  ::
HYALO  TGCTACTCGATTCAACAACTGTCCAGTGTCCAAGCACCAACAATACAAGGATGTGACATTGAGTCCCATCTA-CGA-CGC

      1340      1350      1360      1370      1380              1390      1400
PINFES TATGAATGTCTTCTGCCAGGTTCAGCAGCACGCTGAAGCACACACAGAA--------CAGTTGGTCTTGCTTTTTCATGC
        :  :    :::::: ::: ::::  : :: :   ::::  :::   ::        ::::    ::: :  :  : :
HYALO  CAGCA---GCTTCTGACAGATTCAAAAACA-GTCCAAGCGAACAGCTAAACTCGAGCCAGTACAGCTTCCGGTGACTTCT
```

```
   1410      1420              1430               1440      1450      1460      1470
PINFES GCTTTTTGTTGCGC-----TTCT---TCTCAC-----GGTCA----AGCTTTGCTAGCGCAAAGTCGGTATCGTCTGTTG
       :::: ::: : : :     ::::   :::  :     :::::    :::::: :  :   : : :   :    :: : ::
HYALO  GCTTCTTGCTTCTCCCGCATTCTCATTCTGCCTGTTGGGTCAGTTGAGCTTTACCGGACTACAATTTCTTGTATCCGATG

           1480         1490           1500      1510       1520             1530
PINFES GGGCCTCATGTGCGG---TGGCTTCAA-----GCATTTTTCCCTCTTCGAG-ACGTA----GCTGGTGA---CGCTTAAG
        :   ::::  :: :   : :::  ::     ::: :: :::  ::  ::: : :::    :: : : :   : : :: :
HYALO  TGCTTTCATACGCTGCATTTGCTGTAAAAGTAGCAGTTCTCCTCCT--GAGTAAGTAAAATGCAGTTAAAAGCACCTAGG

       1540      1550         1560      1570      1580      1590      1600      1610
PINFES CTCAAACTCGACGA---TCTTCAGTGTGATAGCCCCAATGCGGTAGTTAGCCAGTAGCGCATGTAACTGTAGGAAGTTGG
       ::: ::::  :::    :::: :: ::  :   : : :  :  :: : ::      : ::   : ::::   :: : ::
HYALO  CTCGAACTTTACGTCCGTCTTGAGCGTCCTGTACTCGAGCCACTAATGAG------GGGCCGTTGACTGGCTGA-GCTGC

           1620        1630         1640      1650      1660        1670      1680
PINFES AGAAGC-ACCAGAAGA--TACGCATCATG---ATGAGCGAAAAGTCGTAGCTTTTCTTGT--AGTCGTCATTCAGAACAC
       ::   : :::   ::   :::  :: : :   :: :: ::: : ::: ::   : :: :   ::::: :    :: ::::
HYALO  AGCTCCTACCTTTAGCCTTACTAATGAGGTGAATCAGTGAACACTCG-AG---TCCTAGCCAAGTCGCC---TAGTACAC

        1690      1700      1710             1720      1730        1740      1750
PINFES GCGACAACAGGCTCATGAGTGATTCAT-TC------TGCACGAGGACTTCAATGTTG--CCTGCATACTCTGCCAGTTCA
        :         ::::::   : : ::: ::      :::: : : ::  ::  :::   :  :::        ::: :::
HYALO  AC---------CTCATG---GCTCCATCTCAGCTGGTGCA-GCGTACCGCAGCGTTTGTCAAGCAG-------CAGCTCA

       1760      1770       1780      1790      1800      1810      1820
PINFES AGGATCATCTTGGCGCCCTGCA-GCTTGGACTCGACATCGTCATCGTAAAGTTTCTCCATGTAGGC
       ::   :: :  : :::   ::: : ::::   : :::: :       :: :: :::  : :: :::
HYALO  AGCC-CAAC--GACGCGTCGCACGATTGGGG-CCACATTG-------AACGTGTCTGGACGTTGGC
```

PITG_2721

Waterman-Eggert score: 268; 33.6 bits; E(1) < 0.0006

49.7% identity (49.7% similar) in 1397 nt overlap

```
                20        30        40        50        60         70        80
PINFES GTGCT-ATTGGTGCCGCTTGTTCTCGTAGCGGGTGTTCGCTGCCAGTCTGCTCCC-AGGAAAGTCGTGTACATACAGCCT
       ::::: ::: :::  :    ::: :::    :::  :::  :::    :: :: : :   :::   ::  :::   :  :
HYALO  GTGCTCATTTGTGTAGGGAATTCGCGTC---GGTTATCGACGCCGCAATGTTCACGATCTAAGC--TGCCCATCGTGGTT

       90         100       110       120       130          140       150       160
PINFES TCATTCT--TTTTGGTGACATTACCGTCACGGAACACGAGATCAAAGC---CTCCCATATGCTCCTGAACGACCCCCTCG
       :::  :   : ::   ::   : ::  ::: : :::  :: :::: ::   : ::  :: :::       :  ::: :::
HYALO  TCAAACGACTCTTCAGGA---TTCCAGCACAGTACAAAAG-TCAACGCTATCACCTCTAGGCT-------GGTCCCTTCG

           170        180       190       200       210        220
PINFES GGTTTCC-TGCTCTCGAGGTCGATTATATCAAGCATATCGTTCAGCATTCCGC-ACTTG-----AGCTG--------ATA
           : : :: ::   :: ::::: :: :: ::::: :      : :: :::  :::::     ::::         : :
HYALO  TTAGTACGTGTTCGTCAG-TCGATAATGTCGAGCATTT------GGATCCCGTGACTTGTTGGTAGCTAGCGACCGCACA

      230       240             250        260          270
PINFES ATCTGACGAGGTGT------TAGCTGAAAGGGAAG-GCGAA---GCGTTCACCTCCAAG-------------------AG
       : ::::   : :::      :: :::  :  :::  : :::   ::::: :::: : :                    ::
HYALO  AGCTGAACTGTTGTCGAAGATATCTGGGACAGAACTGAGAAACAGCGTT-ACCTACGATGGTAAAATATGTGCTGAGCAG

     280       290       300       310        320       330       340
PINFES CCACACCTTGAGGTTTTCATCGATCATAATGTCGT-ATCCGTACAGCTCGAAGCAGTGTTTAT--CGTTCATG-------
       : ::: :::  : :: :  : ::::::   : ::: ::  : ::::: :  :::::: ::: :  :: ::: :
HYALO  CTACAGCTTTGGATTCTGTT-GATCATGCGG-CGTCAT--GAACAGCCCTGAGCAGTATTTGTTTCGATCACGGCGACTG

                                                350               360         370
PINFES -------------------------------------------ATGATCTTT--------TCGAC--GCTGAGCAAAGAC
                                                  ::::   ::        :: ::  ::  : :::::
HYALO  GTATAAACTGGATGAGAAGTGTGATGCGGAATAACCAGCATACATGACAATTGTGATCAATCTACTTGCCCAACAAAGGA

        380       390       400       410       420       430       440       450
PINFES TTGATGATCACCAGCTGAATCTCGTAGAAAAGCTTGTTAATTCGTTCAGGTCCATGCTTGGACATCATGTACAGCTTCAA
       :::::  :::: :: :::    : :  : : :   ::   :: : ::: : :  :: : ::::: :::: ::::::::::
HYALO  TTGATCGTCACGAGTTGAGCTCCATCTAGAGGTCCGTCGGTTAGATCAAGGCGGTGTTCGGACAGCATGAACAGCTTCAA
```

```
        460       470       480       490                  500             510
PINFES GTTGCGCAGATCCCATTTTCCACCGTGCTGTTTGT----------TGTAGTT-CTCA-GACGT-----TTTCTGG-----
       :: :  :::   :: :::  :::: ::::  : ::          :: ::   : :: :: :      ::: :::
HYALO  GTCGTACAGTCTCCGTTTCTCACCATGCTAGTAGTGAGACAGGGATGCAGCGGCACATGATGGGTAAATTTGTGGTGACC

                   520                 530             540             550         560
PINFES ---------ACAGCAACATTC----------GTGAG------GTGGATGAAAG------AATTGGCGAT--GTCGCTGTG
                :::  : ::::           : :::      : :: : :: :      ::: : : ::  :::::: ::
HYALO  GTACGGGTCACACAATCATTATCTATACAGAGGGAGCAAGCAGAGGGTCAATGTTGGTCAATCGTCCATCAGTCGCTTTG

             570       580                590       600       610                620
PINFES ATTGTTCGAGTATCGCGAA--------TTGGTGA-AGC-GCGCGAATCCACCGCGGAAA---AGGTAG------ATTTCA
          :::  :  : :  ::        :: :: : ::: :: : :: : :: ::  :::   ::: ::      ::  ::
HYALO  CAGGTTTAAA-AGCTTGATGCAGGTCTTTCGTCACAGCTGCACAAAGC-ACAGCACAAATACAGGGAGTAAAGAATAACA

            630       640       650         660       670       680       690       700
PINFES AGTGGACAGAAGGACGGCACGAGTGCGTATAG--GCGCAGATCAAATTTCTTTCCGCCGACAAGGTACGGGCTTGAGATG
       ::  ::  ::: : : :  ::    : :::::  ::::        :  :: :::::::    : ::  ::::   :  :
HYALO  AGATGATCGAATGTCAGT-CGCTAACATATAGTAGCGC--------TACCTATCCGCCGTTCCGATATTGGCTCTTGCCG

              710       720       730       740       750       760       770       780
PINFES TAGCGCTGCACGACGTACGTCTCTACCTGTTGATTTTCGGGCCGCCAGCGGAAATCCGTGCGCCAGTCACTGATTTGGCT
       :    :  : : :::   :: ::::   :::::   ::    : : : :: :    : ::::   ::::   :::: :
HYALO  T----CCACCCCACG---GTATCTA---GTTGA---TCACAACACAAACGCA----CTTGCG---GTCA---ATTTAGTA

                  790       800       810       820       830       840       850
PINFES CAGCT----TCGTAAACAGGAAGATGCCTTTACCTTGTGCTTTTCCAATGGGCTTCATGATCCAAACGCCCTGGTTGCGC
       ::       ::::   : :  ::: : ::   :  :::      :::::   ::::: ::  ::  :::   :  :::
HYALO  CAAATACCGTCGTTCTC-GATTGATTCGTTCGGCCCGTGACGCAGCAATGCATTTCAT-ATA-AATTGCCAAAG--GCGA

        860              870              880        890             900        910
PINFES TTGAACTCTT-------CGACA-------AAAATGGCATACT-CGCCCGGTA--GAA----TGTA-CGTGGCAGGAAA--
       : : : ::::       ::::        ::::   :::: : :: : :: :  :::    :: : ::: :: : ::
HYALO  TAGCATTCTTTCAGATCCGACGGAATCTCAAAAGCTCATATTGCGTCAGGCATCGAACACATGAAGCGTCGCTGAAATAC
```

```
             920       930              940       950         960         970       980
PINFES GAAATCGTACGAGCCCAGCGCCGA---GTC----AAGCTCACTTAATGTTGCAT--CA-TCC-GACCTCGCCTTCTTGTC
       : : :  ::::    ::   : :    :::    :::::: ::  :: :: :::  :: ::: :: : : :: :     :
HYALO  GCATTAATACGCCTTCACTTCTGGACTGTCTACAAAGCTC-CT--ATATTCCATTTCACTCCAGAAC-CACCAT-----C

              990      1000      1010      1020      1030      1040       1050      1060
PINFES CATTTGTCGCTTTGCTCGCTTCAGGTTTTTAATGAGCAGATCTTTCCGACACAACTCTCT-TCCATTTCGATAATGATTC
       ::::  :::  :  ::     ::   : : : : : :: :    : ::::::::   : : :  : :: :: :      :
HYALO  CATTGCTCG--TGACTGAAAACAAACTGTCAGTCA-CACA----TGCGACACAATGTTATGTGTACTT-GACAC-----C

              1070      1080        1090      1100          1110               1120
PINFES ACCCGTTGGCTGTTCTCGAG--GTGCACAGTATCCAAAAGCTCG----TAGATATACTCG-----CGG----TCAGCCCA
       :::: :   : ::::::: :  : :: : : : :   :: ::::    : :: : : :::     :::    ::  ::
HYALO  ACCCATAAAC-GTTCTCGGGTCGGGCGCTGCACCACTAAACTCGGCATTCGAGA-AGTCGAAACCCGGATTCTCTTCCTG

         1130        1140      1150      1160
PINFES GTAAAAGTCCCAG--TCATTCTCCGTTGTCTCCTTCC
       : ::: :  ::::  ::::: :: :::   : ::: :
HYALO  G-AAACGCTCCAGCATCATTTTCTGTTTCGTGCTTTC
```

PITG_0942
Waterman-Eggert score: 492; 53.8 bits; E(1) < 1.9e-10
62.0% identity (62.0% similar) in 371 nt overlap

```
        30        40        50        60        70        80        90       100
PINFES TGGAGCAGTATGCCGATTGGTTGGATAATTTGGAGAAGACGGATCCGGAAGGCTACGAGCAGTTTATTGCCACAATGCAG
       ::  ::  ::: :  : ::: :: :  : :::::: : :::::::: ::::: ::  : ::   : :::: ::  :::::
HYALO  TGCTGCGCTATTC--AGTGGCTGAACGAGTTGGAGCAAACGGATCCAGAAGGTTATCAACACGCTCTTGCGACGTTGCAG

       110       120       130       140       150       160       170       180
PINFES AGTCAACTGCAGGCCGCAGGACTGGGACCAGAGAGCTCCGCTGGTGCACCACAAGGTGACGCAGATGTAGCATTGCTCGA
       :::::::::::: :  : :::   :: : : :   :    :: :   :::: :::::   ::: ::  : :  ::::  :
HYALO  AGTCAACTGCAGTCTACTGGATCAGGGCTAAATGACGGTCCTAGAATACCAAAAGGTTGAGCAAATCGATCGCTGCTGCA
```

```
      190       200       210        220       230         240        250       260
PINFES CATGCTAAAGAGCTCAGCAGGTGGA-CGAGGAGGGGCCGCCGACACCATC--TCGTCT-GATTTCAAGCCGCGGTTTCCT
        ::::: :  :: : :::   :: : : :     :   :  :: :   :   : :::: :: :::::::: :::::: ::
HYALO  TATGCTCAGTAGAT-AGCCCTTGCAGCTATTGATGATTGATGATATTTTGGGTTGTCTAGAGTTCAAGCCTCGGTTTTCT

          270       280       290       300       310       320       330       340
PINFES GGTGACAAGATTATGGGGAATGATGGCTTGAAAGCCACTCCAGAGGGGGTGTACATTGATGTGAAGCCTGGCTTCGTCAT
       :::::::::::::::: : :  : ::::::::::::::  : :: :::::    ::   ::: :: : :::  :  :::
HYALO  GGTGACAAGATTATGGAGGAGAAGGGCTTGAAAGCCACAGCGGAAGGGGT----ATGAGTGTTAA-CGTGGAGTGCTCAA

          350       360       370         380       390
PINFES GAAGACAACCGATGTTAAGAACAAAAAGAAG-GTT--TTTGTAAATGTAGT
       : ::  ::  :  ::     ::: : ::  : : :  ::::::  ::: ::
HYALO  GCAGTAAA--GGGGTCGTCGACAGAGAGCTGAGCTAGTTTGTA--TGTTGT
```

PITG_12763
Waterman-Eggert score: 472; 50.8 bits; E(1) < 3.1e-09
51.6% identity (51.6% similar) in 1430 nt overlap

```
              10         20         30         40        50        60        70
PINFES TAAGAGCGCGAG-TGTTTGAGT-GCTGCTGCC-TCCGGATTGAATTCTGGAGTCTCTATAAACTGCAGAAAGAGGTTTGT
       : : ::: :::  :::  :  : : :: :::: :: ::::   :   : : :::::  :::: :  :: ::: : ::
HYALO  TGAAAGCACGAAATGTGCGGATAGGTG-TGCCGTCTGGATCCGACCATAGTGTCTCGCTAAATTAAAGCAAGCGCTTCCA

       80        90       100       110       120       130       140       150
PINFES GAGGCGTTTGCGTAGCCCCTCAAGACTCTTCTCCTTCCGTCCACGACTGAGCATATCCGCCTCACGGTCAATAAGCTCCA
        :  : ::::: :: : ::::: :::: :::::: : :: :  :  ::  :  : ::: :   : :::: :: :: ::
HYALO  -ACCCTTTTGCATAACGCCTCACGACTTTTCTCCGTGCGGCTGCAGCTCGGTGTGTCCACTCTAAGGTCGATGAG-TCGG

      160       170       180       190       200       210       220       230
PINFES CTAGCTCTCGAGTGAGAGCACAGTCGAACTCTTGCACTGTCCACTTGATATGCAGCAACATGTCCAAGCGTTCGTCCACT
       :  : : : ::     :  : ::: :             ::  ::: : :::  ::: :::::::::: :   :: ::
HYALO  C--GTTTTGGA-----AATAAAGTGG-------------TCAGCTTAACATGAGGCAGCATGTCCAAGAG---GTACAAC
```

```
     240    250     260      270     280    290     300    310
PINFES GAACTGAGTGGTGTACGTAGACCAT---GGTACAGATCCGTGAGCTCTTTGGCGAACAGTGAACGGTGTGTGCACCTC
        :  : :: ::::: :   ::::::   ::   :  : : ::::::: ::  :  ::::::: :: :: : :::::
HYALO  TACATCAGCGGTGTCCACGGACCATATTGGCCAATCTTCATGAGCTCCTTCACCTGAACAGT-AAGGGCGGGTGCAAGCT

        320     330     340     350     360      370     380    390
PINFES TTTTACACCTCCGTCACTCATCTCCCACTGCTTCGGACGTGCCATAAACTG-CAGCATGGCTTCAATACGTTGCTTACGG
       :::::     ::::: :: :                   :: :: ::::: : :  ::   :::         : ::::::::
HYALO  TTTTATTATACCGTC-CTTA---------------GATGTACCATAGATTTACAATTTGG---------GCTGCTTACGC

         400     410      420     430     440     450     460     470
PINFES TTGGCCTCTGTTGCCGTTAACTTG-AGTCTGTCAATCGTCTGAAGCAGTTTGGTCTCTTTGGCCAGAATGGCGTCAAGAG
       ::: : :: :: ::: ::: :::: : ::::  :  : ::  ::  : ::  :::   :::  :       : ::: : :
HYALO  TTG-CATCCGT-GCCCTTAGCTTGGATTCTGAAAGACCTC--AAATAATTCAGTC---TTGCTC-------CATCACGTG

         480     490     500     510     520     530       540
PINFES CGTCCTGTCTCTCGGACGGATCCAGTTCTCGTTGCTCACGAACTTTCCGACATTCAAC-CTG--TCGCCATTTTTCG---
       :   ::: :  ::   : ::::::  :: :  :: :     :: :: :: ::  :::: :::  : : ::  : : :
HYALO  CACGCTGCCATTCACCCAGATCCATCTCGCTCTGTTTGGACACCTTGCGGCA--CAACTCTGGATTGGCACCTCTGGCTG

        550     560     570     580     590          600     610
PINFES -AGTTCATTGTAGAGAATCTCAAAGTCACTCACCGTGTTTGGGTGCAT-GCG-----ACGGTTGACTTCACGACGT-TGA
        :  :: :  : :    : :: :   :  :: :  :: ::  :   :: :::     :::     :: ::  :::  : :
HYALO  GAAATCCTATTCGCCTCTGTCGACCGCGATCGCAATGCTTATGAAAATTGCGTTATTACGCCGAGCTCCATTACGCGTCA

    620     630     640     650     660     670     680     690
PINFES CGACTCACGTCATCAGCGTCGTTCTCTTGGGCTTCGATCTCTGCGAGTGTACTCACACGACGACGCTGTTCACGTATACT
       : :::    : :  ::  ::  :: : : : : :: :  ::     ::  :  :: ::  ::  :  :: :: :   ::
HYALO  CAACTGCTATGAAAAGA-TC--TCACATAGACATCCAGATC-----GTACAACCAAACATCG--GTCGTGCAAGGTGAC-

    700     710     720          730     740     750     760     770
PINFES AAAGACGTAAAGTCGAGCGCGAAACCCGC-----GCCACAGACGCTGCAAGAGAAGCACAGAGTCTTCTCGTAGACTGTC
        : :::   : : :: : :    :  ::     ::::  :::: :: :  : :::  ::  : ::  : :: :     :
HYALO  -ATGAC----AATTGATCACTCGTCTGGCCTTTGGCCATT-ACGCAGCTATGGTAGCTGAGTCTATTGCCTTACAAAAGC
```

```
          780             790         800       810              820       830
PINFES TAGTTGGTA------CGAATCAAAG--TAAGGA-TGTGCAGTTACG-------TGGCGATCGAAGGACTCGTCTAGCTTA
        :  : : :      ::   : : :  :::::: ::  ::: ::::       ::::: ::::  :  ::: : :::  :
HYALO  AACATAGCAAATACCCGGCCCGAGGCGTAAGGAATGCACAGCTACGGGTTTTCTGGCG-TCGATAGGGTCGAC-AGCCCA

      840          850       860       870       880       890       900       910
PINFES AGATCCCG---TCGAGACATTTGAGTGCCGGACTCCCGGTGGGTCTGCAAGGAGCGCGTCACGAGTTGCTGTGTTTGG-G
       : : :      ::: :  :::: : :   :::   :   :: :: ::::    :     ::: :  ::: :  :::   :
HYALO  ATACCAAAAGCTCG-GCAATTTCATTCATGGAAGAC---TGTGT-TGCA----GTTGTACACTATGTGCAGCATTTTATG

          920        930       940       950       960       970       980       990
PINFES TCTCGC-GGTGGAAACGTCCCGGACCCATATTGTGACGCGCTCGTGATAAAACAGTCTGAGTGGAGGCGTGATGAAATTG
       : :::: :::: ::     : : : :  ::  : :  ::: :: ::   : : : : : :  ::  ::::     :: :: :
HYALO  TGTCGCTGGTGCAA----GCAGTAGCTCTAAGGCGCGGCGATCTTGCAGACATA-TGTCGGTAAAGGCAGCTGGATATCG

          1000         1010      1020      1030      1040        1050             1060
PINFES TTGTAGCGTTTTACG---GTGTTTGAAGCCTCCGAGGTACAATTTGTGGCCTGT---GAACTTGTC--AAT-----CTCA
        :  :  :   :: :   : :   :  :: :: ::  :::  : :: : : :     ::::::  :  :::     ::
HYALO  CTAAAAAGAGGTATGCAAGCGAAGGCGGCGTC-GACTTACCTTCTGCGTCGTTCAAAGAACTTTACTGAATGGCGACTAC

             1070      1080      1090           1100         1110      1120      1130
PINFES ACGGTTAAAATACTGGTACTTTCGGTATCC-----TCATTGTAAACCTGGA---CTTGTATCCGGTCTGGCATGACGTAG
       :::: :    ::    ::: :::    :::     : : :: :::: :: :   :   ::    : : : :: : ::::
HYALO  ACGGATTTGCTA----TACATTC---TTCCATTGGTTACTGCAAACATGAAAGCCCACTAAGTTGGCAGTCAAG-CGTAA

           1140      1150      1160      1170      1180      1190      1200
PINFES CTGCTTGTGGTGGCTGGTCTGGCGACGTAAGCTTCGAGAGTGCATTCCGAGTTGCCATTAGGTAATCCGT----ACG---
         : :::   : : ::    : ::: ::::: : ::     :::   ::  :: :   ::: : :::  :    :::
HYALO  GAG-TTGGTATCGTTGAG--GTCGAAGTAAGGTGCGGCTTGGCACAACGTTTTCCTCGTAGATGATCTATCTGAACGAAG

                         1210      1220      1230      1240         1250        1260
PINFES -----------------CTGAAAGCGGTAGATTTTCCTCCTTTTGATCTGCATCGCTAA---GAC--CATGGAGATCGGG
                        :: : :: :::: :::: :   :  :::::: : :   ::::   : :  :: : :  :::
HYALO  TCGAGTAGATATGAGTACTAATAG-GGTATATTTACGCACACTTGATC-GAA---CTAATCTGGCGTCACGCACTTCGTA
```

```
          1270      1280           1290          1300      1310      1320
PINFES GAAGCTCACAAGCTCAGAAG---TGAGC--TGT---AAGTCGGCGCACAGCATCTTCCGTAGCGTTCCGA
          : ::: : : ::  :::   :: ::  :::   ::::::: ::: ::::  :   ::::::::::::
HYALO  CC-GATCAAATGGTCTTAAGATATGCGCATTGTGATAAGTCGGTGCAGAGCAGTTCGTGTAGCGTTCCGA
```

PITG_11526
Waterman-Eggert score: 630; 53.6 bits; E(1) < 4.5e-10
51.9% identity (51.9% similar) in 1191 nt overlap

```
            10          20        30        40        50        60        70        80
PINFES TGCGCCGTCCTTGT-GT-TGAGGCTGCTTGGCCAGCACCGTACGCAGCAACTCCGTCATAGCGCAGTCTGTCCGCTCCTC
       ::: ::::  :::: :: :::  : ::  ::::  ::: :  :  :     ::::::::: :: :::::::: : :: ::
HYALO  TGCACCGT--TTGTTGTCTGATACGGCGGGGCCGACACTGGGCAAATAGGTTCCGTCATAACGAAGTCTGTCAGGTCTTC

              90        100       110       120       130       140       150
PINFES CCAGCCTTGCTCCACGGCGCCGCTGTCAGCCGTCTCGTTCACGACAGGAGAAAGATAGGCTTCTAGCAG----CCGTGCG
        :  : ::: :        ::   ::: : ::: :::       :::: ::   :   : :: : :: ::    : : :::
HYALO  ACTACGTTGTTT------GC--ATGTGACCCG-CTC-------ACAGCAGCTGGCGTGTCTACAAGAAGTGAGCTGAGCG

       160       170       180       190       200       210       220       230
PINFES TCTTCATCACTGAGATGGAACCGGAACTTAATGAGCATCAGGATGAGCGACACGCAGCACGCCAAGCGGTTGGCTGCACG
          ::::  :  ::  : : : : :  :: :::  :::::::::::::::: :: :  : : : :  ::::  :: ::
HYALO  CTGTCATGGCCAAGCAG-AGCTGAAGGTTGATGGACATCAGGATGAGCGACACCCATCGTGTCTATCCATTGGTAGCGCG

       240       250       260       270       280       290       300       310
PINFES GTTCAGCTTGGCTTCCACCGCCTCCATGGCGTGCGACAGCGAAATGAGTTGGTCGTACGTGCCGTGTAGCAGCACGTCCA
        :  ::: :: : :   :         : ::: ::::      ::::::   ::::: :::: :  :  : :: :::: :
HYALO  TTGAAGCGTGACGTGTGC---------GACGTACGACGATACGATGAGTGAATCGTAGGTGCAGCATTACCGCGCGTCAA

       320       330       340       350       360       370       380       390
PINFES GGAAGTTGACAGCGGCGGGGCTGCGATCCTTGTAGCGCACCAATAGCCGCTTCTGGATCACACGGAACTGGTGCGCTCGG
       :      :::::  :        : ::  ::: :       ::: ::  ::: ::  ::::  :    :::: :   ::::
HYALO  GA-----GACAGATGA-------CCATTGTTGGACTATGGCAAAAGG-GCTGCTTAATCATGCAACGCTGGAGTTGTCGG
```

```
         400       410       420       430       440       450       460       470
PINFES TC--GTTCAGCTGCGCTGCGAGCTCGAGTTTTTCCTTCCGAAGCACGAAGTGCGCGTCAATAGCGCCGAAGTAATCAGCT
       ::  : ::::::  :::::  ::::::   ::  :::::  ::  : ::::::::::::::: : ::  : :: :  :
HYALO  TCCCGATCAGCTAGGCTGCATGCTCGATGATT--CTTCCATAGAGCAAAGTGCGCGTCAATATCACCTGAATAGTT-GAA

         480       490       500                     510       520       530
PINFES AGCGGCAGTGGCTCCTGGTAGAACACCT---------------CGAGA--GCCGTGGCAGTTCCAGCGTCGCTTCCTTTG
       :: :   :::::  :::: : ::: :::               :::::  :   :: :: :: :::: :  :  : ::
HYALO  AG-GTTTGTGGCCACTGGCAAAACCCCTGTGTGCGGGCTTGAACGAGACAGAATTGTCACTTACAGCCTAAC--CATTCA

      540       550       560       570       580       590          600         610
PINFES GCCTCTGTGTGCAGTTCTTGTCCGTCGAAGAATGCACGAAGTCGTGCCACTAGTTCC---TGCGA--CACCATCCAGAGC
       :: ::::         :: :::   :::  :: ::: : ::      ::::: : ::   :: ::  ::: :: ::    :
HYALO  GCTTCTG---------CTCGTCAAACGACAAAGGCAAGGAG------CACTACTACCCATTGTGAATCACAATGCACTCC

           620       630       640       650       660       670       680
PINFES GCCTCGAGCTCGTTAGACTGGATACGATAGCGTCCGGCATTCTTCGATACGAGGATTGTCGCCTCGACGC----CGTTAT
          ::   :::   : :::::: :  :: :           :::: ::      : ::::: :: ::  :::    : :: :
HYALO  CTATC---CTCTGCA-ACTGGA-ATCATTG----------TCTTAGAC----GCATTGTTGCTTC--CGCATCACTTTCT

     690       700       710       720       730       740       750       760
PINFES AGTACTGAAAACTCAGTACATTTGTGCCGGAGCCTCCTGTCACTACCTGCTTCGCCCACTCGGGCGTGCAACTTGGCTGA
        :  : :: : :: :    : ::::    :   :::: :  :: :::   :: ::  :::: :  :: :: ::
HYALO  -GACCAGAGAGCTGA----ACTTGTTTTAGGTTCTCCCGCTACCACCGAATTTGCGTACTCTGATGTCCACCT-------

     770       780       790       800       810       820       830       840
PINFES TGGAGCATGTCTTCAAACAAACTAGCAAGTTCAAGAGGCTCCTGGTTTGTCTCGAGAGTAAACTTAAAGGTACTGGACTT
          ::  :::    :::::          ::: :  :: :   :: :: ::: :     :   :::::::    :: :  :
HYALO  ---AGTTTGT----AAACAT--------GTTGACCAGTCATTTGCTT-GTCCCCTACCTG--CTTAAAGTG--TGAATGT

     850       860       870       880       890       900       910       920
PINFES GACGGGAGGGACGAGGCGACATGCCATGCACATGGGGAGCTTCACAACGCACTGCGAGACCCTCGGCTGGCCTGT-CTCG
        : :: :           :: ::: ::::  :::      :::                        :::::::: : ::
HYALO  AATGGTAAA---------ACGTGCAATGC--ATG------TTC------------------------TGGCCTGTACACG
```

```
      930       940       950       960       970       980       990      1000
PINFES AGTGTGTAAGACGCTGAGATTGTAACGGAGAGCGACGTAGGCATGGCGTCGGAACTTGGACGTAAGACGACTGGGATAAT
        :   :   :::: ::   :: :::   :: ::: : :: :  :::::::::: :  :: :
HYALO  ACACT---AGAC-CT---ATAGTA---GAAAGCTAAGTGGATATGGCGTCGGGAGATGAA--------------------

     1010      1020      1030      1040      1050         1060      1070      1080
PINFES AAGCGGCGTCGATGCCTTGCCGTCGATACTCCCGATCACGAA--CGAGGAGTCACTAGCCACGACATTCTCGGGTGTCGA
              :::  :::  : :: :: : : :::::::  :  :  ::::   ::  :  ::::: ::::: :  :    ::
HYALO  -------GTCATTGCAGTACCATCAAAATTCCCGATTTCATATTCGAGA--TCGATTACCACGTCATTCGCCAGCCCTGA

       1090      1100              1110       1120      1130      1140
PINFES CACCGACAGCGTCACATTGG-------AGACACGC-GTTGCTCCAGTGTACGTGACGTACACGCGCATTGT
        :     : ::   : ::::       ::::::   :: :: :: :: ::     :::::  ::::::: :
HYALO  AA-----AACGGGCCGTTGGTAACCCCAGACACTAAGTAGCACCGGT-TATTCCGCGTACGGGCGCATTAT
```

PITG_2021
Waterman-Eggert score: 494; 112.8 bits; E(1) < 8.8e-27
53.5% identity (53.5% similar) in 1597 nt overlap

```
      3130      3140      3150      3160      3170      3180      3190         3200
PINFES TGGAGCATGAAGGCAACTACAAGGAAGCAGAACACCACTTTGCTGAAGCTGGAGAGTGGCAAGC---TGCTGTCAACATG
       : ::  : ::::: :   ::::  :: :  :::: :: :    :: :::::: ::::::   ::   ::: :: : ::::
HYALO  TCGAAGACGAAGGTAGTAACAAATAAACTAAACATCATTAAAATGGAGCTGGTGAGTGGACGGCACCTGCAGTTATCATG

         3210      3220      3230      3240         3250        3260         3270
PINFES TACCGGACGAACGATATGTGGGATGAAGCGATTCGTGTT---GCCAAGTTCCACGGC--GGGATC---AACGCCTCGAAG
       ::::: :: : ::: : ::  :   : :: : :     ::   : :: :: :    ::  ::: :    :::     :::
HYALO  TACCGAACCAGCGAAACGTCTGTCGTAGTGCTGACATTTCAAGGCATGTGCTTGTGCATGGGCTTTGGAACATGAGGAAA

       3280         3290      3300      3310      3320      3330      3340
PINFES CGTGTG-GC--TTATGCTTGGGCCATGGATCTCGGAGGTGAACAAGGCGCTAAGCTGCTCACTCGTC---------TTGG
         :: : ::  ::::  : ::  ::: :: : ::: : :  ::   :   :   :::  :: ::  :         : ::
HYALO  TCTGGGTGCCATTATCGTAGGCTCATTGAAC-CGGCGATCTACTGTGTCATTGTCTGAGCATTCTCCGAATAACGTTCGG
```

```
         3350      3360              3370        3380      3390      3400      3410
PINFES ACTCATCGAGCCGGCAATAG--------ACTACGCTATTGA--AAGTGGAGCGTTCGAGCACGCCTTCGAGCTTGCACGC
        :   :::  ::  :  :::        ::      :  ::  :::::: :: :  :::::::::: : : ::::: :
HYALO  TCCGTTCGCACCACCTTTAGTTGCAGGCACGGGCAAAAAGAGCAAGTGGCGCTTGTGAGCACGCCTCCCAACTTGCGCA-

         3420        3430           3440                      3450           3460
PINFES AATTGTGCCACCAAA--AAGCTACCGG-----AGGTGCATC----------------TCA-----AACATGCGCTGTTTT
       :: :::::  ::: :  ::::: :        :: : ::::                :::     : ::: :  :: :
HYALO  AAGTGTGCT-CCATACGAAGCTGCATATATATAGATTCATCATTAGCATATATAGATTCATCATTAGCATACCTTGATCA

       3470      3480      3490      3500      3510      3520      3530      3540
PINFES TCGAGGATGAGGAACGCTTTAAGGAAGCAGAGGAAGAGTTCATCAAGGCTGGGAAACCTCGTGAAGCGCTGGACATGTAT
       : :: :  :  ::   :: :: ::  :  :      : :: :  ::: : :  :::   ::   ::: :::: : :::::
HYALO  TGGACGGCGGAGAGGACTGTACGGGGGGCGGCTGGAAATTAAATAAGTCCGAAAAATGACG--GAGCCCTGGGCGTGTAT

       3550      3560      3570      3580      3590      3600      3610      3620
PINFES GTGCACCAACAGGACTGGCAAAACGCCATGCGCGTCGCGGAAAGCGCGGACCCGGCGTCTGTGGCTGACGTGTTCATCGC
        ::::    ::: :: :: ::  : :  ::   ::: : : :::::: ::  :  :      ::  :: :: :: :: ::
HYALO  ATGCAT---CAGTACAGGTAATTC-CATTGTATGTCACCGGAAGCGCCGATTCTACACAA--GGTCGATGTATT-ATAGC

       3630      3640      3650      3660      3670      3680      3690      3700
PINFES TCAAGCGAGACTCTGGATTGAGCGCAAGGAACATCAACGTGCTGAAGGCTTCTTCCTCAGTGCCGGGAAACCAGAGCTGG
       :: :::: :::   ::::   :: :::::  : :     ::  ::::: :  ::: ::::::: :: :: :
HYALO  TCGAGCGTGACAGCGGATCAGGCACAAGGTGCGTGTGTTTG--GAAGG-TCTTTCTTCAGTGCAGGCAAGC---------

       3710      3720      3730      3740      3750      3760      3770      3780
PINFES CTCTTGCCGCGTATCTGGAGGCTGCGATGTGGGCTGACGCAGTGCGAATTGCTAAGCGGCATCTTCCTCATAAGCTCATG
               :: ::::::    :  ::::       ::  :: ::: :::: :::  :: ::   : : :  : :    :
HYALO  --------GC-TATCTG----CCCCGATA------GATACAATGC-AATTACTACACG-CACAATTCGCCGACG----TC

       3790      3800      3810      3820       3830      3840       3850      3860
PINFES GAGGTGAATATGGCTCATCAACGCGCTATCTTCTCT-GGAGGACCGAAGAAGAAAGAA-GAGCTCATGGAGGCGTGTGAG
       ::    :::::   : :: : ::: : ::::: : : :: :  : :::: : ::: :: ::::::::   : ::
HYALO  GAC---AATAT---TTATAAGCGCACCATCTTTTATAGGCGTGCTGAAGGACAAACAATGAGCTCATCTTGACG------
```

```
         3870      3880      3890      3900      3910      3920      3930      3940
PINFES ATGTGGGTGGCCTCGCAGCAGTATGTTCAGGCAATCGACGCGTATTTGTCGATTTCTATTGACCAGATCAGCGACCTTGG
       ::::::::  : :: ::: ::  :::  : : :     :::: :   ::::      : :: :: : :::  ::::: ::
HYALO  ATGTGGGTATCTTC-CAGTAGCTTGTCTAAGAAC----CGCGCACCCGTCG------AATGTCC-GTTCACTGACCTCGG

         3950      3960      3970       3980      3990      4000      4010      4020
PINFES AGAGCTGGAAGAGCTCTGGGCGAAGGCCATCGA-GCTGTGCGCTAAGCACGACCCGAGTCGCTACAAGTCGATCGTGGAG
        :::: : ::: : :    :: ::::: :   : :::::  :  ::  :  :          :: ::::   : :: ::
HYALO  GGAGC-GCAAGTGATAGTTGCAAAGGCTACAAAAGCTGTATG--AAATATAA----------TATAAGTTCTTTGTCGAA

          4030      4040      4050      4060        4070         4080      4090
PINFES GAGGTGGCGTCACGCCTCCTTGGAATGTCTCGCTTCGACTCCG--CTGCTGAGCA---TTTCCAGTCTATTGACAAGATG
        : ::::: :  :::  : ::   : : ::::: ::::  : :  : :::  : :   :::: : : ::  ::: :   :
HYALO  AACGTGGCTT--CGCAACTTTCACACGCCTCGCCTCGATACGGTGCCGCTCGGTAATTTTTCAAATGTACGGACCAAGAG

     4100      4110      4120      4130      4140      4150      4160      4170
PINFES AACGAGGCCCTGGATTGCTATCTGCGCGTCAATAACTGGGCTGCCGCTCAGAAGCTGTGCGAGCAGCATGCCCCCGAGCT
       ::::  :: ::::: :: ::  : ::: :  : :  ::  :  : ::::: :  ::::   : :::::::  :: : : :
HYALO  AACGCCGCTCTGGACTG-TAATTTCGC-TTCACAGTTGAACAACAGCTCA-ATACTGTCTCAACAGCATGAGCCAGCGTT

          4180      4190      4200      4210      4220       4230              4240
PINFES GTTACCTCGTCTCGAGCGAGCACAACAGGCTAGTGCCTTTGG-ATCAGCTTCGCACCACC--------CTGCAGAGGCCA
        ::   : ::::: :      :  :    : :::::  :::  : ::   :: : : :::        :: ::   : :
HYALO  CTTCAGT-GTCTCAAAATA--AGGAAGAACCAGTGCTCTTGATAACATGCTCTCCCGACCGTCGAACTCTTCACTCGTCG

    4250          4260      4270      4280      4290      4300      4310      4320
PINFES AGAT----GGCTACAAGCTCGTACACTCCGTCAGCAGATACCAAGATGCACTACTCTGCAGAGAAAAAGGAGTCGACGCT
       :  :    : : : : : :::::::: :  ::  :   : :  :: ::: ::   :: : :: :   :   ::::  :::
HYALO  ATTTCTCCGCCCAGATGGTCGTACACCCATTCGTCCATTTCTCAG-TGCGCTTTACTTCGGAAAGGTAAATGTCGGAGCT

        4330      4340      4350      4360      4370      4380      4390      4400
PINFES CAGTGCTGAAGGTGATGACGCAGGACGCGGTACTAATGCTCTGGATGCGTGGATGCAGCGTGGGGAATGGGACAAAGTGT
       : :::::   ::    ::::  ::  :::  : ::   : :: ::::: ::::::::: :::    ::::: :::  : :
HYALO  CCGTGCTTGCGGGTGCGACGATGGGTGCGACAGTACCACCCTTGATGCATGGATGCAGTGTG----ATGGGTCAACTTCT
```

```
        4410       4420      4430      4440       4450         4460       4470
PINFES TGTCT-TCAGCAGCCAAGCATAGTGCTGGGTCGCTGGCGA-AGTACC---TCGTTCTTCGGTGC-TCGCGACTGTGCGAG
       : ::  : : :  ::: :::    :::: :  ::::: :: :: :     :::        : : : ::::      :
HYALO  TATCACTTAACTACCA-GCA----GCTGCGAAGCTGGAGACAGGAAAGAATCGAGGCAGAATCCATGGCGATATACTGCT

    4480      4490      4500      4510      4520      4530      4540      4550
PINFES CATGGCGAGATGTCTACGGCGATCAAGACCATCTCTGACTATGGCATTCCTCTTGACTCGGAGGCTCTGGATATGACAGA
       : :  ::   :: :::: ::::: ::          : : :::: :  : : :: ::: : : :       : ::: :::
HYALO  CCTTACGCC-TGGCTACCGCGATGAA----------GTCAATGGTAGGC-TGTTTACTTGAATGTAAAAAGTGTGAAAGA

    4560      4570      4580      4590      4600       4610       4620      4630
PINFES GAGACTCGTCCAGAAGACGCTTGCATGTGGCTACACCATCGAAGAG-GATTCTGAACA-CCAAGCGGCGTTGCCAGA
       :::::: : : : :::  : ::: :: ::    ::   : : :::: :: :   :::  ::::   :  ::  ::::
HYALO  GAGACTTGGCGA-AAGGTGGTTGAATTTGCGGTCAGGCTGGTAGAGAGAATGCAAACGTCCAATTAGTTTTATCAGA
```

gap....

Waterman-Eggert score: 142; 34.9 bits; E(1) < 0.0023

53.7% identity (53.7% similar) in 257 nt overlap

```
          190       200       210       220       230       240           250       260
PINFES GTGACAAGAATTACATCGTGCGTGCACTTGAGTTCTCGCCGGATTCGTCCAAACTCGCGGTGGCA----CAGAGCGATAA
       : :::: : : : : : :  :   : : :: ::::::    :::   :   :  :  :::: :::    ::::: ::  :
HYALO  GGGACACGGAATGCGTTGCACTCTCGCATGTGTTCTC----GATAATTTTGAGGTATCGGTTGCAATTGCAGAGTGACGA

              270       280       290       300       310        320       330
PINFES CATCGTCTTTGTCTACAAAATTGGCCTAGAATTTGGGGATAAGAAGAGCATTT-GCAACAAGTTTCTACAGCCATGCTCC
         :    ::    :::::  :     : :::    : ::   :::::: :::: :: :: :::::  : :::  : :
HYALO  ATTGCGATTCAGTTACAAGTTGTAGATGGAAGAGCGAGACTGGAAGAGTATTTTGCTACGAGTTTGCAGAGCACTACCTG

     340       350       360       370       380       390       400        410
PINFES GTGACGACACTCACGTGGCCCAGCACACACCCTAACGAGATCGTCTTCGGCCTGGCAGACGG-CAAGGTGAAAGTCGGTC
        : : ::  :: :  :     ::  : :      ::::: ::   ::  : :::: :::::  :  : ::::  :::::
HYALO  TTCATGAACCTTAT-TAATGAAGTGCGCGAGTAGACGAGGTCAGTTTGAGACTGGTAGACGAACTTGTTGAACCTCGGTT
```

```
      420           430
PINFES AGTT----GCGTAGCAA
       ::::    ::::: :::
HYALO  AGTTTGCCGCGTACCAA

gap....
Waterman-Eggert score: 138;  34.1 bits; E(1) <  0.0043
56.6% identity (56.6% similar) in 279 nt overlap

          2680      2690      2700      2710           2720       2730      2740
PINFES GAGGCTGCACTTAACTCACGACAATGGGCTAAGGCCAGTCAACTT-----GTAGAGACAC-TGGAGGACGATGTGTCTCT
       :: :: :  : : ::::: : ::::::  : : : ::   ::: :     :: : ::: : :  : :::  :   :: ::   : ::
HYALO  GACGCCGATCCTCACTCAAG-CAATGGTGTCATGTCAACTAACATCGCCGGTCGTGACGCGTTAAGGCCACTG---CACT

      2750      2760      2770          2780      2790      2800      2810      2820
PINFES ACCATACTACCGTCGCCTTGCTCGTCAC----TACCAGGAAGCAGGCAATCTAGAGCAGGCTGAGCGATGCTTCATCAAG
        :: ::: :: :: :    :::    ::     :   : :: ::: : : ::  ::::::::: :::  : :  :  : :
HYALO  TCC-TACGACTGT-GAAAAGCTGAGTACATTTTTATAAGATGCAAGTAGTCCTGAGCAGGCTGGGCGTCGATCGAAGA-G
          2830      2840      2850      2860      2870       2880        2890
PINFES GCGGACGCTGCTCGTGATGCCGTCGAGATGTACACTCGAGCCAACAAATGGGA-TGCTGCC--TACCAGGTCGCACTCA-
       :     ::  :::  :: :: :: : ::::: ::: :: ::: : :::   :: ::: : :  :::     :: :  :: 
HYALO  GTATTTGCCACTCACGACGCTGTTG-GATGTGCACACGTGCCGATAAACAAGAATGCAGTCAATACATATACGAAAGCAC

     2900      2910       2920        2930
PINFES -ACCACTTGGATAAAT-ACGAGACCGAGA--GACTGTAC
        :  ::::   ::: : :::::  : : :  :: :::::
HYALO  GATGACTTCACTAAGTCACGAGCACCAAATTGATTGTAC

gap....
Waterman-Eggert score: 119;  29.9 bits; E(1) <  0.076
60.2% identity (60.2% similar) in 118 nt overlap

       2140       2150       2160      2170      2180      2190      2200      2210
PINFES ATGAAGCT-ATTGAGATGT-ACCAGCACTTGCACAAATGGGAAGATGCTATCCGCGTTGCTGAGACGAAGAACCATTCCG
       ::::  :: :::: : : : :: :  ::   : ::: : : :::::  :   ::: :  :: : ::::  :  :::  ::
HYALO  ATGACACTGATTGTGTTATCACTACTACGGACTCAATTTGCAAGATATTGCACGCATACCTCATACGACTAGTCATCTCG
```

```
       2220      2230      2240      2250
PINFES GTTGCG---AACAGAT--GAAGAGAAGCTACTATGAAT
        ::: :   ::: :::  :::  :::::: : : : ::
HYALO  ATTGTGCAGAACTGATTTGAAACGAAGCTGCAAAGGAT
```

```
PITG_5499
Waterman-Eggert score: 528;  102.4 bits; E(3501) <  1.4e-21
56.5% identity (56.5% similar) in 864 nt overlap

          640       650       660       670        680       690       700       710
PINFES AATCGTTAAAGGACCAACTCGCGATGAAATCTGCGGAA-CAAGAGCGCGAGACACGACGCAAGGAGCGACTGGATAAAGA
       :: : ::  : :::  ::  :: :::     :::: :: :: :  :: ::     : :: :: ::  :  : : : ::::
HYALO  AAGCTTTGGAAGACTTACCAGCTATGG----TGCGCAAACAGGTTCGTGAATTGAGGCGTAATGATTG--TTGGTGAAGA

           720       730       740       750       760       770       780       790
PINFES GATCAAAGACGTCAAGAGTAAGATGGAGAAAAAGACTACAGAGCAGGCTCAAGGCGCGACAGAGCTTACGCGCTTACAGA
       :   ::::  :::::::::: : ::::       ::::: : : :::   :: ::  : : ::: :::   :::   :  
HYALO  G--TAAAGGAGTCAAGAGTATG-TGGAAGCGGCAACTAC-GGGGAGGTCAAACGCAAG-CGGAGTTTATCTGCTGCAAAG

           800       810       820       830       840       850       860       870
PINFES CACAAGTTGGAGCGCTGGAGAAGCAGCTCGCGGAGGCGCAAGGTACGATGGACAAGTATGTGCGAGACTATGAGACGCTG
        : ::  :::  :  : ::::    ::     ::  : :   :: ::::::   :::: ::    ::: :  :  :::::
HYALO  GAAAA--TGGGTCATTTGAGA----GCGATGCGATACTCGTAGTGCGATGGGATAGTACGTCTAGGACAACAACGCGCTG

           880       890       900          910       920        930        940
PINFES TTCGGACGTACCCAGAAGCTCACGGAT---CTCCTGAACGAGCAGAATGATAAG-ACGATGCAACTTGA-AGTAGAGCGA
       ::::::::   :     :  :: : :    :::   ::: ::::::: ::   : ::  ::::::::   ::  :::  :
HYALO  TTCGGACGCGTCAGTTCGTGCATGCACTGACTCGCTAAC-AGCAGAACGAAGGGGACTGTGCAACTTCTGAGGGGAGGTA

      950       960       970       980       990      1000      1010      1020
PINFES CGCAACTTGGAGAACGAAATCAAGGCGCGACACGAGGAAATTCACCGCGTGAAGCTTGAGAAGGGCACGGTGGAGCGCAA
       : : :::: : :  : :   ::::  : :     :   : ::   :::: ::::::  ::   ::::  :  :: : : :
HYALO  CACTACTTCGGGGGC-AGCAGAAGGTTC-AGCGTAATTACTTTTTCGCGAGAAGCTGTAGGTTGGCATCGAAGAACACGA
```

```
     1030          1040      1050      1060      1070      1080      1090      1100
PINFES ATT----GGACAAAGAGAAGCGACTTCTGCAGCAACTAGAAGAAAAACTTGAGGGCGAACACACGGCCAAGATCGTGCTG
       ::     :::::: : ::::::::::     ::::: :::::  : :              ::: :: ::: ::: :: :
HYALO  ATGAATAGGACAACG-GAAGCGACTTT----GCAACAAGAAGCCAGA--------------CACTGCAAAGGTCGCGCAG

1110      1120      1130      1140      1150      1160      1170      1180
PINFES CAAACGCAGATCAAGAGCATGCAACACGATCTGGATCAAGACAAGATCAATGAAGACCACACACGAACGGAGTTGGAGGC
       ::: :    :  ::: : : : ::  : :::: :   :: ::: : :   ::       : :::: : :: : ::
HYALO  CAAGCTTTTACGAAGGGTACG-AATGC-ATCTTGG--AAAACA-GTTTGGTG-------CGCACGTATGGCGGTG-----

         1190      1200      1210      1220      1230      1240      1250      1260
PINFES GTTAGAACGAGAGAAAGCTATGCAAATGAAGCAGACGCTGAAGGTAGAGGGCAAAGTTCGTCGTGCTCAGGATGAGATCA
              :   :::: :::::: :::  :: ::::: ::::::: :::  :::: : : :::: :   :::  : :::::
HYALO  -------CACAAGAAGGCTATGGAAAACAACCAGACACTGAAGGCAGAAAGCAATGATTGTCGAGTGTAGGGAG-GATCA

         1270      1280      1290      1300      1310          1320      1330      1340
PINFES AGACCAACGAGCGTGTCGCAAAGAACTTGGAGCAAGAGCTGGCAGG--ATT--CAAGCTCGAAGCTGCCAAGCAACGTAA
       :::: :: ::::::: ::    :  :::  : : ::::   ::: :  :::  : :::  : :::    ::::: : :
HYALO  AGACGAAAGAGCGTG-CGGTGTGGGCTTTAACCTAGAGTGAGCAAGTTATTGCCGAGCAGGTAGCCAA-AAGCAGCCTCG

             1350      1360      1370      1380      1390      1400      1410      1420
PINFES ACTTATCTATCAGCTCGAGAAGGAACGCGAGAAGTATGGCATGGAGGCAGCCGAGCAACGTAAACTCTACTTGCAAGCTG
         ::::: :  :  :     :  :: :::  ::: :::: :: ::::  :: ::::: : : ::   :   : ::  :::
HYALO  T-TTATCAAGAACTTTACTCA--AAAGCGGAAAGGATGGTATCGAGGTTGCAGAGCAGCTTGAAACGTG--TACAGTCTG

             1430      1440      1450      1460      1470      1480
PINFES AAGAAGAAGGGAAGTTGAAGGAAATGCGAAACCACGACTTGCAAAAGAAGGTGCATGAAGGAGA
       :    ::::  :: : ::::::  :::::   :: : :::::::: ::  :: ::: :  : ::
HYALO  A----GAAGCCAAATCGAAGGAGGTGCGAGGACATGGCTTGCAAACGAGAGTTCATAAGTGTGA
```

PITG_8038

Waterman-Eggert score: 166; 26.3 bits; E(1) < 0.025

59.0% identity (59.0% similar) in 183 nt overlap

```
               10        20        30        40            50        60        70
PINFES TCAATGCCCAGCACAAGACCATTTCACTTGGCAGTTGTCCATT---CGA-GTCTGCGAGCAGATCGAGAGCCATTTGCAC
       ::: ::   :::    : :::::   : : ::::  ::  :::   ::: :::: ::: : : :: :::::: ::::::
HYALO  TCACTGTTTAGCGAGGGGCCATT---CGTCGCAGCAGTG-ATTACACGACGTCTACGAAC-GGTCAAGAGCCGTTTGCAA
```

```
          80        90       100            110       120       130       140       150
PINFES TGACATGCGTACGCAGTAAAATCTGG--CGCG---ATTTCCATTTTCACTCGAAGTTTCGCCTCGCTAGGCACATTCTCG
       :: :: ::: :::: : ::   :: :  ::::   : : ::  ::: : ::  ::  ::   :: ::   : :  :   :
HYALO  TGGCACGCGCACGCTGCAAGTGCTTGGTCGCGGCAACTCCCCATTTGAGTC--AGCATCTA-TCACTCAACGCTGTAAAG

             160       170
PINFES CGGAAGAACATGTAGTCGACACT
       :    :: ::::::: :  :: :
HYALO  CATTTGAGCATGTAGGCATCAGT
```

PITG_12891
Waterman-Eggert score: 291; 39.4 bits; E(1) < 4.6e-06
54.0% identity (54.0% similar) in 665 nt overlap

```
               20        30          40          50          60         70        80
PINFES TCCACGACTGCTGGACGAGC-CGGGGTGC-CGCA--CTTGTACGTCTACA--GCAGCTTCTC-GATGACGCCGAGGCCCA
       : :: : ::: :: :::::  : :: ::: :: :  : : : :::  :::  : ::: :  : :::  :: :: : :  :
HYALO  TACATGCCTGGTG-ACGAGTGCTGGATGCTCGAATGCGTCTTCGTGAACAAAGTAGCGTGACAGATT-CGTCGGGACGGA

            90       100       110       120                        130       140
PINFES AGCCGAGACCGAACGCAAGGCCGCGACACACATCCAGCGG-----------CTTTTT------CGAGGACAACGTGTCCG
       :  : :: :     :: :: ::   :::: :   ::::            ::::::      : :: ::  :   :  
HYALO  AAGCCAGCCATTCAGC--GGACGTTCCACA-ACAGAGCGTATGTGTGCCACCTTTTTTAAACCCAAGCACCGCCAACCGA

         150       160                       170       180         190       200
PINFES T-GCTGCTGTCACAGC-----TCAGACA-----------GATGCTGAACTCCTGATCTCT--CGTGTGTTCCGAGGACAC
       : : : ::::::: :      ::: : :           ::::: :::     :: ::::  :::: :::   :   : :
HYALO  TAGTTACTGTCACTGAAATCTTCACAAATCTTCGTTAGCGATGCAGAAGGAGCGAACTCTTGCGTGAGTTTTTACAGCGC

       210       220         230        240         250       260       270       280
PINFES CTCGCACGACGACGCTGTAA--TCAACTACGCA-CTG--ACTTGGAGCGAGCGCATCGCCGCTCAGTATTGGATTACTAC
       :: :::         :: ::  :::  : ::   :::  :  ::::  ::   :: :   :  :::: ::: ::: ::  
HYALO  CT-GCAA--------TGCAAGTTCACTTGCGTTGCTGGTATGTGGATAGATGCCAACATTGG-CAGTCTTGAATTTCTG-
```

```
            290       300       310       320       330       340       350       360
PINFES GCTGTAGTCATCCAGAAACTTGCACGCGGGGTTCAAAGTCGGACACTTCGGCTCGACTTCCGTAAACGGAAAGCTTATGT
       :: :: : : :: :  :: :   :::::     :  ::: ::::        :: ::  :   :: ::   : ::::
HYALO  GCAGTTGACGTCGAATAAAT---ACGCG-----CTGAGTAGGACGAA----GTCAACAACA-AAGCGATGACCTTA--C

            370       380       390        400         410       420        430
PINFES TTTGGAACTTGCAGCCAAAGGAGACCTCATG-CGTCAAATGCTG--GAAGAAAACCTACTTAAGCAA-CAAGAACAGGAG
       :    ::::: ::::::   :::::    :: : ::  ::::::  : ::::: :  :: :::::: :: :: ::: :
HYALO  TGACAAACTTACAGCCATGTGAGACGATGTGGCATCGGATGCTGTTGGAGAAATCGCAC--AAGCAAACAGGAGCAGAAC

      440       450       460       470        480        490         500       510
PINFES AAGCTAGACACTAAGAAAGCCGCAAGAGCGGAGCTGGTCAA-AGTC-ACTCAAGA-TCTCCAT-CACTTGGTCAGCACGC
       :: ::::: : : :::  :: :  :::    : ::::::   :: : : :::::  ::: ::: ::::    :: ::::
HYALO  AA-CTAGAAAGTGAGAGGGCAGTCAGACTAAAACTGGTCCCGAGACTAGTCAAGGGTCTGCATACACTA---CA-CACGT

          520                 530             540       550       560       570
PINFES GTGCTGTG----------GCTGGCATC------TTCAACTCGCCCATGCAGGCAGCAAGCGTCACGAGTTTCGGTGTCTC
        : : ::           :: ::::::      : :: :: :::::::::   : : :  :::::   : : :   :  :
HYALO  TTACCGTCACCGAGATCTGCAGGCATCGCAATATACAGCTTGCCCATGCAAATATCCACTGTCACTCTTGTTGACCTGCC

580       590       600
PINFES -TGTAGAGAGCCACATTCGAGAAAA
        :: ::: : : : ::::: :::::
HYALO  GTGAAGAAAAC-ATATTCGCGAAAA
```

PITG_13069
Waterman-Eggert score: 138; 24.2 bits; E(1) < 0.084
55.9% identity (55.9% similar) in 211 nt overlap

```
              230        240       250        260       270       280        290
PINFES AGGACACAGAT-TGGATCTATTTCAGTGTACT-CGCCTCTAGGGTTTAGGTAGAACAGGTGTTTGACGC-CGGTCTTGAG
       :::: : : :: ::::: : ::  : :: : : :: ::      :: ::   ::  ::: : : ::::  ::: :  :::
HYALO  AGGATAGATATGTGGATGTCTTGGA-TGAATTGCGACTTGTTTATTGAGAGGGATTAGG-GCTGGACGAACGGACACGAG
```

```
       300       310         320       330       340       350        360       370
PINFES GAAACCCATGACAGCACCATC--GTCATCATTGATGGCCAGCAGCAGTGTCTGTTGCTGTTCT-GTTGCTGTGGACGTCG
            :  : : :::: : :   ::  ::  :  : :     : : :: : ::    ::::   :::::::::::: :::
HYALO  TCGTTCGTTCAAAGCATCGTGAGGTACTCTATTCTAGTGTTAAACTGTTTTTGGAATTGTTGACGTTGCTGTGGACTTCG

          380       390       400            410       420
PINFES CAAAGAGATCGGTGGAGGTGATTTCATGGGAG-----AGAGTTTGGGCTGT
       ::::::::   ::  :    : :: : :  ::     : :: :::: ::::
HYALO  CAAAGAGA---GTTCA----AATTTAGGTTAGTATTCAAAGCTTGGCCTGT
```

PITG_10796

Waterman-Eggert score: 241; 28.8 bits; E(1) < 0.011

50.6% identity (50.6% similar) in 1369 nt overlap

```
          220       230       240       250       260           270       280        290
PINFES TCTCGTTGGCAACGCGTTCTTTTTCTCGACGCAGGGCCAACTCGTCCAG----CAGCACCTTGTTGTGTCTCTTC-CAGA
       :: :: ::: :::: :: : : : :: : : ::::  :::   ::  :     ::::: ::  ::: :    : : : ::
HYALO  TCACGCTGGAAACGTGTGCATATGCTGG-CTCAGG--CAATGTGTTGAAGGTTCAGCAGCTC-TTGCGAACTTGCGCCGA

              300       310               320       330         340               350
PINFES AGAGATCGAGGTATTCCCACT----CGC----TCACAGCGACGCCGTTCT--GCAGTCCT-TG-------TGGCTGGAA-
       : : :: ::::   :::   :    :::    ::: :: :  ::::: ::  ::: : :: ::       :::  : ::
HYALO  ACACATTGAGGA--TCCTGGTGAGGCGCAACATCAGAGTGTTGCCGTCCTTGGCATTGCTCTGATTACGATGGGCGAAAA

                  360       370       380       390       400              410
PINFES -----GTCGAGCATTTGCAGCGCTTCTTCGTCCTCACGACCGGTTTCTGTACGCAT------CGCTA-GTCGTACAGTTT
            :  : : : : :::  ::    ::::  : :: ::  : : : :::::         :: :: : ::  ::::
HYALO  AATTGGCAGTGAAATGGCAATGCG--GTCGTT-TGACCACTTGCTGCAGTACGGTGAAGTGGCGGTACGCCGAGCAGTGC

     420       430        440       450       460         470       480       490
PINFES CTGTAAGTTCGTTCGCT-TCCTGTTCGTCGGCCAACGTCGACTG-GTAAA-ACGGAAGCACGCGCTCTCGCTCC------
       :: :     : :: ::: :  ::  ::: : :: :  ::  ::: :::   :: ::   :: :::: :::: ::
HYALO  CTCTGG---CATTTGCTCTATTGAGCGT-GTCCGA--TC--CTGAGTACGCACTGATCGACACGCTGTCGCGCCTGACCC
```

```
                       500        510       520       530       540          550
PINFES --------GTCTCCAGT-TTGCGTACAA-GTTCTCCAAGCTGCAGGATCCGCTCGCTCAGCTG---CAAATGAGCGTCCA
               : :::  :: ::::  : :: : : :::  ::: :  : :: : :   ::::: :   : ::: :    : :
HYALO  ACGATGTTGACTCGGGTGTTGCTCAGAATGCTATCCTCGCT-CTCGGTCTGGT--ATCAGCCGGCACGAATAACTCGCGA

       560       570       580         590       600              610       620
PINFES GTTTCTTGGAGGCTTCGCGAGCT--CCGTGCGATAGCCCTGCGAGTCGACG-------TCGCTGGTCGTCCCGCATGCGG
       ::  :: :   ::::::: ::::  : : :   ::     : ::: : :         ::: ::  :::  ::::   ::
HYALO  GTGGCTGGTTTGCTTCGCCAGCTTTCGGAGTTTTATAATCGTGAGGCAAACCATCTGTTCGTTGTGCGTATCGCACAAGG

      630            640       650       660       670       680       690       700
PINFES TT-----CATTTTGGCTTTGAGCGTCTGGAAATGATGCGAGATCGCCGCTTTCTCCTCTTCTAGCGCCTTGTTACGCTCC
        :     ::  : :::    :: ::::   ::::: ::     : ::  ::   :: ::   : ::  :  : : :   :
HYALO  CTTGCTGCACATGGGCA---AGGGTCT---AATGACGC---TGCACCCGTT---CCACTCGGACCGATTGATCATGTCTC

           710           720         730          740          750       760        770
PINFES GAGCACTCGCGTACA----TTT--TGAGCGATCTT---GGTCCTCCAGA---GCGCCAGACTCTGTTGCAGACG-CTCAA
       : :   : :::  ::    :::  :::    ::::   : : : : :::   :   :: ::: ::::: : ::  : ::
HYALO  GTG---TGGCGGTCAGCGGTTTGCTGACAATTCTTCACGCTTCGCTAGATATGGAGCATACTATGTTGGACACTTCCCAC

               780       790       800          810       820       830       840
PINFES TCT-TGCGGATCTTCAGCTCGATATCACGGGACAGATCC---TGGTCCTTCGCCGTCAGGTACTTGAAGTCCTGTGTGCG
       : : : : :  :: ::   : : : ::  :  :::  ::   :: :: ::   : : :      ::: :  :::  : ::
HYALO  TATATTCTGTACTGCATTGCTACAGCAATG--CAGCCCCGTATGCTCATTACGCTTGA------TGAGGAGCTGAATCCG

        850          860       870       880       890       900       910       920
PINFES CTGA---TCTGTGTTCTGCAGGTAGTTGAGATGCGCCGTCTCGAAGTGTTGCTCTAGCTCCTCAATGCTACTGTCCAAGG
       ::     ::: :::::           :::  :  : : :   : :: ::       : : ::: ::   :  ::
HYALO  CTTCCTGTCTCTGTTC-----------GAGTCGGCCAGGCGGTACGTATT-------CACTTCATTGTAGCGATCTTGAT

              930       940       950       960           970       980       990
PINFES TG---ATCCGCAACACGTTGATGTCTTCCAGATTCTTGTTCCGG----ATCTCCTCTCGAAGCTGTTCGTGCTCCTGCTT
       ::   ::: ::: : :::     :::  ::  :::::: : :::    ::: :   : :::  :::  :    :: :  :
HYALO  TGGCCATCAGCA-CTCGT-----TCTAACAT-TTCTTGCTTCGGTGTAATCGCAGGTTGAAATTGTCGG----CCAGGCT
```

```
       1000      1010           1020       1030      1040      1050      1060      1070
PINFES GGCGTCGGCCTCACGCTCCTTC-----TCCTGCTG-ATCTACCGCCTGCATGATGCGCTGCAGCTCGTTACGCTCCATGG
       :::  :: ::  :  ::  : :     ::: :  : ::  : ::::::        ::::: :   :: : :  ::
HYALO  GGC--CGCCCGAAGTCTATTACGGGTTTCCAGACGCATACAACGCCTGT-------GCTGCTGAATGTAAAGGACC----

             1080      1090      1100      1110      1120      1130      1140
PINFES TGTGCTGAGCGATGAGTTTGTTCTTCTCCGTCTGGAACTCTAGCTCCAGCTCTCGTAGATCGGCTTCAAAC-----TCTT
               :::  ::: :::        :: : :::    :: :::: :::  :: :  :  :::  ::       :: :
HYALO  --------GCGCAGAGCTTG--------CGAC-GGACGAGTACCTCCCGCTTACGAATGT--GCTGGAAGGCGTTGTCAT

        1150      1160        1170      1180        1190      1200      1210      1220
PINFES TCTCTAGACCTAATA--GACGCTCATCTTGCAGGTCGATCAGCTCG--TCGACGTTGAGGAGGTGGGCCCGCAGAGCCAT
       ::::  ::   :::   ::   ::: :  :   :::  : :::  :  :  : :   :::::::::  : :    ::::
HYALO  TCTCC-GAAAGAATCCGGATTATCACCCGG--AGTCT-TAAGCAAGCATTAAAGA--AGGAGGTGGAACGGTTTGGCCAA

                   1230             1240      1250         1260      1270      1280
PINFES -CTGGAA------CTGC---TCCTCGGC----TTCCTCCAAGTCGCGATC---GAGCATCTGGATGAGCGCGTCTTTGCG
        : ::::      :::    :::  : :    ::::  ::::: :::::    ::::: :    :::   :  :::: ::
HYALO  ACAGGAAGGGGCGCTGTCGTTCCGTGACCCCATTCCGTCAAGTAGCGATAACGGAGCAACACACTGAATAC--CTTTACG

         1290      1300       1310      1320      1330      1340        1350      1360
PINFES ATCCACGTCGCGCTCGTGATTCTG-AGACCGCACCTCGACCTCTTTACGCAGCGCCTCCACCT-TGA-CAAGCCTCATGA
        :::  :: : :  : : :::::  ::  :  :: :::       :  :  : :: : :  :: ::: ::::   :  ::
HYALO  CTCCT-GT-GTGTCCATCATTCTCTAGTTCTAACGTCG-------TGTGTTGTGCATGCTGCTGTGAACAAGTTGCTGGA

1370      1380            1390      1400      1410       1420       1430
PINFES TTTTCCGCCAATGGTTCTGGA----TCTT-GAG-CTTGTTCATACGAGAGATCTTC-TCCTCTTTAG-CCATGAACTCCT
            :: ::::::   : :     :::: ::  :: :: :::::::     :  : :::::::: : :: :  : :  :
HYALO  -----CGACAATGGACGTAGTACGCTCTTCGACACTCGTACATACGATTATGCGACGTCCTCTTTCGGCCGTTCAGTTTT

          1440
PINFES GCAGTAGGG
       ::: :  ::
HYALO  GCACTCAGG
```

PITG_02417
Waterman-Eggert score: 164; 24.8 bits; E(1) < 0.12
51.4% identity (51.4% similar) in 863 nt overlap

```
            40        50        60        70        80        90       100       110
PINFES TGCATTTGCGGCACGTTTGGCATTGAAATTGGCATTGTTGCCAATTCCTGGTTGCGGAACATTTGCATGGTTAAGACGCC
       ::::   ::::  :::::  : ::   : ::: : ::  :       :::::: ::::   ::: : :: :   ::::
HYALO  TGCA---GCGGATCGTTT--CGTTTGGAGTGGGAGTGGCGGTCGGAGCTGGTTACGGAC--TTTACGTGCT---GACGGA

           120       130            140          150       160       170       180
PINFES GCATGCCCTTGAGTGTACGCACTTT-----GAGTTC---GTCTCGTGTGAGAGGATGCATCGCGTCGTCATTATCTC-CG
         ::  :   ::::  ::::::        ::: ::   :  : :: : :: ::: :    :  : ::::  :: :  ::
HYALO  CGATATCGAGGAGTCCACGCACCAGATCCAGAGCTCCATGGGTAGTCTCAG-GGACGAGATGATTGGTCAAAATGTGGCG

                  190             200       210       220       230        240
PINFES CTATC--------TTCA--CCCTCG----GAGGAATAATCGTCGAGGTTTGGCGGGTTGATCTG-TAGCGGTTCCTGAC-
       ::  :        :: :  : ::::    :: ::  ::  :  ::     : : ::  :: ::: :::: : :   :::
HYALO  CTTACGAAGCGCATTGAAGCGCTCGAGGAGAAGACCAAGTGAGGAAAAGAGACAGG--GAGCTGCTAGCTGATGGAGACG

                   250       260        270       280       290       300       310
PINFES -------------CCATTGGTGCAA-TCGGACCGACCCCAAGGATCGAATGCAAAGTCGCCACAGCCCCGTTCGTGCCGC
                    :::::::::    : ::::       :::::    :::  ::   :  :  : : : ::: ::  :
HYALO  CTAGAACACTAAACCATTGGTGGTGGTTGGACGAGAGAGAAGGAGGAGATGGGAA--AGATAATGGCTC-TTCTTGATGT

          320       330       340       350       360       370       380       390
PINFES CTGCAGCTCCAACTGCCGCTGCAAACTGTGATTCGCGATCATAGGCATCACTGGTAGGTTTGCTCTTCTGGGCATCT---
         :::: ::  : :       ::  :: :  ::::        : :  ::::  : : ::: ::::  ::   :::
HYALO  TGGCAG-TC--AGT-------CATCCTCTCGTTCG--------GTCTACACT--TGGTTTTTCTCTCTTGTCTATCACTT

               400       410        420        430       440       450       460
PINFES --GCCTGTTGCCTAGTGACAACTGCTG-CGTACTGGT-GCAAAATCTCATTGGTTTTCTGCTCAATGACACCCAAGTAAG
         :: :: : : ::::      : ::  :::::     ::  :   : : :::::: ::: ::        ::  :: ::
HYALO  GAGCTTGATTCATAGTT-----TACTCACGTACCATCAGCGTACGATAACTGGTTTACTGTTC--------CCGTGTCAG
```

```
       470       480       490                  500        510       520       530
PINFES CCATCATGTTCGCTTCCGTGACTACC-------GAATCG----CCCAACATC-TCCACCATGGCTTGTGCGTTGCATCCA
       : : : :::  :::  :::::: : :       ::  ::    : : : ::: : :::  :: :  :   : :::::: :
HYALO  C-AGC-TGT--GCT--CGTGACGAGCTGTTGCAGATCCGTAGACACGAGATCGTACACTTTGTCA-GCTAGCTGCATCGA

          540          550       560          570            580           590
PINFES -GTTTTAG---TGAACAAGTTCTGGATTGCACGTTTG---AC-AGTGTTCA----CTGTCTTCT----GAGCTTCTGCA-
        ::   ::   ::  :::   :: :::  : : :  :   :: ::  ::::    ::: ::: :    ::::: :: :: 
HYALO  CGTGAGAGCGCTGTGCAACGACTCGATGTC-CTTCCGGAAACTAGCATTCAGCTCCTG-CTTTTCCTCGAGCTGCTTCAA

      600       610       620       630       640       650                   660
PINFES CAACGAGTCTCACACTTTTCAGCTGCTTGTTCCGTGAGTGCAAGTCGAGTTTC--CAAG-------TC---ACGTAGCAA
       :::: : :   :: :      :: :::::::   :::  :::  ::: ::: :  ::::       ::   ::::: :  
HYALO  CAAC-ATTGCGACGC------GCAGCTTGTT---TGACCGCAGCTCGCGTTGCAGCAAGCAGAAGATCGCGACGTACCGG

        670        680        690       700       710        720       730          740
PINFES TTGCTT-GTGTTGGCCAACA-CCATTTTCATCTGCAACTCCATTGGCTCCGTC-GCCGTAACGACGTT--CTTCATCA-C
       :  : : ::  :: :  ::: :::: :::    ::  ::  : ::  :: ::: ::: : : : : :   ::  :::: :
HYALO  TCCCGTAGTACTGTC--ACATCCATATTC----GC--CTTGAGTGTGTC-GTCTGCCATGAAGTCCTCGACTAAATCATC

               750       760       770       780       790       800
PINFES G-TAGCGCTGCGATCCATTCCTCGAGTTTCTCAATCTCGTTGCTTTGTTCGTTGACATAGTTA
       : ::::::::         : :: ::  ::::: :  :   :::::  :::   :::: : ::
HYALO  GATAGCGCTGG--------CGTCAAGCATCTCAGTAGCAGCGCTTTCATCG---ACATTGATA
```

PITG_10664

Waterman-Eggert score: 290; 68.1 bits; E(1) < 3.6e-14

56.8% identity (56.8% similar) in 454 nt overlap

```
     2660      2670      2680      2690      2700      2710      2720      2730
PINFES GCTGCGTACTATCACTGGAGACTCTGCGAGCAGCAGATGAAGACCGTATTCTCCCCACAGCAGCAAGTGGAAGACGGCAA
       :::: ::  :::::    :::::::   ::  :   : : :: ::   : :   : :  :    :::   ::: ::: :: 
HYALO  GCTGAGTT-TATCAA---AGACTCTAATAGGAATTGCT-AAAACTTAAATGAGCTCTTAATTCCAAACTGAACACGTCAC
```

```
    2740      2750          2760      2770       2780      2790       2800
PINFES GGAGCTGAGTGAGGAGCAACG----CTATCGACTCGCAGAAGCC-ACGGAGAACGAGCTCT--TGTCGGAGCTCTAC---
        :: ::   ::: ::::::::    : :: ::::    :: ::: :::    :: : : :   : ::: : :::: :
HYALO  AGATCTAGATGATGAGCAACGAGCACGAT-GACTTAATGAGGCCCACGTCAGACAAACCCAACTTTCG-AACTCTTCCTT

         2810      2820      2830      2840      2850          2860      2870      2880
PINFES ---TACGCGTACGCCATGGTCTACGCCTTCACGGACGAGCCCTTCACCACTTTA----CAGCCCGAGTCGCTCTTCCACG
         : : : : ::::::: ::: : : ::  : :: : ::: :: :: :: :::    ::: :: ::: ::::    :
HYALO  ATTTCCACATGCGCCATGATCTCCACGTTTTCCGATG-GCCATTTACGACATTAATAGCAGTCCAAGT-GCTCCGAAA--

            2890      2900      2910       2920      2930      2940      2950      2960
PINFES CTGCTCGCTTCCTCCTCAACAAACTGTCAGCTCGAGG-CAGCAACGGCAGAGCGGGAGGAGGCAACGGCAACGCAGCAGC
        :: :  ::  :: :::::::  ::::::  :   : :: : : :::: :   : :  :   :  :   :: :  ::::
HYALO  --GCACCTTTTTCGTCAACAATATGTCAGTGCATCGACATCGA-GGCATAATAGAATAATAAAGAGAAGACACGACAGC

             2970      2980      2990      3000      3010          3020      3030
PINFES GACGGCGCCTCCTCCTCCTGGGATCTCGCTCAGCAACATCTACTTCACTC----TCGGACACCACGCACTGCAGCTCGAG
       ::::::   ::: ::::  :  :   :  :::   ::: :   :::: ::    : : :::: : ::  :::: ::: ::
HYALO  GACGGCTTTTCCGCCTC--GATACAGCTTTCAAATACAACCCTTTCAGTCATACTTGAACACTATGCTTTGCACCTCAAG

       3040      3050      3060      3070      3080      3090
PINFES AGCTACAAACTGGCGCGACAGGCGTACGAGAAACTGCTGCAGATGCGGCTGAGA
        :::::   ::::  ::::  :: : :   :: :: :: ::: ::: :  : ::
HYALO  TGCTACGTGCTGGTACGACGCGCTTGCATTAATCT-CTTCAGGTGCTGTCGTGA
```
