# Supplementary material for: Decay of Genes Encoding the Oomycete Flagellar Proteome in the Downy Mildew Hyaloperonospora arabidopsidis
Source: PLoS One. 2012 Oct 15;7(10):e47624. doi: 10.1371/journal.pone.0047624 (PMC3471859; doi:10.1371/journal.pone.0047624)
Supplement: Table S2 — Features of gene remnants detected in H. arabidopsidis . (PDF) [file pone.0047624.s004.pdf]

Table S2. Features of gene remnants detected in *H. arabidopsidis*[1].

PITG NUMBER	COMMON NAME	SIZE OF *P. INFESTANS* GENE (NT)	NT OF *P. INFESTANS* GENE IN ALIGNMENT[2]	FRACTION OF *P. INFESTANS* GENE IN ALIGNMENT[2]	LALIGN SCORE[3]	PER CENT NT MATCH	FALSE DISCOVERY RATE[4]	*E* VALUE FROM PRSS (SHUFFLE)[5]
627	FBB9	714	239	0.33	178	54	5E-05	2E-26
942	DAP2	1713	371	0.22	492	62	5E-03	9E-46
1067	BBS7	2250	429	0.19	144	57	(0)	2E-25
1312	FAP155	1317	375	0.28	216	55	2E-03	6E-41
2417	DCC2	1824	769	0.42	164	51	8E-04	2E-95
2492	MOT51	6741	306	0.05	227	58	(0)	2E-34
2721	FAP267	1218	1150	0.94	268	50	3E-04	1E-125
3419	CPC1	4512	1323	0.29	396	52	2E-03	1E-132
3488	DHC6	12942	180	0.01	208	63	(0)	3E-25
3600	BBS2	2172	1662	0.77	868	53	2E-03	2E-186
3878	DIC1	2259	290	0.13	283	59	3E-03	1E-32
4600	DII6	1773	372	0.21	240	59	(0)	1E-40
4711	IFT25	417	233	0.56	228	57	9E-03	2E-28
4848	MOT28	504	226	0.45	399	62	(0)	1E-52
5487	SSA1	1683	755	0.45	369	55	5E-03	2E-91
5499	FAP58	2667	850	0.32	528	57	1E-03	4E-71
5608	FM135	2841	1883	0.66	415	51	(0)	1E-152
6195	SSA2	1689	375	0.22	196	60	1E-03	7E-17
7793	DLT2	411	192	0.47	170	58	2E-03	7E-15
8015	DLI1	1149	570	0.50	278	56	(0)	2E-36
8038	MOT25	621	174	0.28	166	59	(0)	7E-75
8685	UNC119	720	581	0.81	333	54	(0)	6E-67
10664	IFT122	3858	433	0.11	290	57	(0)	2E-44
10796	FAP250	1686	1227	0.73	241	51	1E-02	8E-85
11109	FAP57	3900	2713	0.70	261	50	2E-03	8E-57
11526	BBS9	2754	1146	0.42	630	52	(0)	9E-128
12109	MOT49	864	208	0.24	195	59	(0)	6E-23
12763	FBB4	1443	1324	0.92	472	52	(0)	7E-128
12891	MOT17	1020	592	0.58	291	54	4E-04	9E-71
13069	TAT	540	200	0.37	138	56	3E-03	6E-17
13461	DIP13	336	207	0.62	231	60	(0)	4E-22

PITG NUMBER	COMMON NAME	SIZE OF *P. INFESTANS* GENE (NT)	NT OF *P. INFESTANS* GENE IN ALIGNMENT[2]	FRACTION OF *P. INFESTANS* GENE IN ALIGNMENT[2]	LALIGN SCORE[3]	PER CENT NT MATCH	FALSE DISCOVERY RATE[4]	*E* VALUE FROM PRSS (SHUFFLE)[5]
14226	IFT46	1536	138	0.09	124	57	7E-03	4E-11
14588	MKS1	606	99	0.16	157	64	1E-03	2E-11
15414	DII1	792	83	0.10	136	64	8E-03	1E-09
16089	SSA4	621	473	0.76	271	59	1E-02	2E-34
18065	FAP66	4077	304	0.07	283	59	1E-04	2E-29
20212	IFT172	5349	2126	0.39	494	54	(0)	4E-120
20902	DLE3	714	451	0.63	261	57	1E-03	2E-46
21013	KAP	1917	1789	0.93	559	54	(0)	3E-175

[1]Gene remnants were identified by using LALIGN to search the relevant intergenic regions of *H. arabidopsidis* with *P. infestans* gene sequences, trimmed of introns. Matches with $E<0.2$ were then examined in more detail.

[2]Counts all bases in alignment, including identical and unmatched bases, but excluding gaps.

[3]Using default parameters in LALIGN (+5/-4 matrix, open/ext: -12/-4). If separate segments were generated by LALIGN, the score reflects the highest value.

[4]Determined by searching 7×10^6 nt of sequences from *H. arabidopsidis,* joined from 1000-nt regions of DNA downstream of 7000 genes, with each *P. infestans* sequence. The number of matches obtained using the threshold employed for gene remnant detection ($E<0.2$), minus authentic hits (if any) against remnants, was multiplied by the size of the *H. arabidopsidis* sequence originally searched for remnants and divided by 7×10^6. If no hits were detected, a rate of "(0)" is reported.

[5]The statistical significance of alignments was estimated by employing the program PRSS (http://fasta.bioch.virginia.edu/fasta_www2/fasta_www.cgi?rm=shuffle), using 200 shuffles of the *H. arabidopsidis* sequence. If separate segments were generated by LALIGN, the *E* value is shown for the PRSS analysis of the LALIGN alignment with the highest score.
